# Correction: ZC3H11A mutations cause high myopia by triggering PI3K-AKT and NF-κB-mediated signaling pathway in humans and mice

**DOI:** 10.7554/eLife.110124

**Published:** 2025-12-01

**Authors:** Chong Chen, Qian Liu, Cheng Tang, Yu Rong, Xinyi Zhao, Dandan Li, Fan Lu, Jia Qu, Xinting Liu

**Keywords:** Mouse

Chen C, Liu Q, Tang C, Rong Y, Zhao X, Li D, Lu F, Qu J, Liu X. 2025. ZC3H11A mutations cause high myopia by triggering PI3K-AKT and NF-κB-mediated signaling pathway in humans and mice. *eLife* **12**:RP91289. doi: 10.7554/eLife.91289.

Published 27 August 2025

During our verification process, we identified duplicated schematic representations of the electroretinogram (ERG) traces under 0.01 cds/m² conditions between the Het and WT groups in Figure 3a. This error occurred during the figure preparation process: while using the 0.01 cds/m² Het image as a reference template to maintain color and size consistency for tracing the WT image, the reference image was inadvertently retained in the final version, resulting in duplicated Het images. We emphasize that this issue only affects the visual presentation of the schematic diagrams; all statistical data and experimental results remain accurate, and it does not alter the scientific conclusions or related interpretations of the study. The figure has been corrected accordingly, and the updated Figure 3a is shown below.

The corrected Figure 3 (updated for panel A) is shown here:

**Figure 1:**
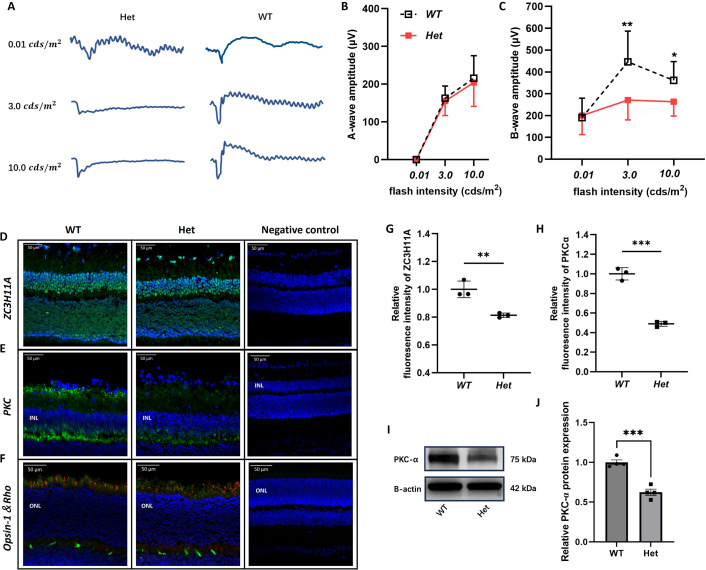


The originally published Figure 3 is shown for reference:

**Figure 2:**
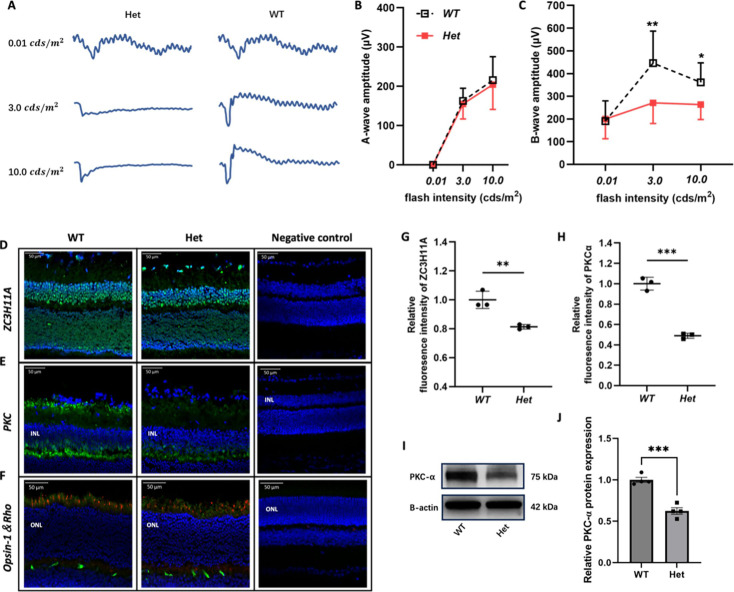


The article has been corrected accordingly.

