# Supplementary figures and images for: ZC3H11A mutations cause high myopia by triggering PI3K-AKT and NF-κB-mediated signaling pathway in humans and mice
Source: eLife. 2025 Aug 27;12:RP91289. doi: 10.7554/eLife.91289 (PMC12387752; doi:10.7554/eLife.91289)

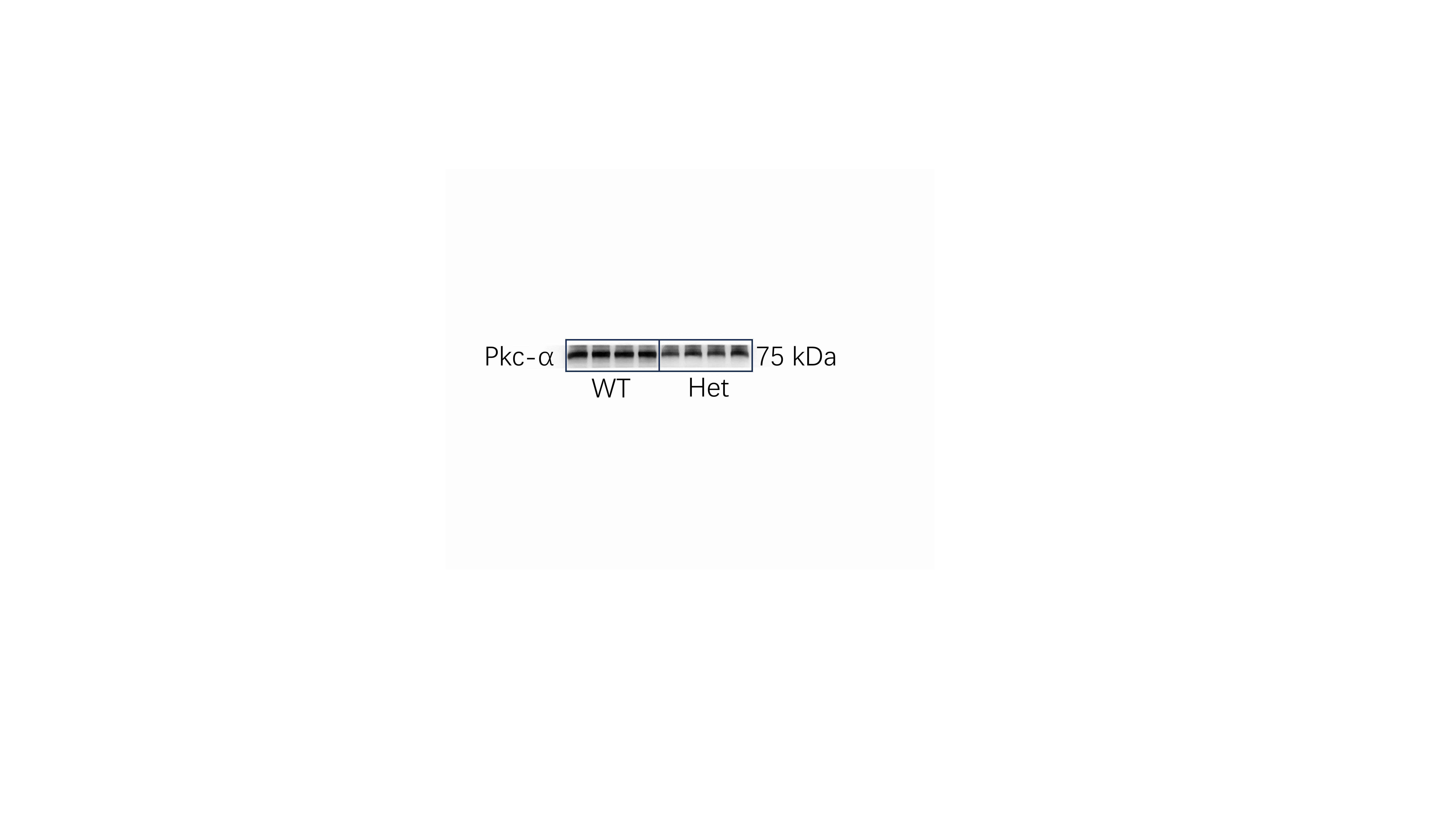

Supplement: Figure 3—source data 2. [file elife-91289-fig3-data2.zip › Figure 3—source data 2/Pkc-α.TIF]

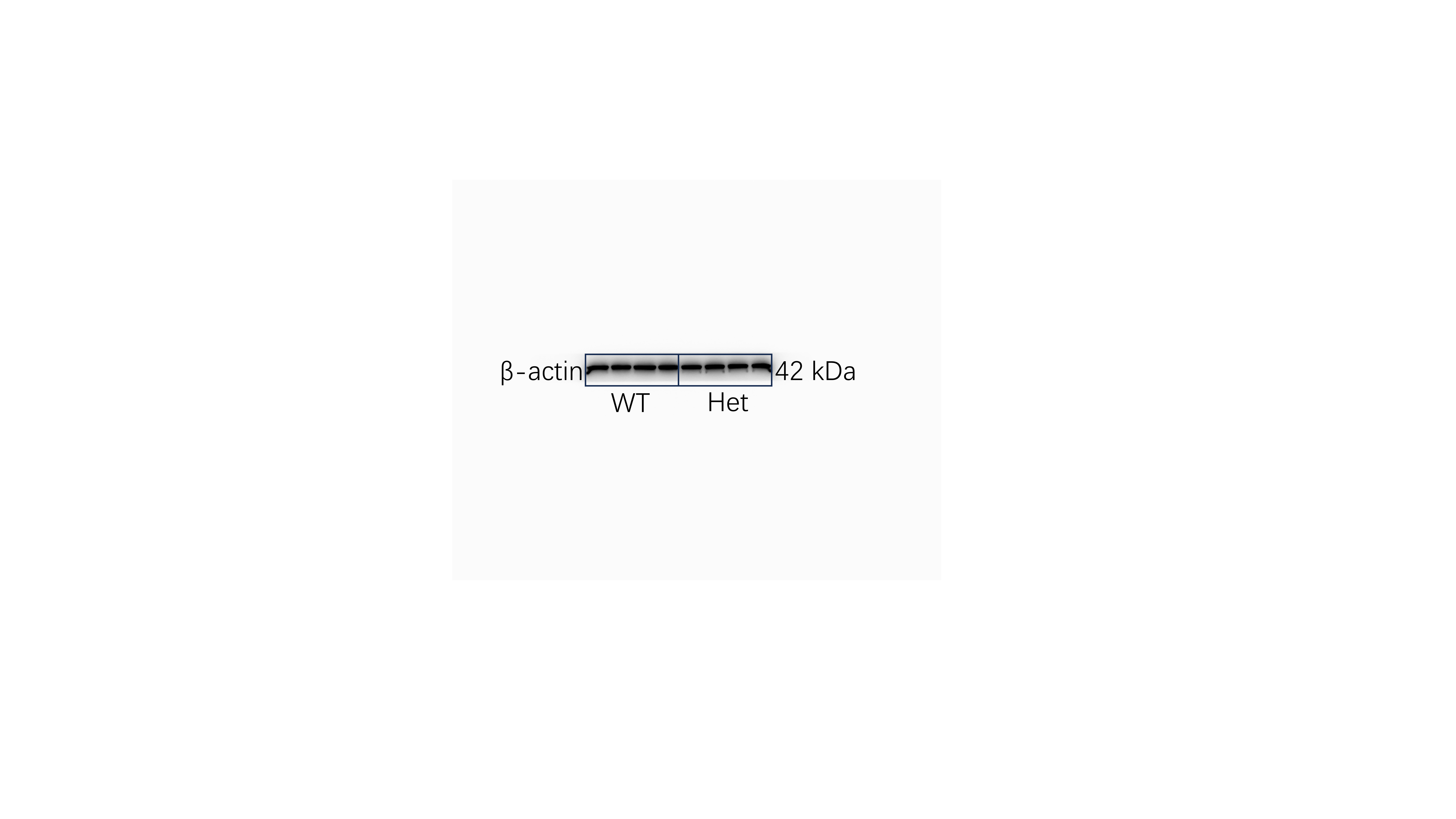

Supplement: Figure 3—source data 2. [file elife-91289-fig3-data2.zip › Figure 3—source data 2/β-actin.TIF]

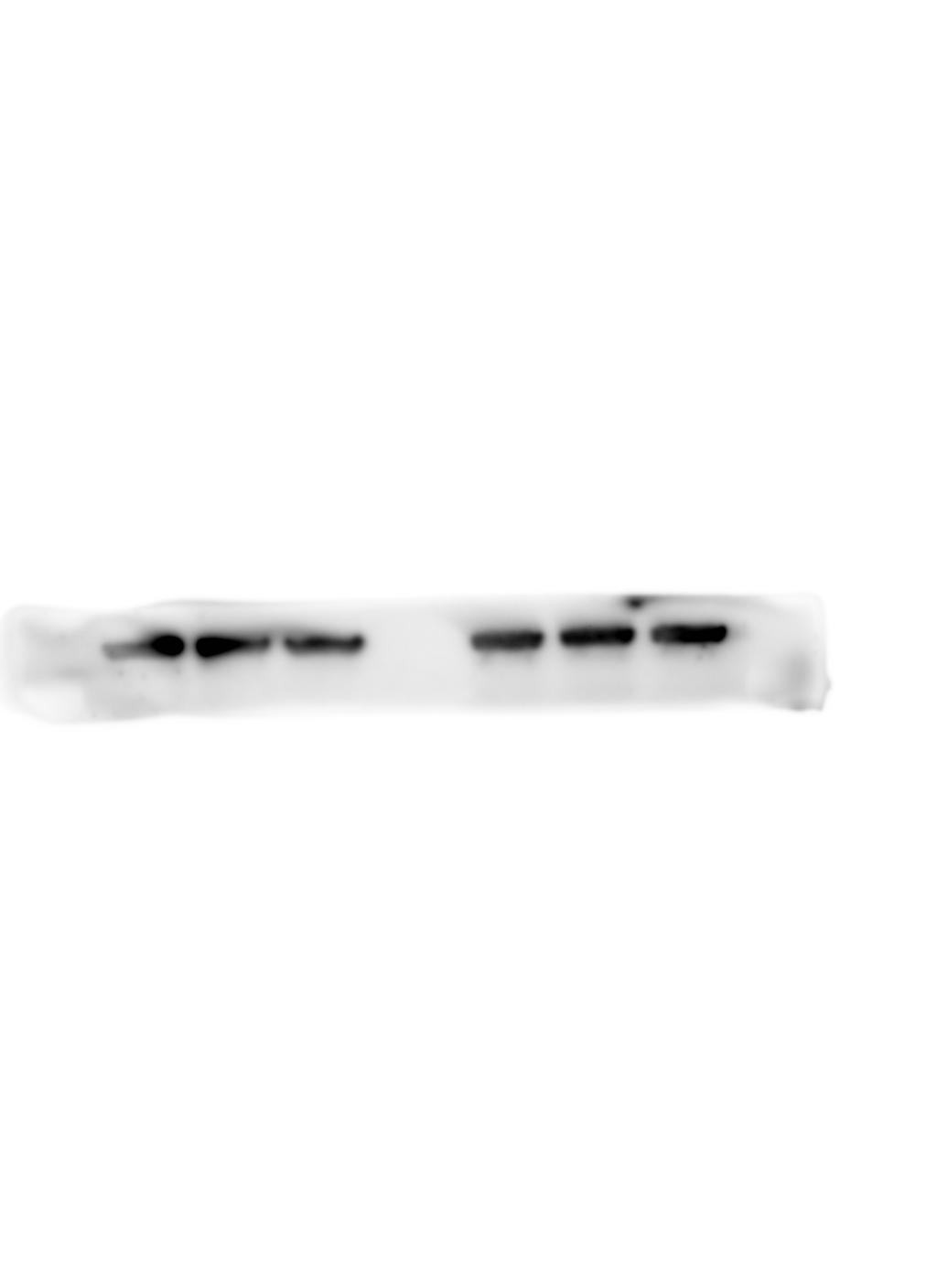

Supplement: Figure 6—source data 1. [file elife-91289-fig6-data1.zip › Figure 6-source data 1/GAPDH-1.jpg]

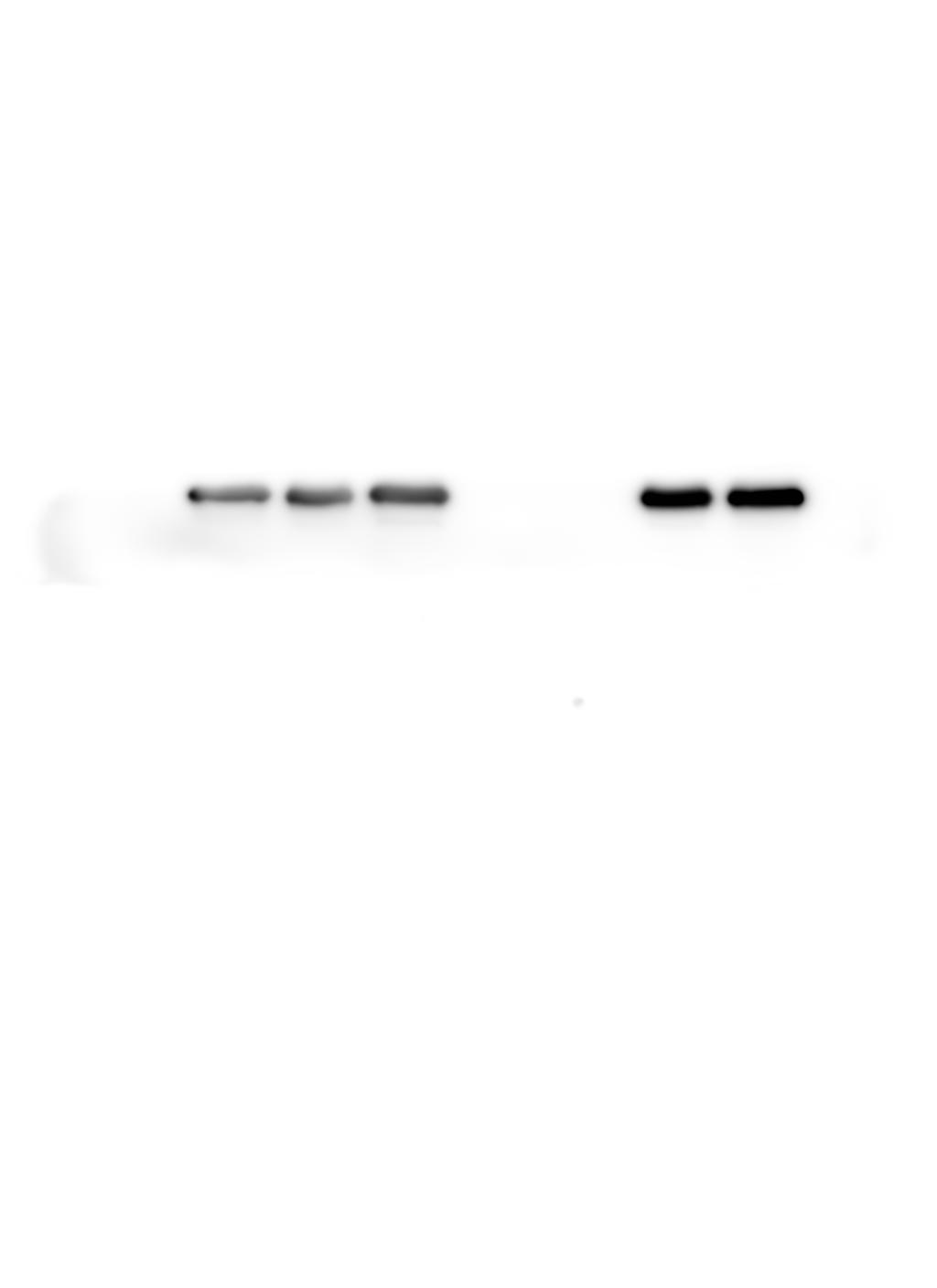

Supplement: Figure 6—source data 1. [file elife-91289-fig6-data1.zip › Figure 6-source data 1/GAPDH-2.jpg]

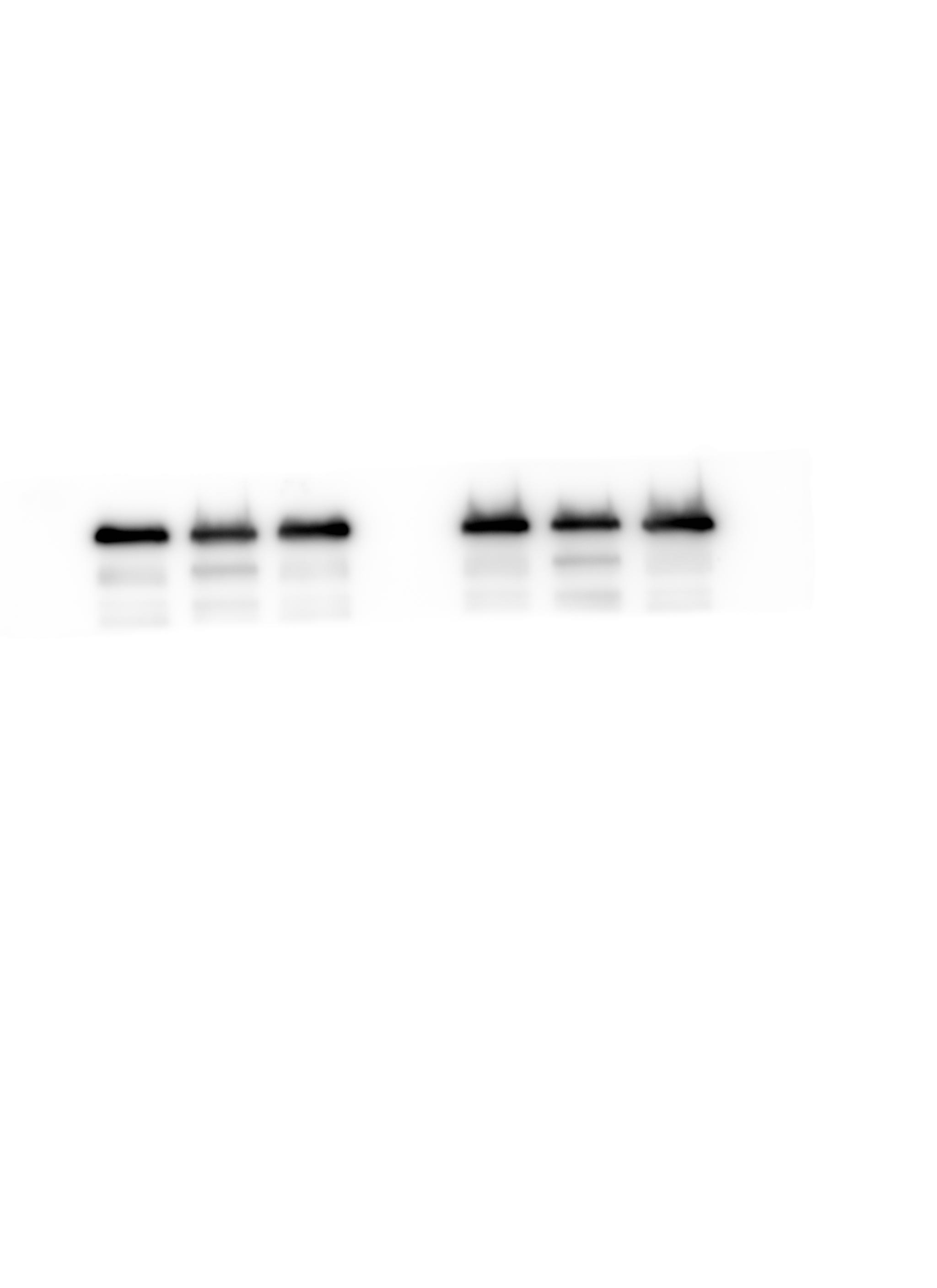

Supplement: Figure 6—source data 1. [file elife-91289-fig6-data1.zip › Figure 6-source data 1/Zc3h11a-1.jpg]

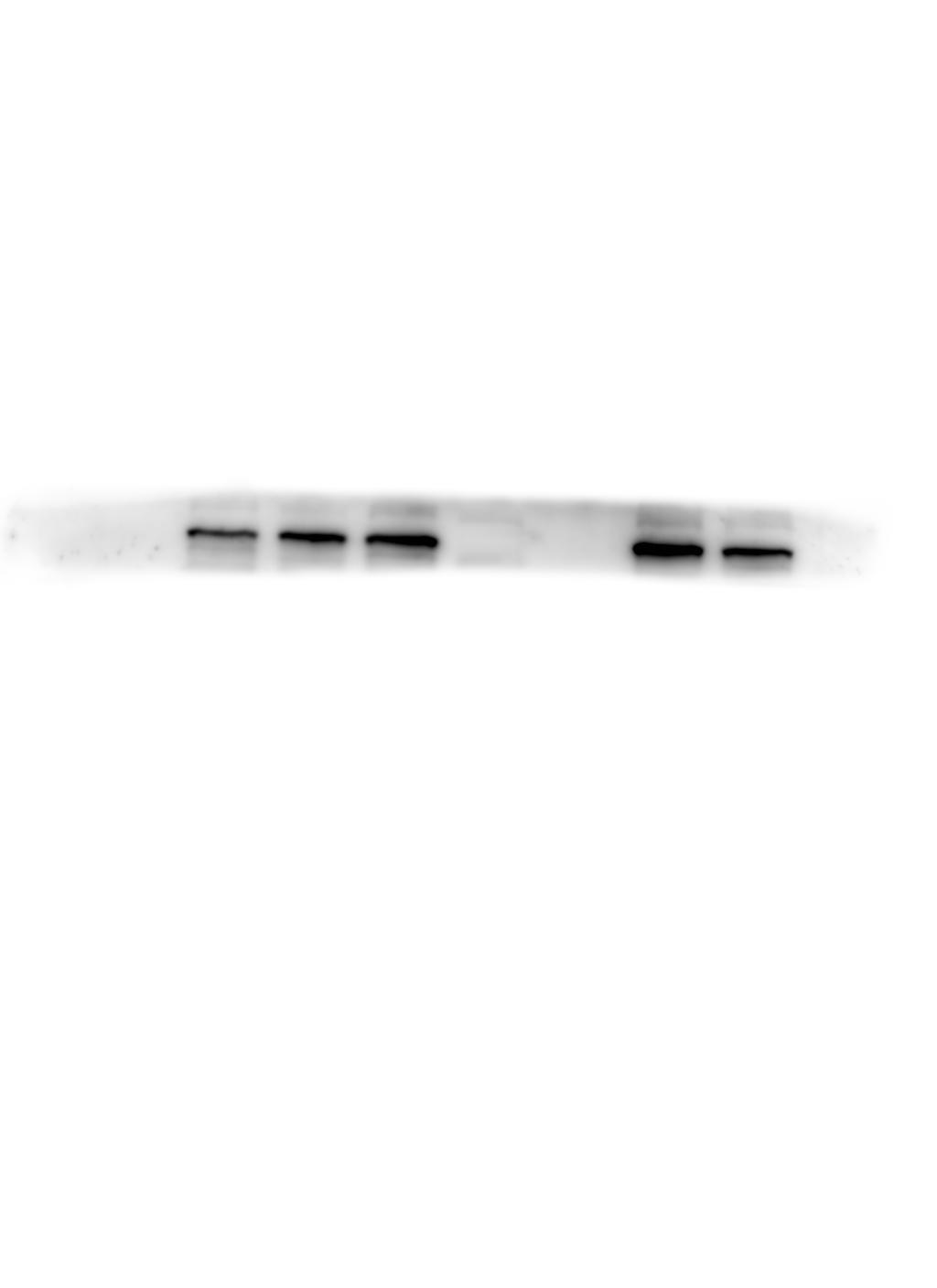

Supplement: Figure 6—source data 1. [file elife-91289-fig6-data1.zip › Figure 6-source data 1/Zc3h11a-2.jpg]

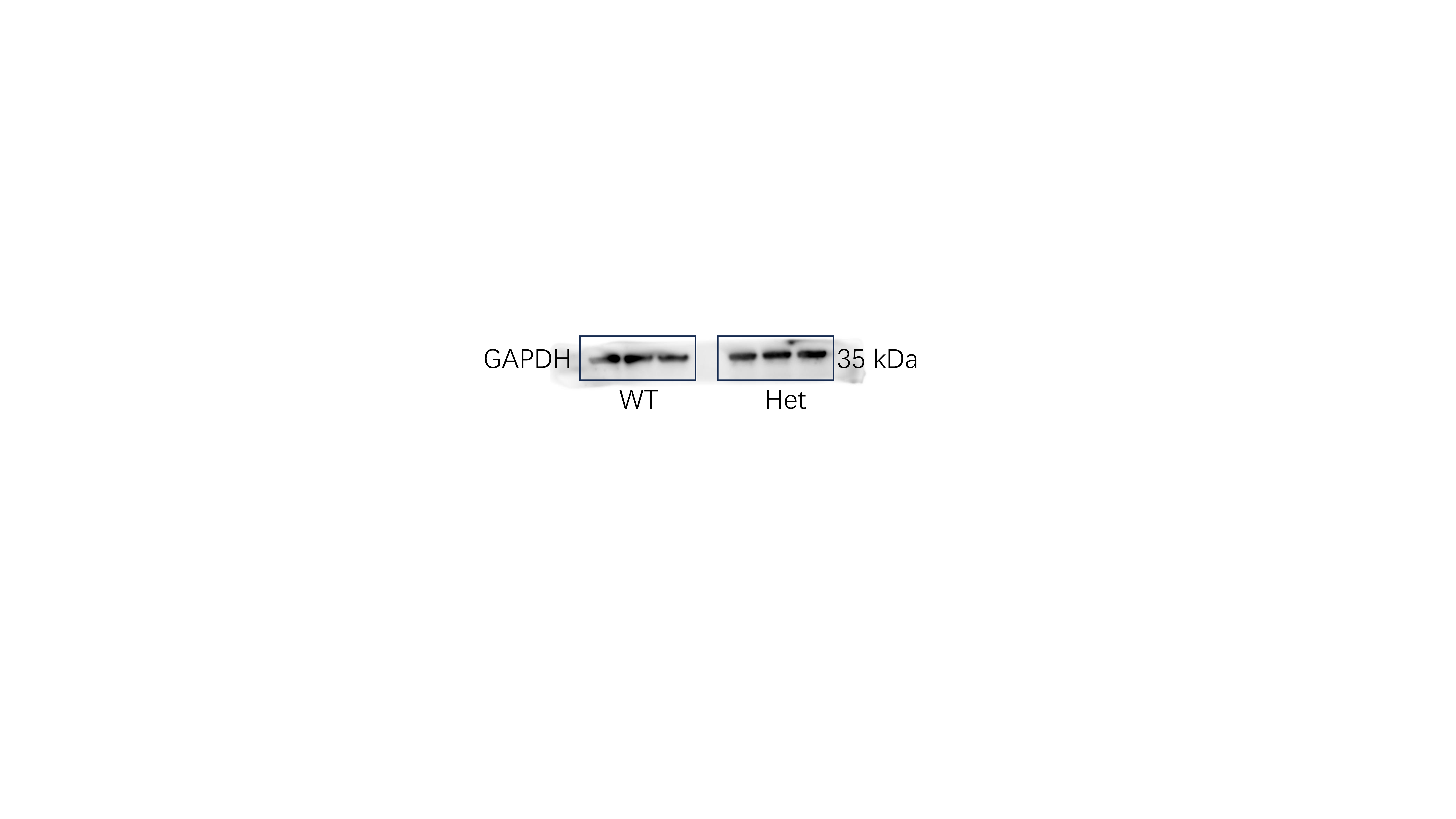

Supplement: Figure 6—source data 2. [file elife-91289-fig6-data2.zip › Figure 6-source data 2/GAPDH-1.TIF]

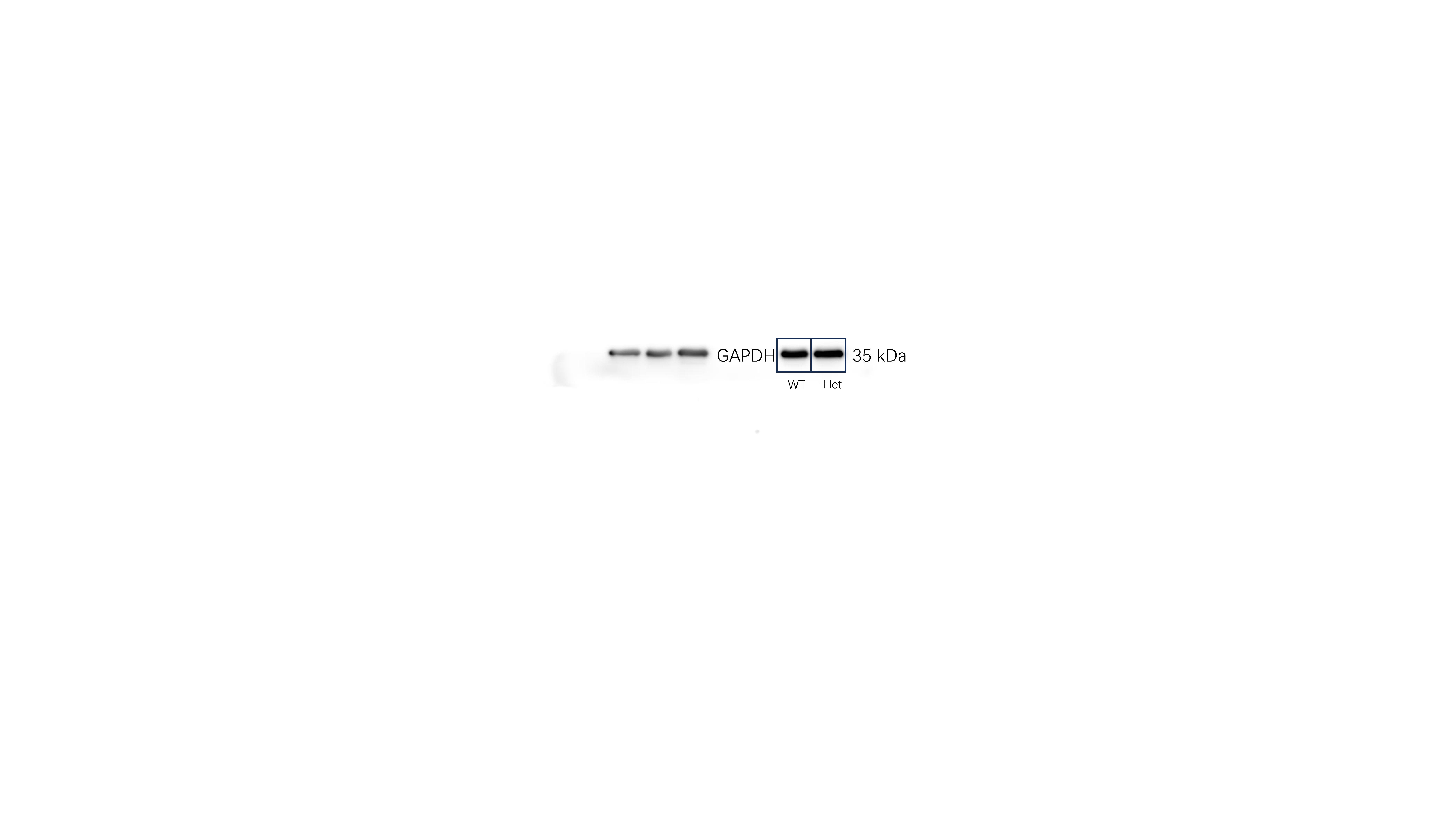

Supplement: Figure 6—source data 2. [file elife-91289-fig6-data2.zip › Figure 6-source data 2/GAPDH-2.TIF]

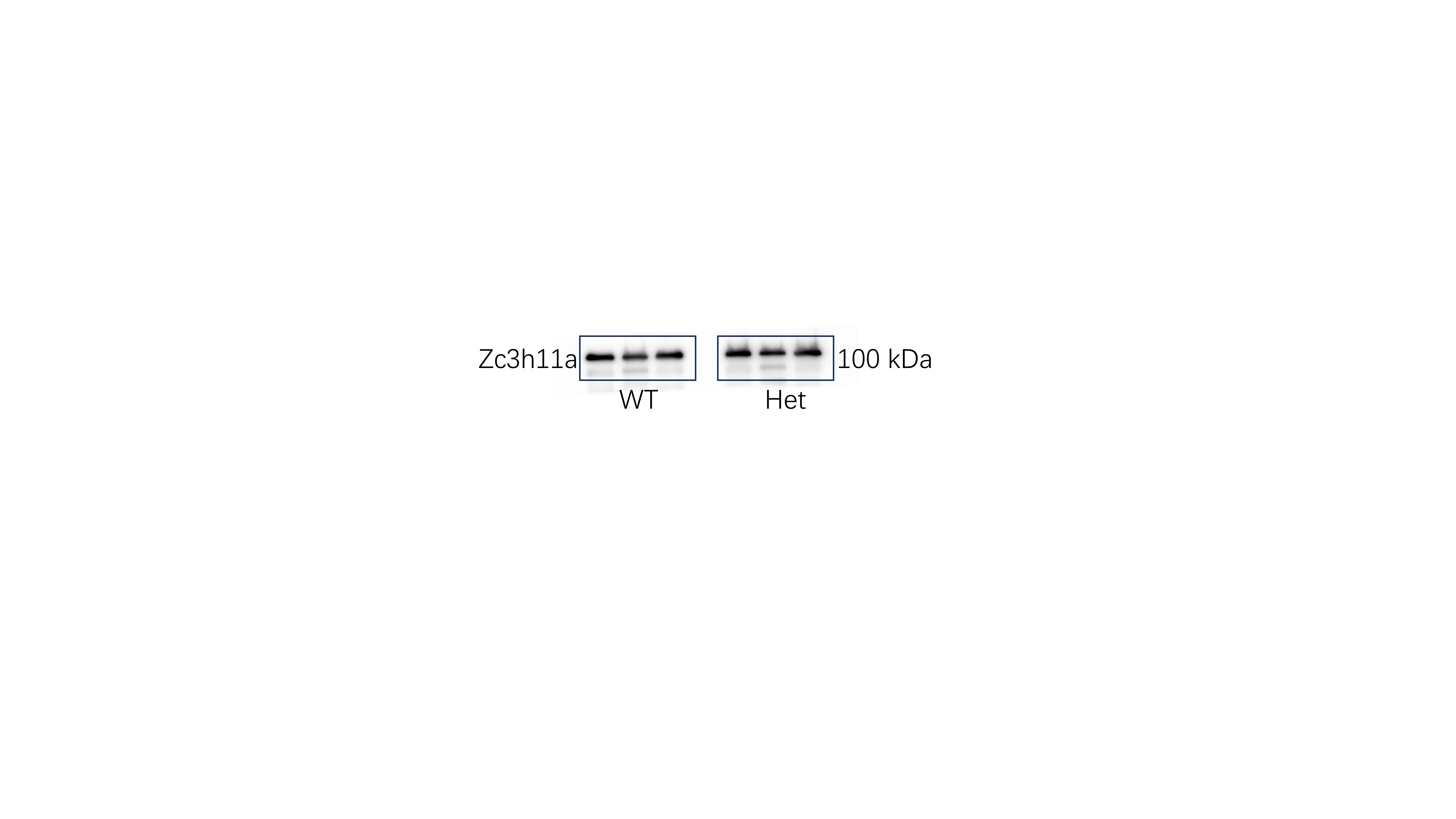

Supplement: Figure 6—source data 2. [file elife-91289-fig6-data2.zip › Figure 6-source data 2/Zc3h11a-1.TIF]

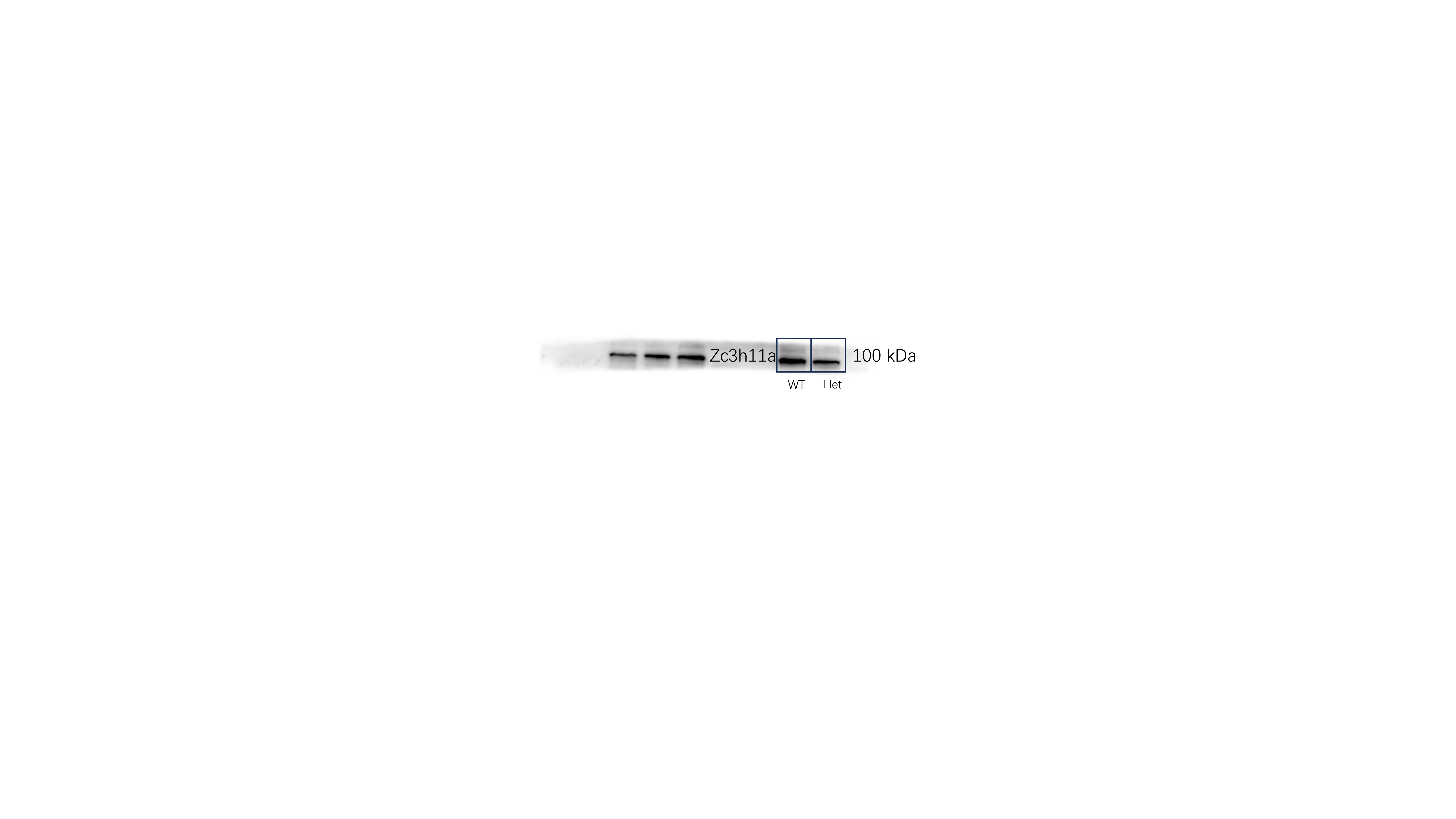

Supplement: Figure 6—source data 2. [file elife-91289-fig6-data2.zip › Figure 6-source data 2/Zc3h11a-2.TIF]

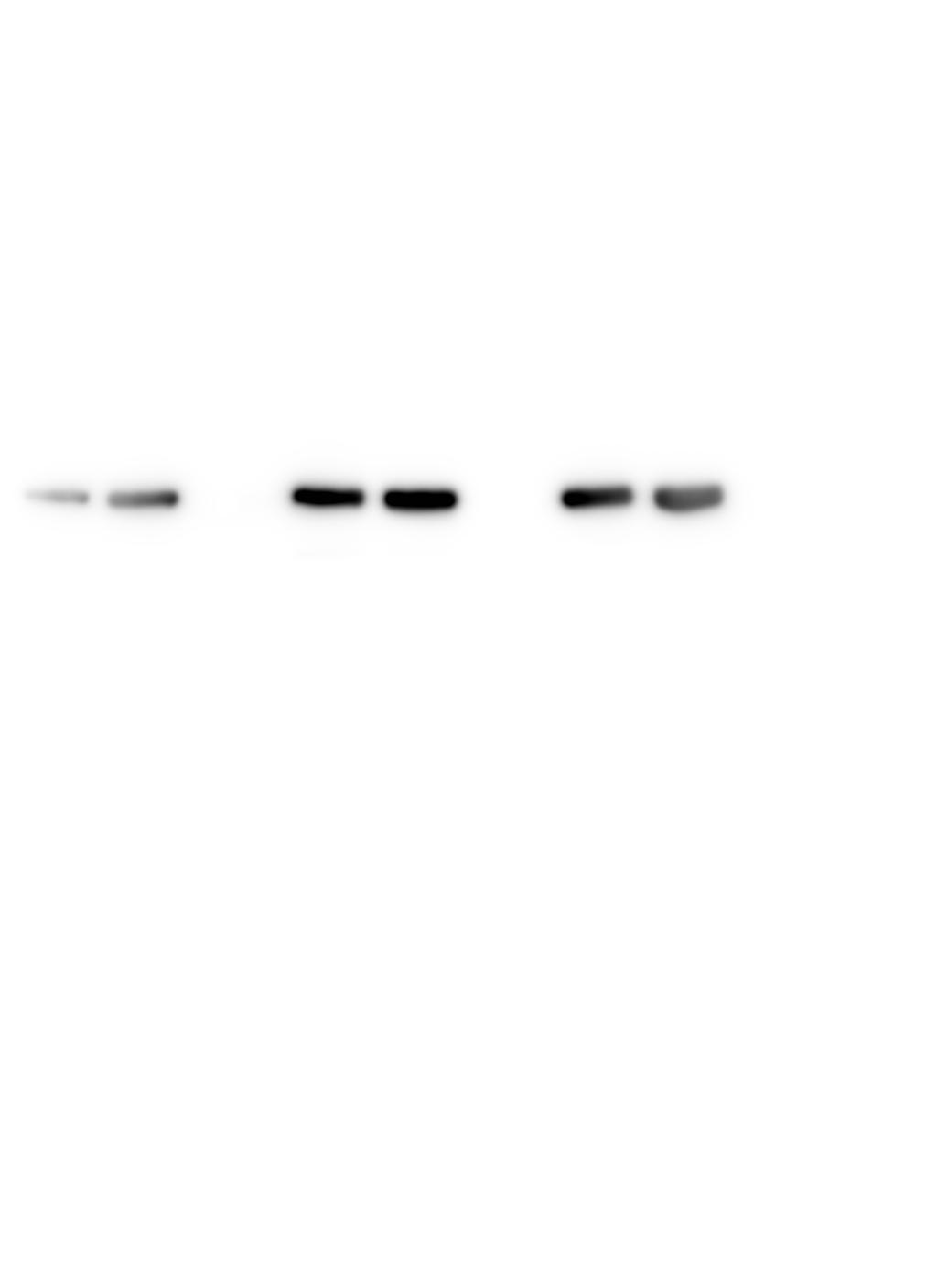

Supplement: Figure 6—source data 3. [file elife-91289-fig6-data3.zip › Figure 6-source data 3/GAPDH-1.jpg]

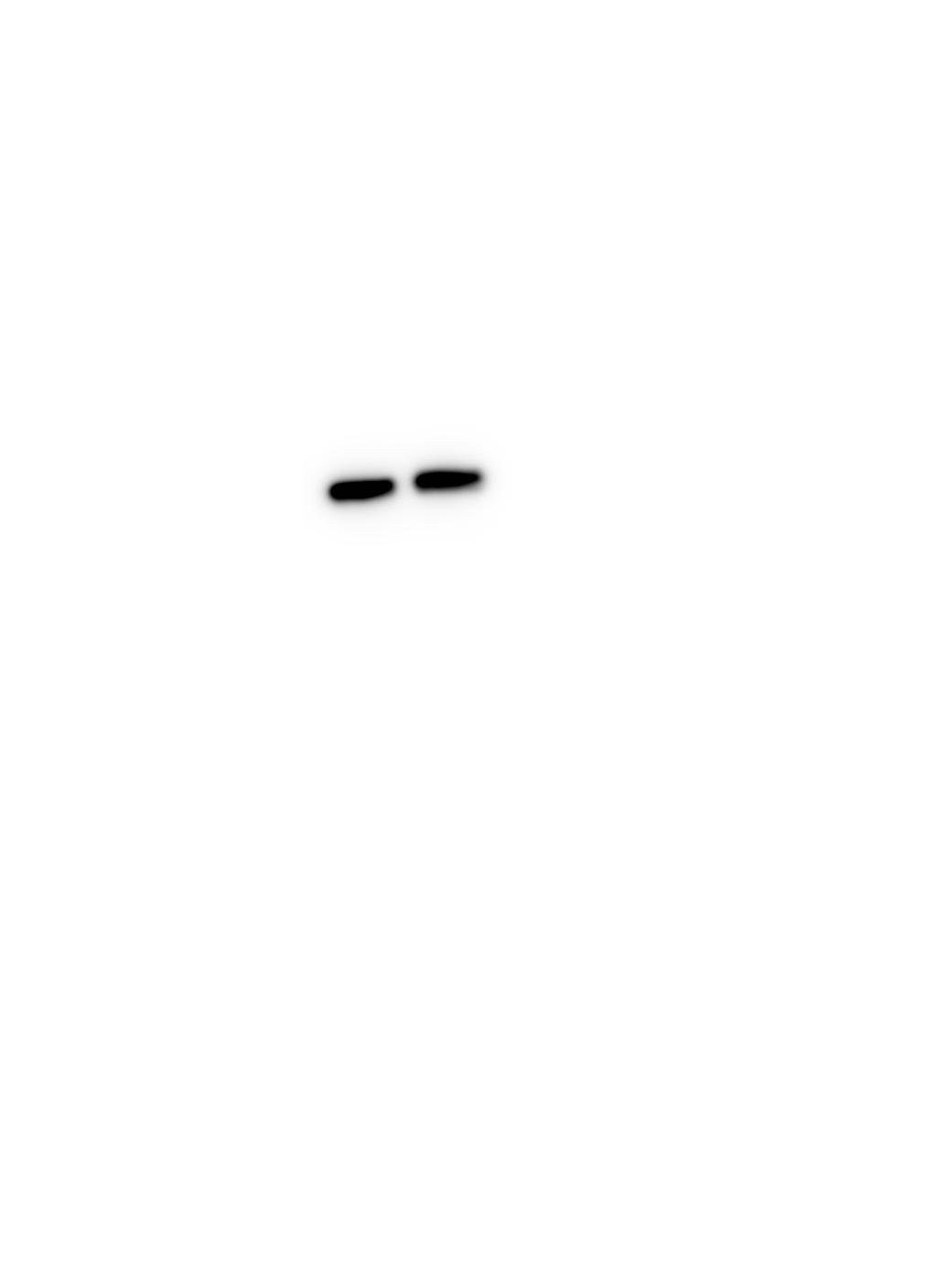

Supplement: Figure 6—source data 3. [file elife-91289-fig6-data3.zip › Figure 6-source data 3/GAPDH-2.jpg]

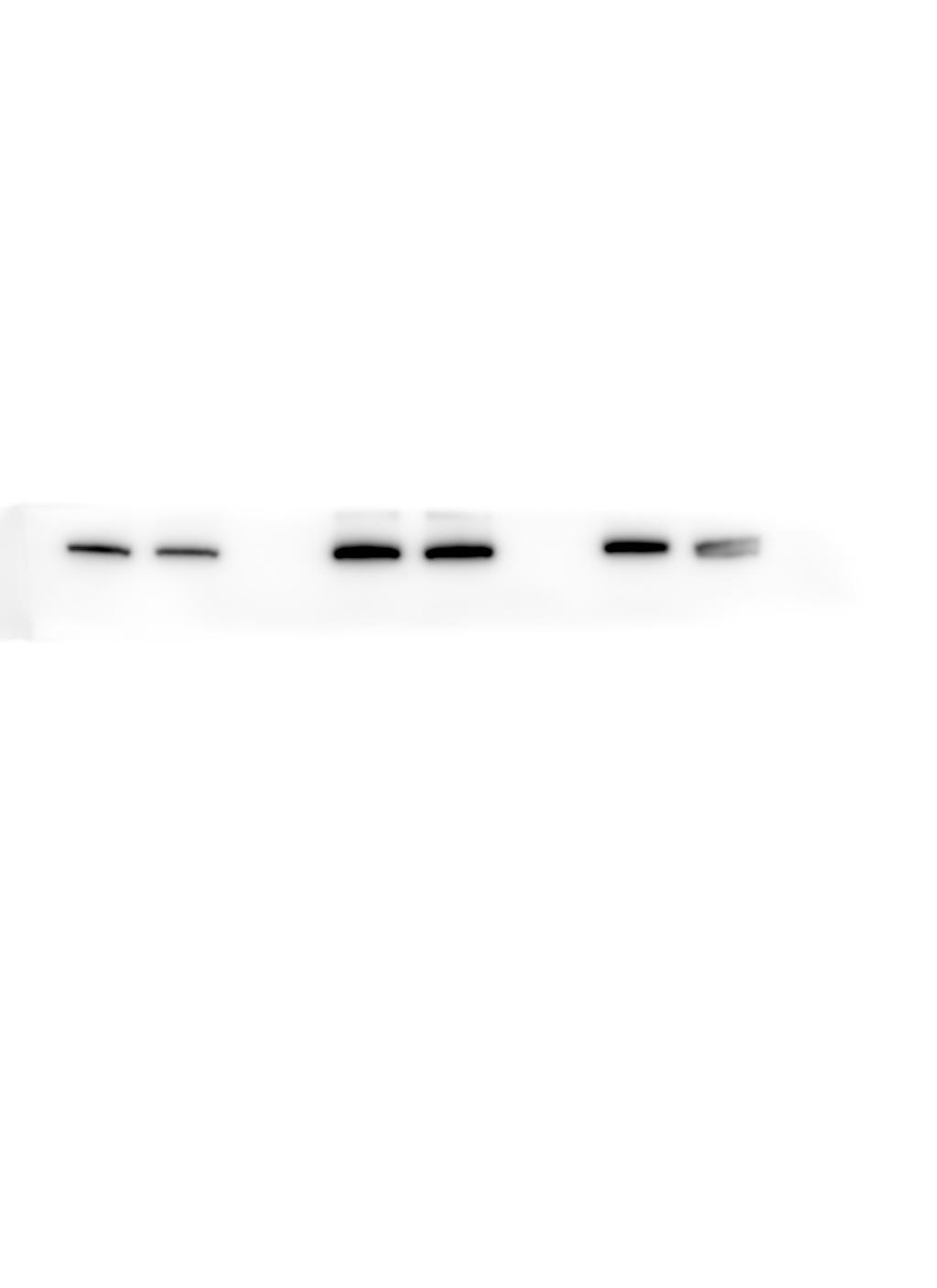

Supplement: Figure 6—source data 3. [file elife-91289-fig6-data3.zip › Figure 6-source data 3/PI3K-1.jpg]

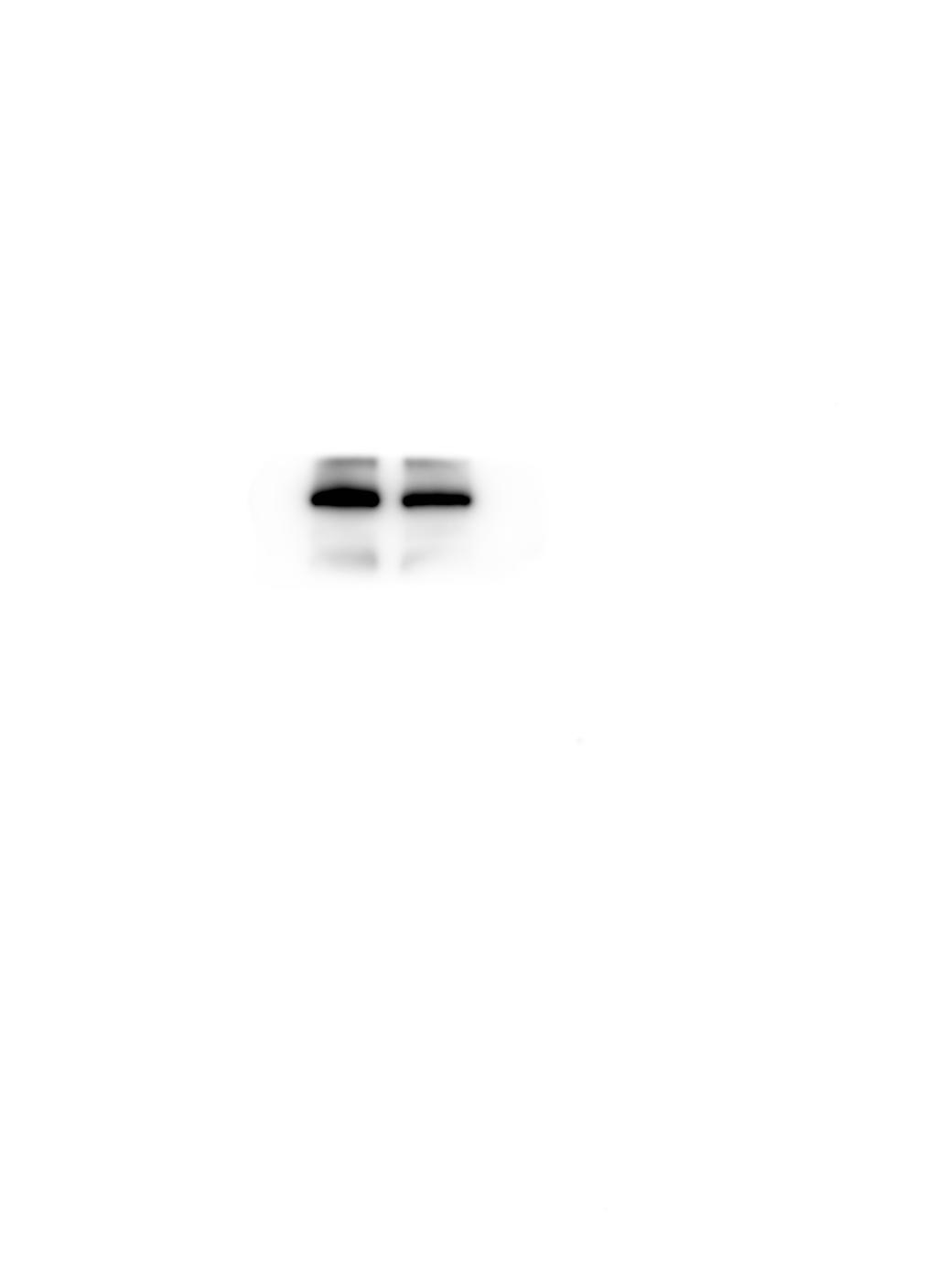

Supplement: Figure 6—source data 3. [file elife-91289-fig6-data3.zip › Figure 6-source data 3/PI3K-2.jpg]

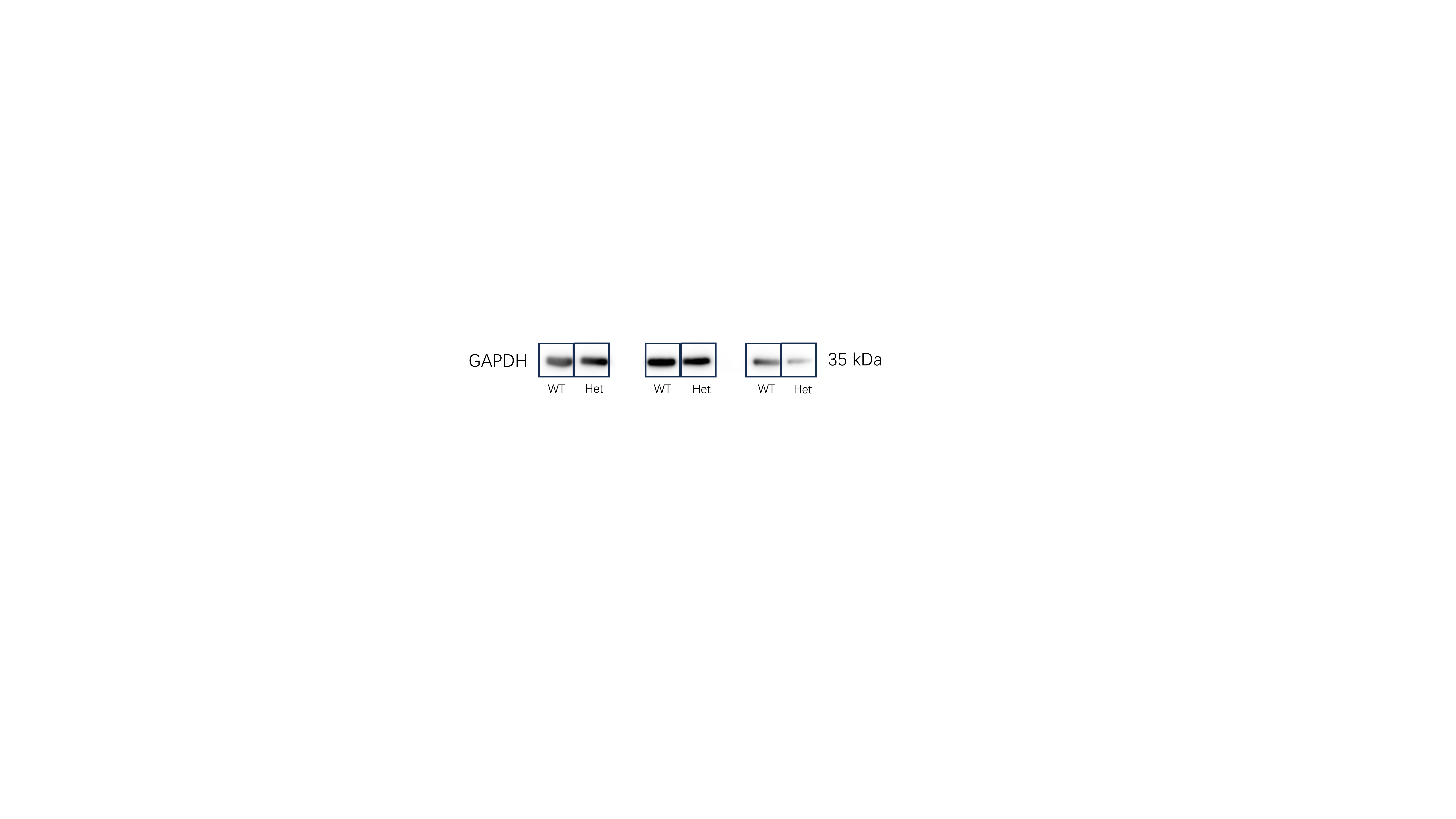

Supplement: Figure 6—source data 4. [file elife-91289-fig6-data4.zip › Figure 6-source data 4/GAPDH-1.TIF]

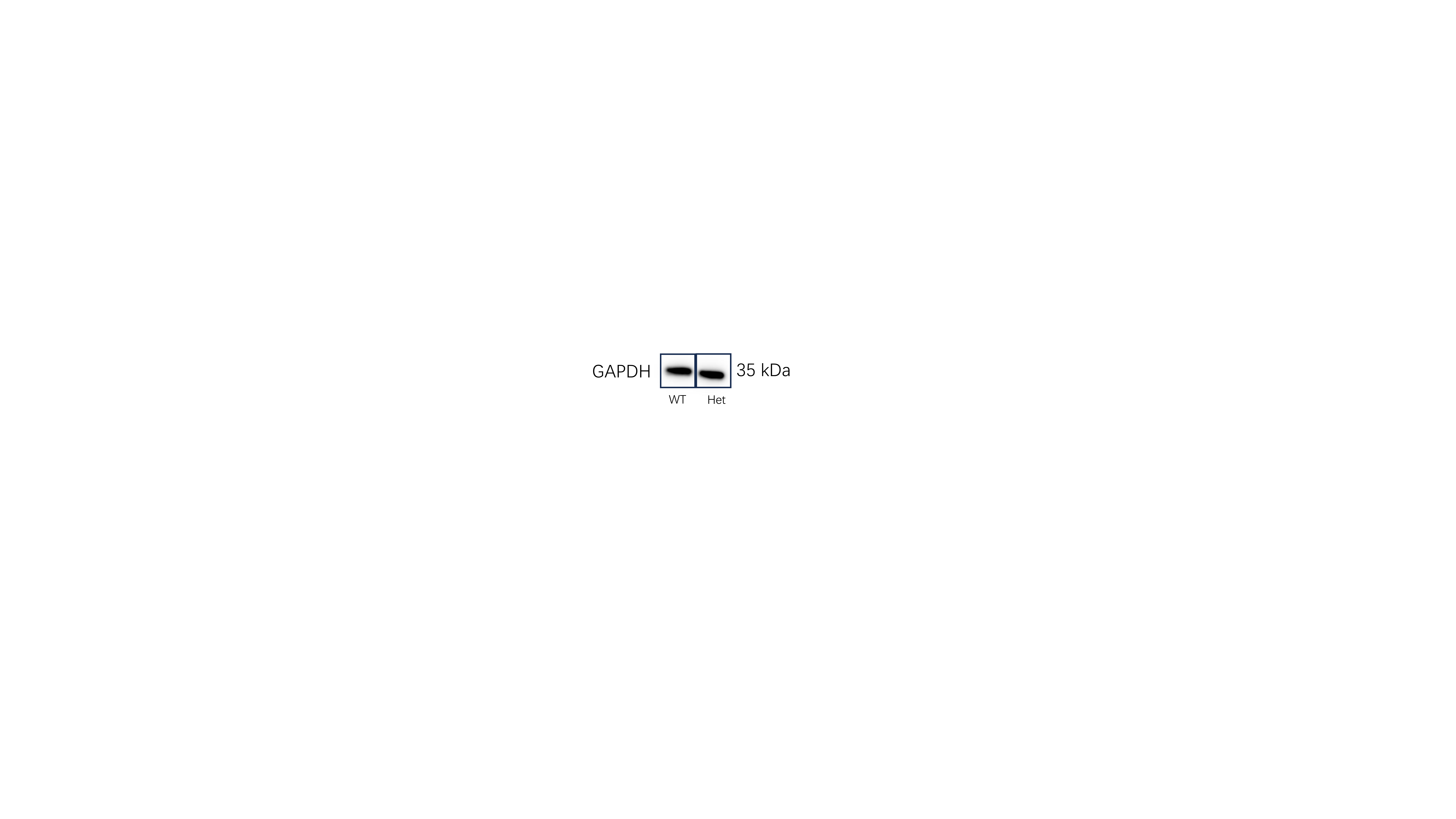

Supplement: Figure 6—source data 4. [file elife-91289-fig6-data4.zip › Figure 6-source data 4/GAPDH-2.TIF]

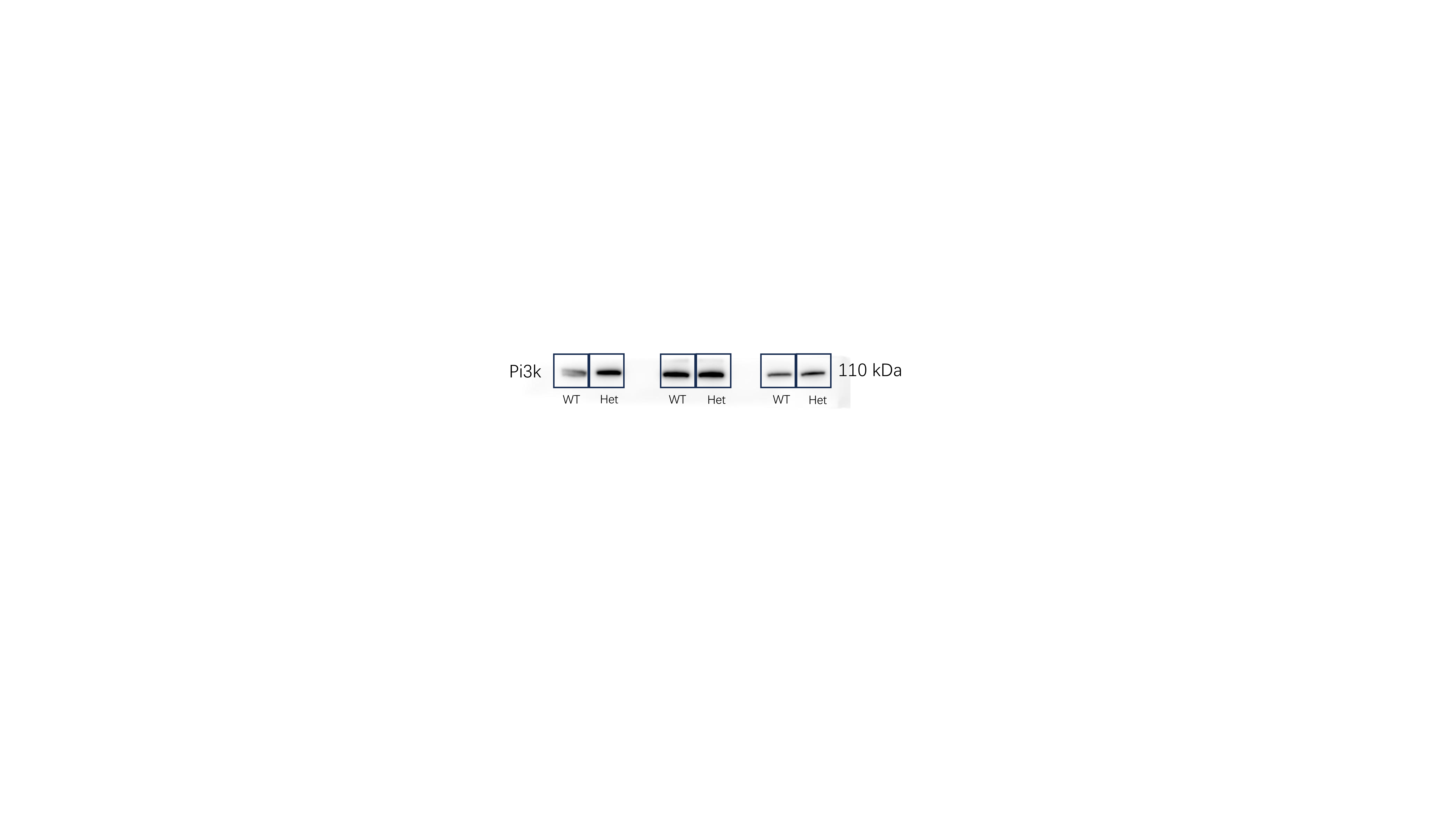

Supplement: Figure 6—source data 4. [file elife-91289-fig6-data4.zip › Figure 6-source data 4/PI3K-1.TIF]

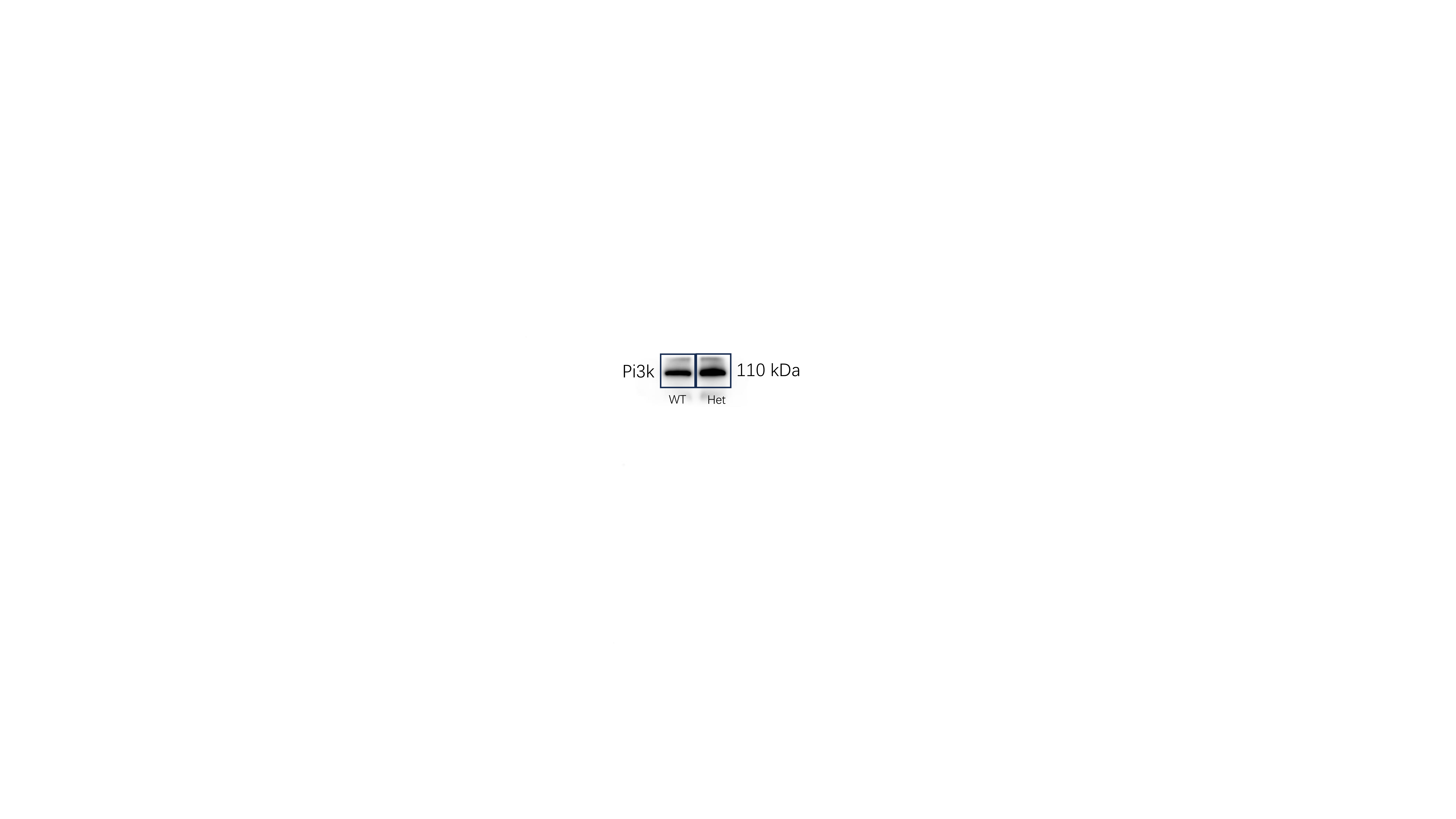

Supplement: Figure 6—source data 4. [file elife-91289-fig6-data4.zip › Figure 6-source data 4/PI3K-2.TIF]

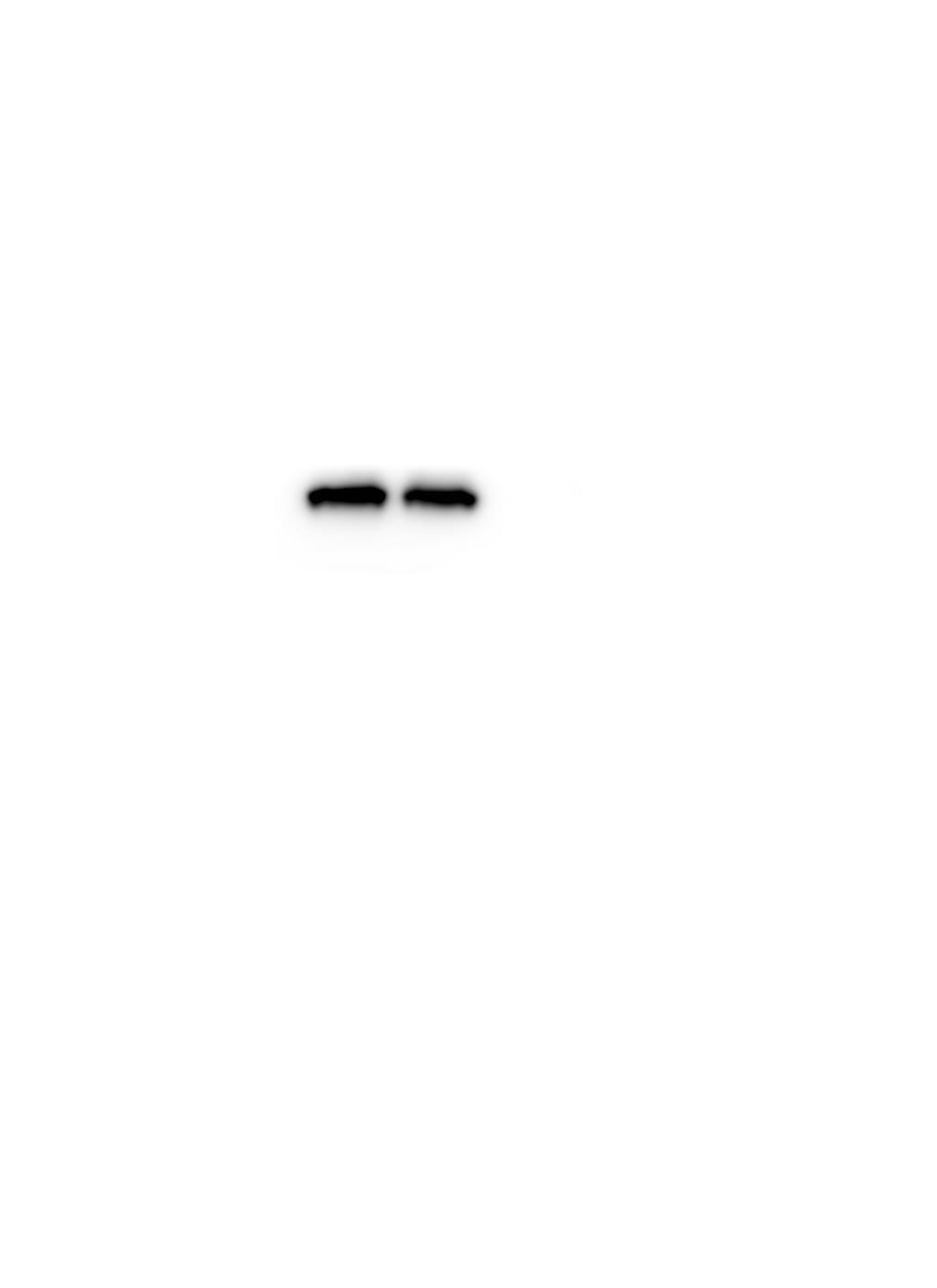

Supplement: Figure 6—source data 5. [file elife-91289-fig6-data5.zip › Figure 6-source data 5/AKT-1.jpg]

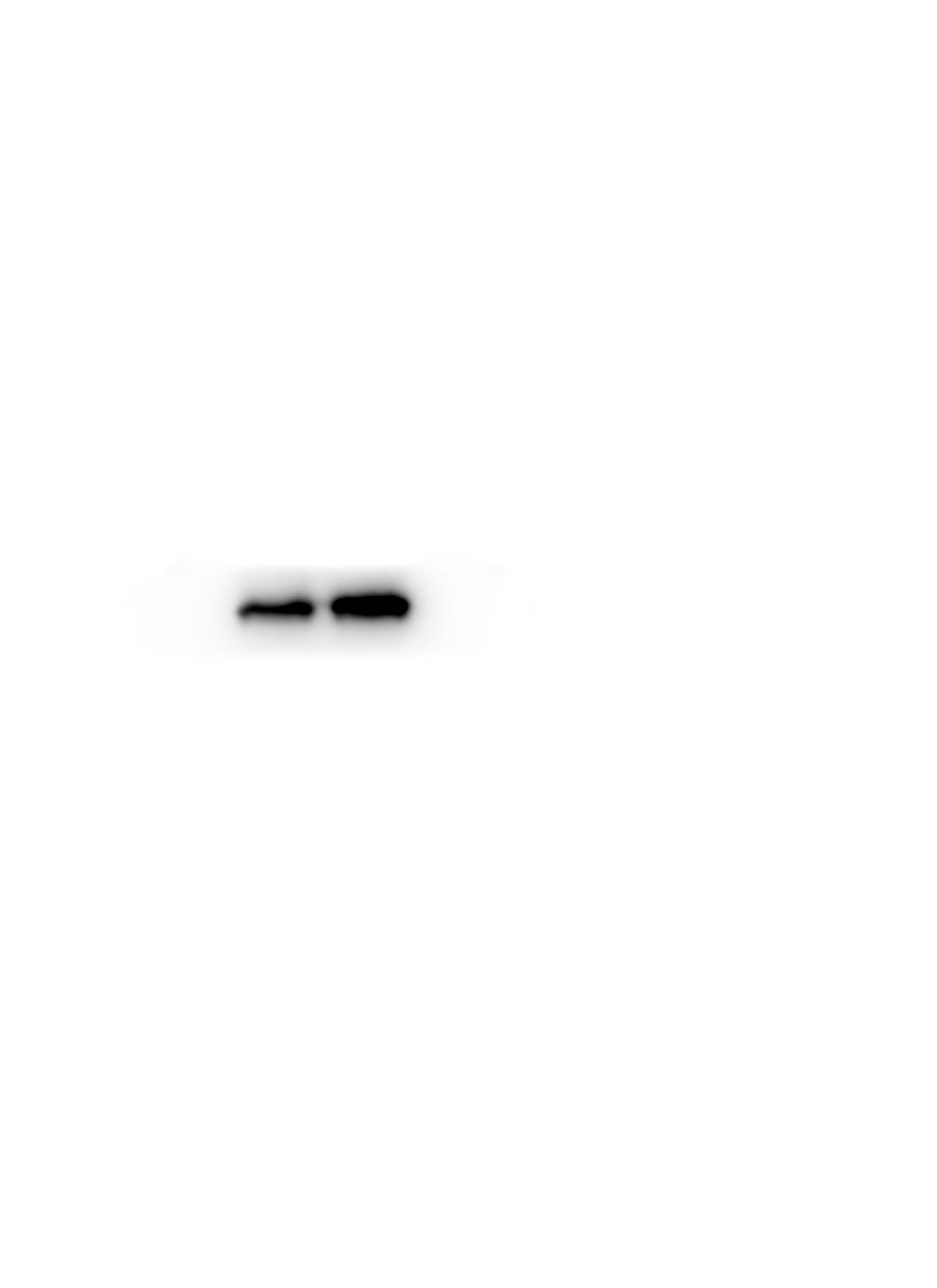

Supplement: Figure 6—source data 5. [file elife-91289-fig6-data5.zip › Figure 6-source data 5/AKT-2.jpg]

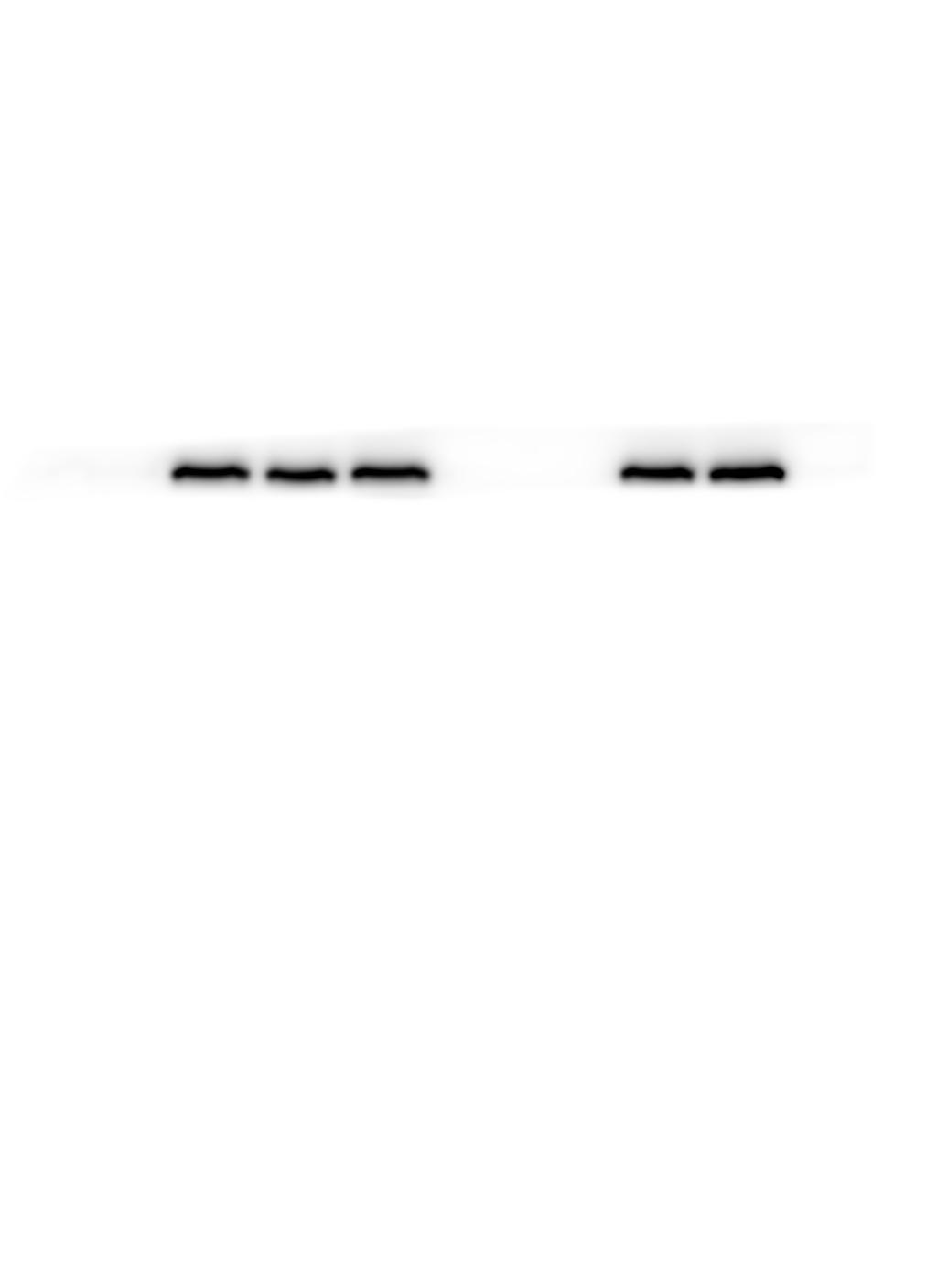

Supplement: Figure 6—source data 5. [file elife-91289-fig6-data5.zip › Figure 6-source data 5/AKT-3.jpg]

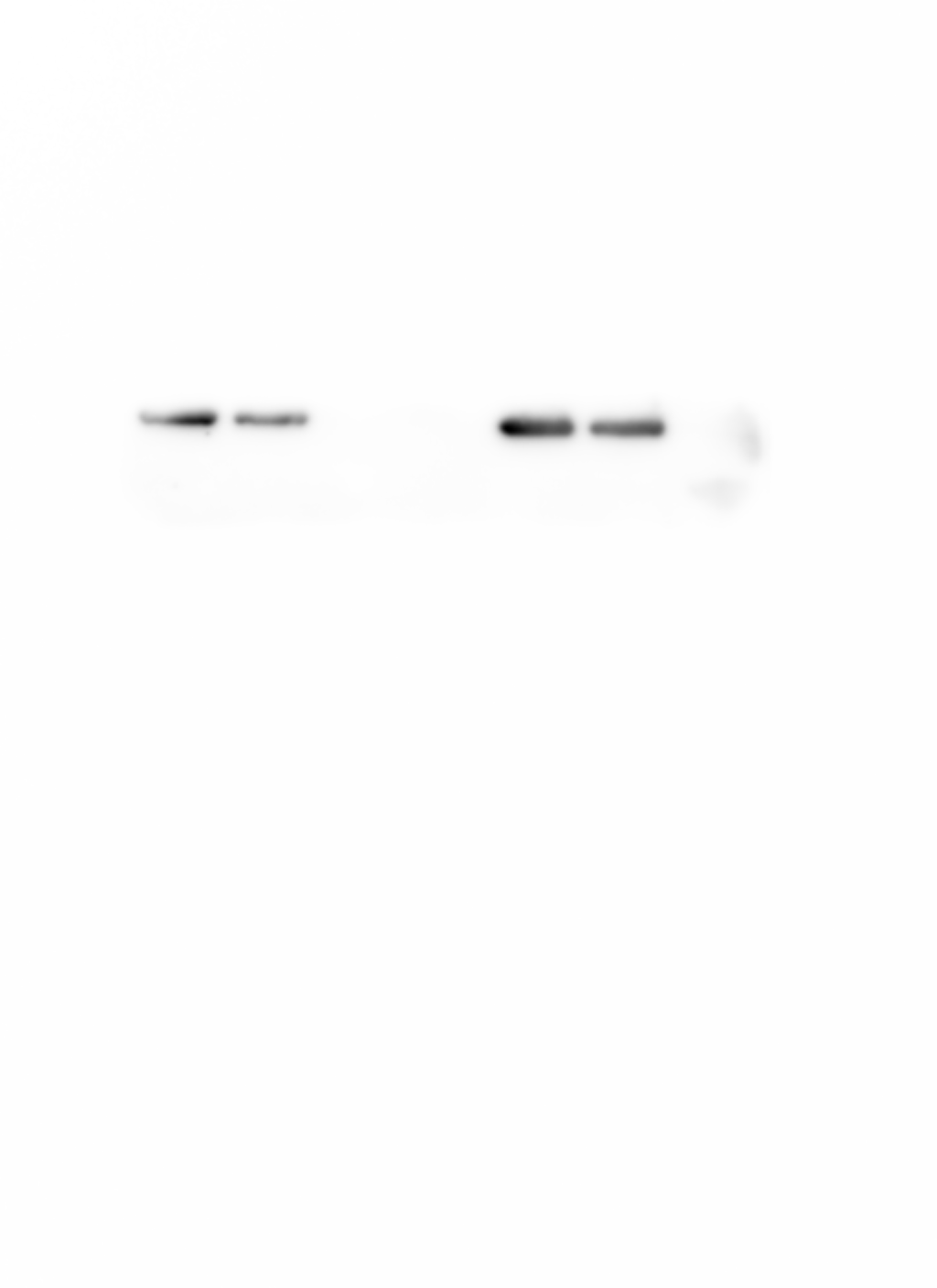

Supplement: Figure 6—source data 5. [file elife-91289-fig6-data5.zip › Figure 6-source data 5/GAPDH-1.tif]

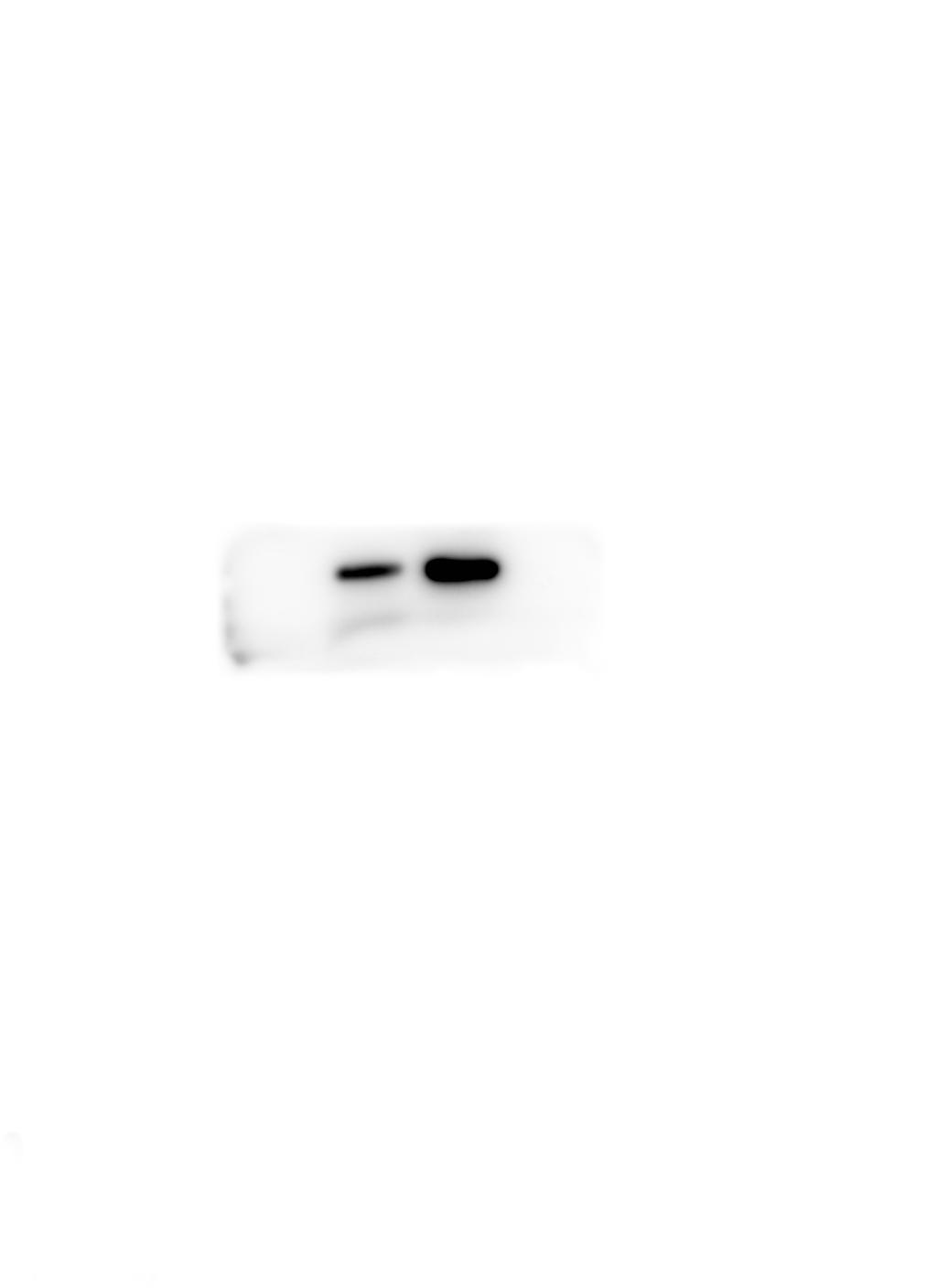

Supplement: Figure 6—source data 5. [file elife-91289-fig6-data5.zip › Figure 6-source data 5/GAPDH-2.jpg]

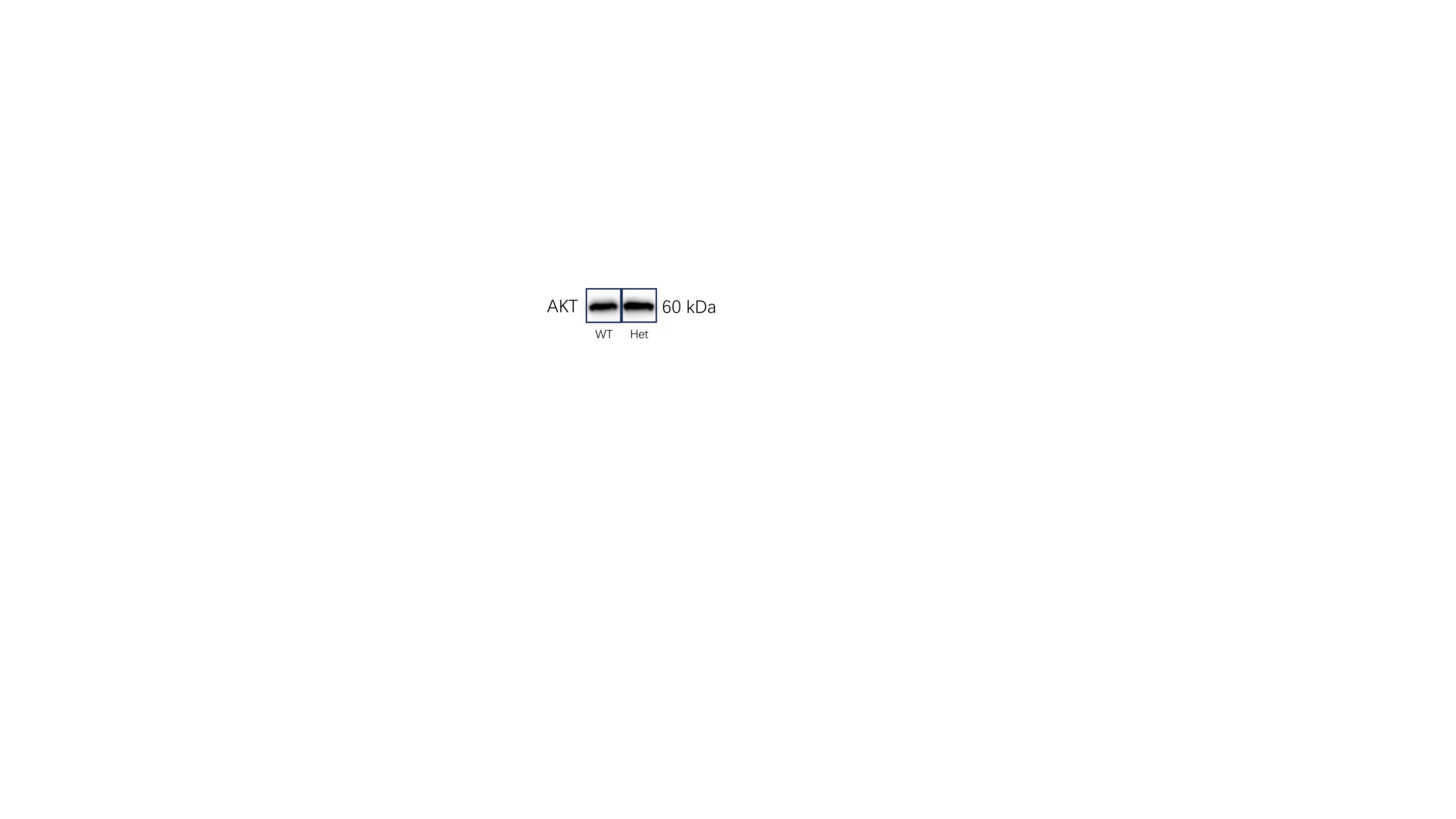

Supplement: Figure 6—source data 6. [file elife-91289-fig6-data6.zip › Figure 6-source data 6/AKT-1.TIF]

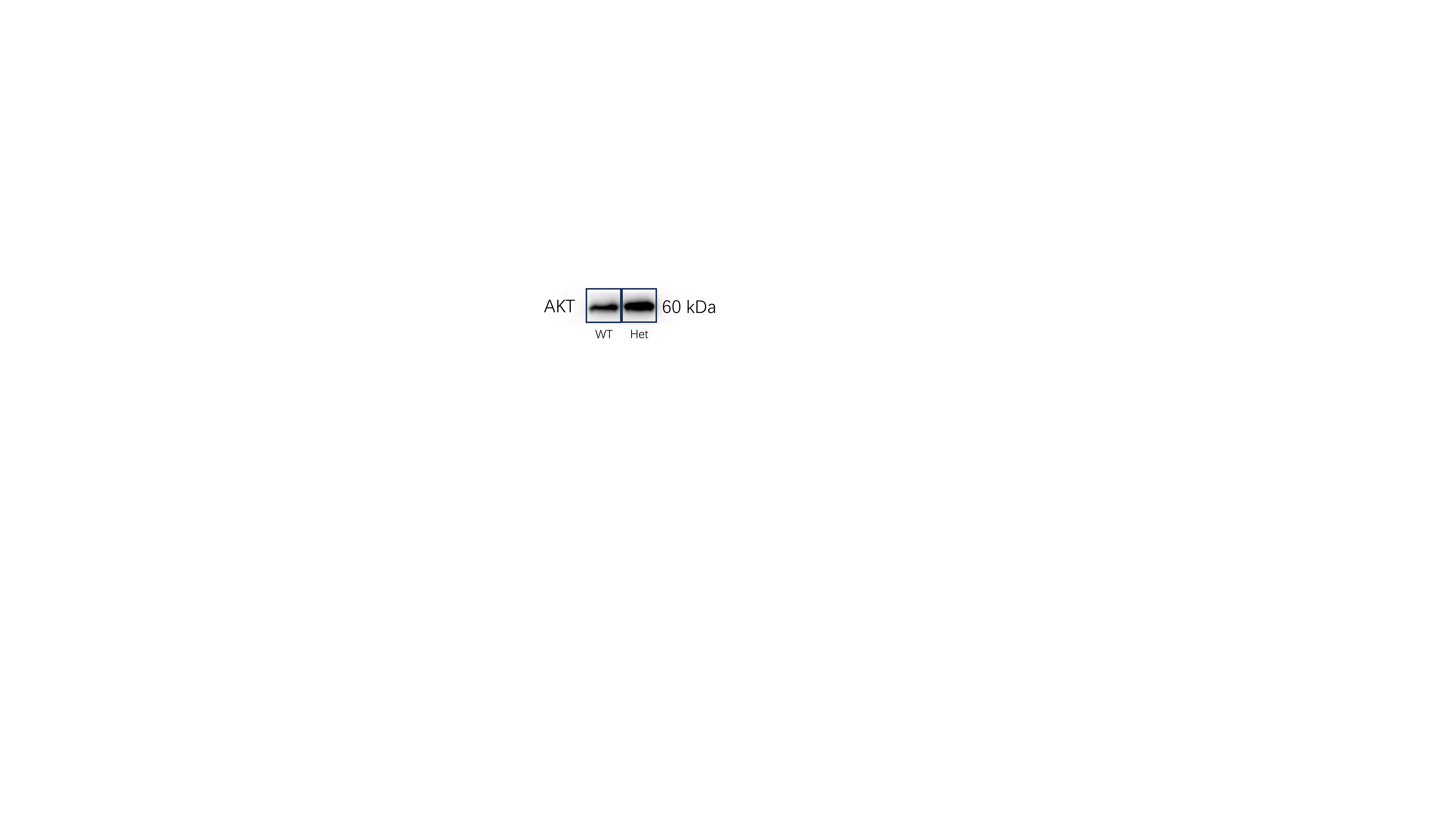

Supplement: Figure 6—source data 6. [file elife-91289-fig6-data6.zip › Figure 6-source data 6/AKT-2.TIF]

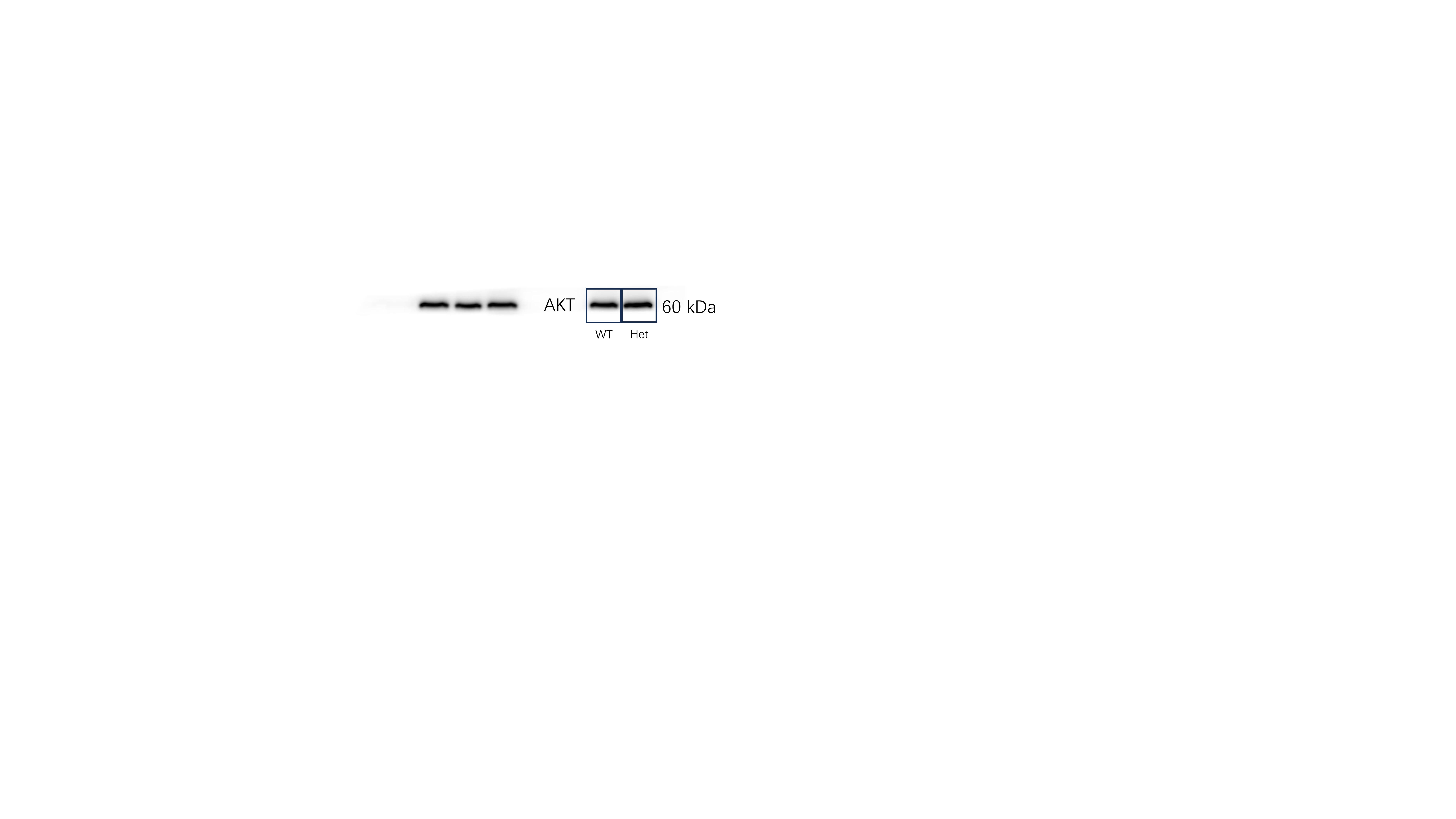

Supplement: Figure 6—source data 6. [file elife-91289-fig6-data6.zip › Figure 6-source data 6/AKT-3.TIF]

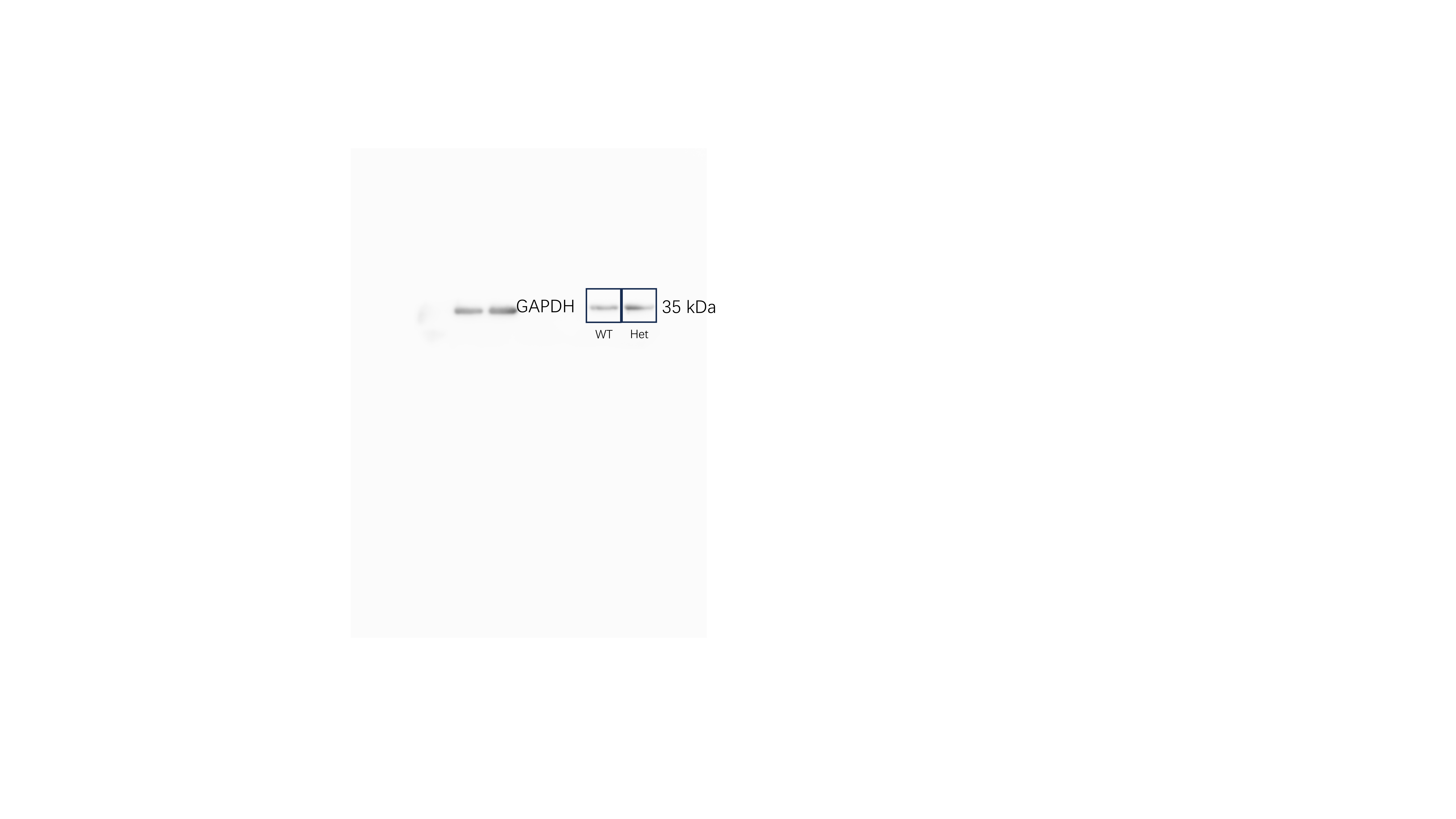

Supplement: Figure 6—source data 6. [file elife-91289-fig6-data6.zip › Figure 6-source data 6/GAPDH-1.TIF]

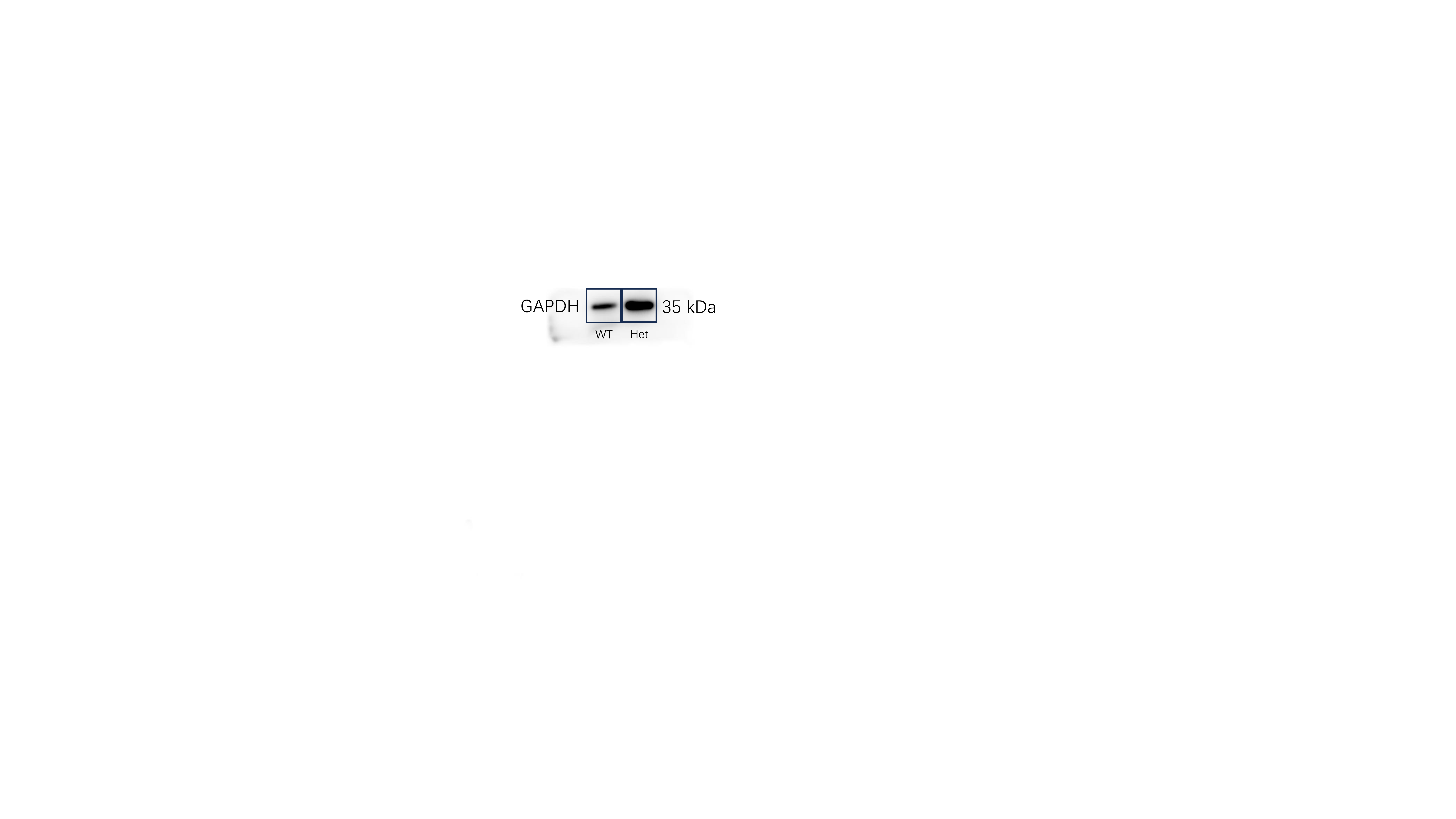

Supplement: Figure 6—source data 6. [file elife-91289-fig6-data6.zip › Figure 6-source data 6/GAPDH-2.TIF]

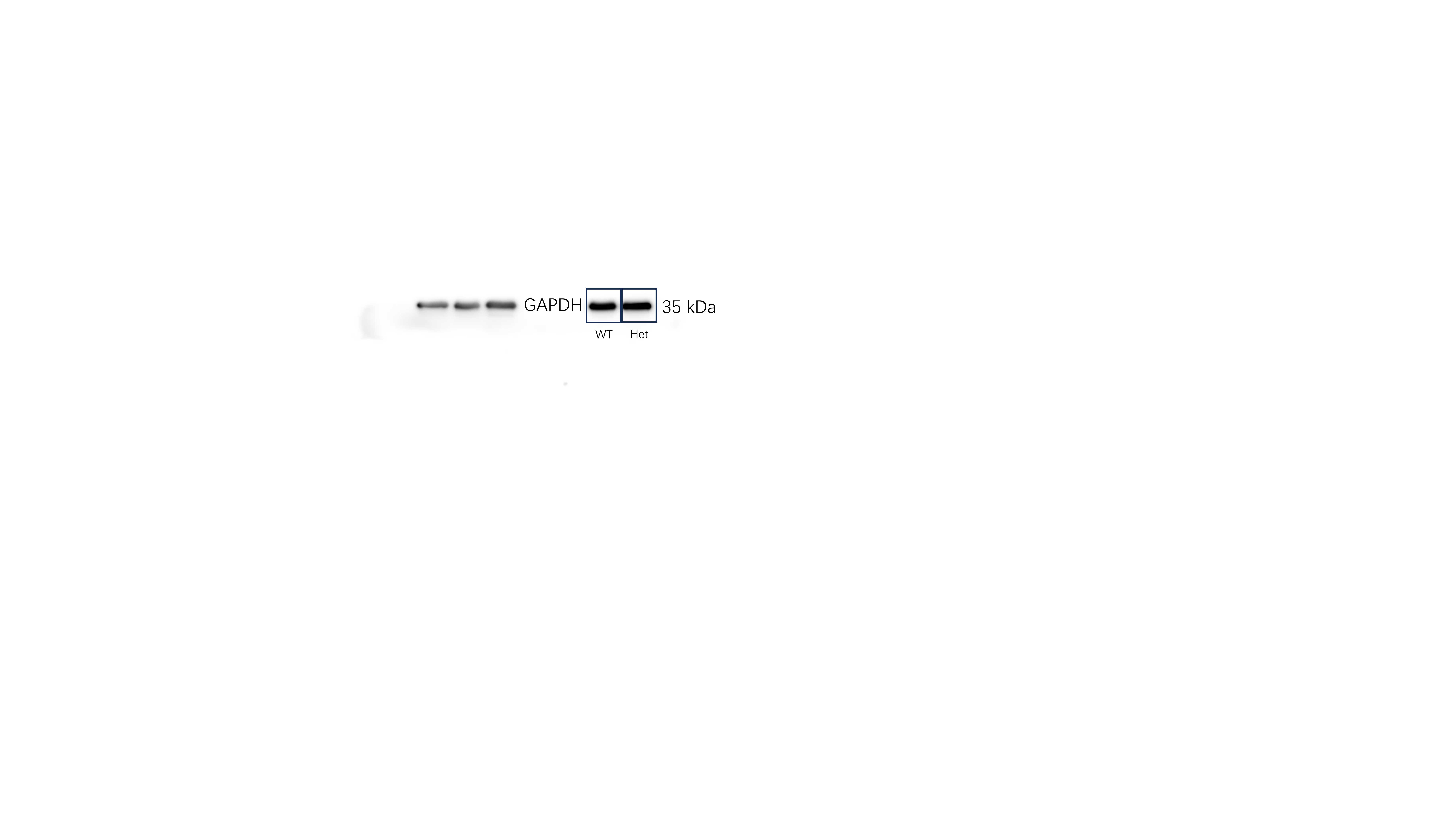

Supplement: Figure 6—source data 6. [file elife-91289-fig6-data6.zip › Figure 6-source data 6/GAPDH-3.TIF]

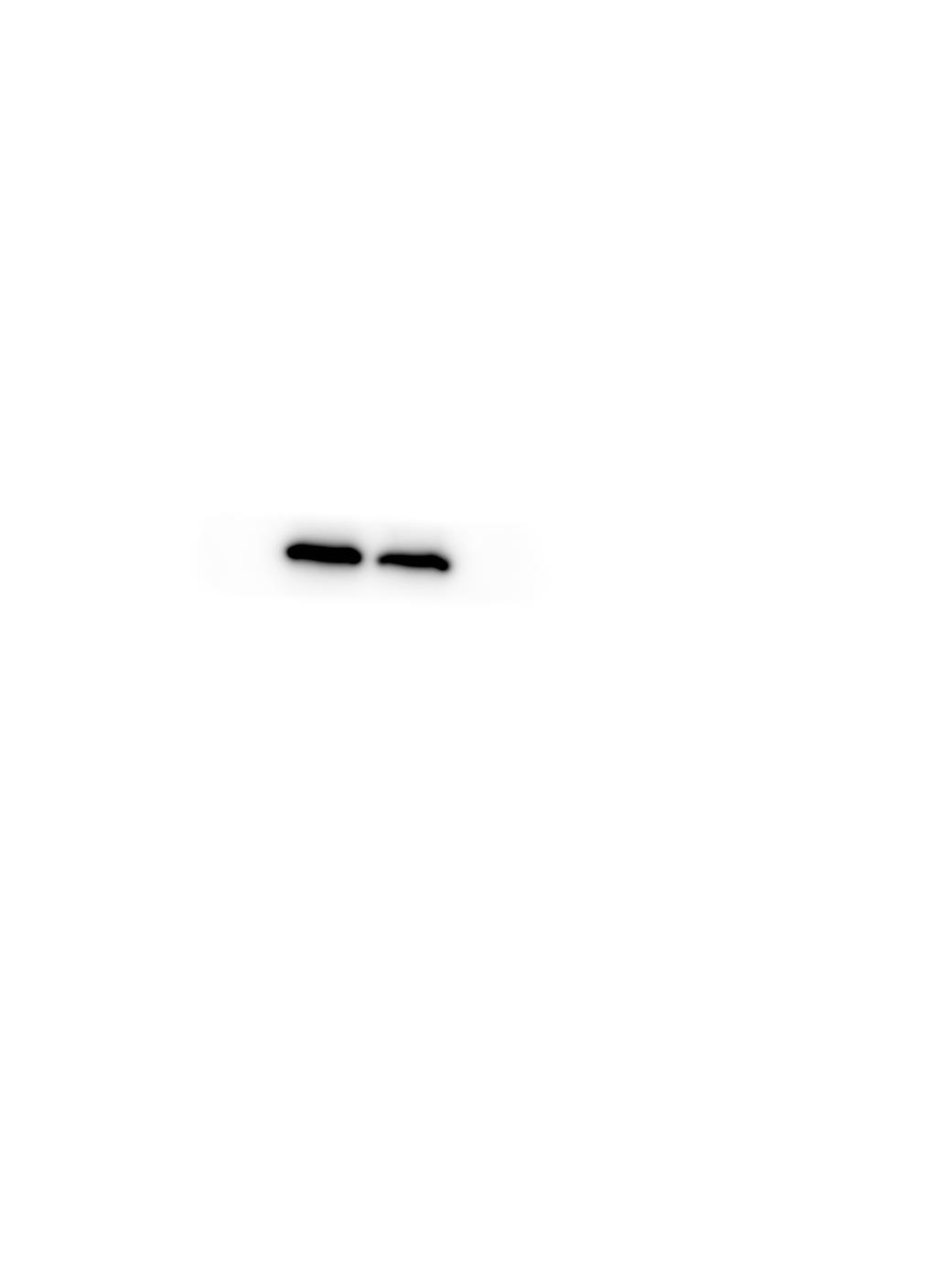

Supplement: Figure 6—source data 7. [file elife-91289-fig6-data7.zip › Figure 6-source data 7/p-AKT-1.jpg]

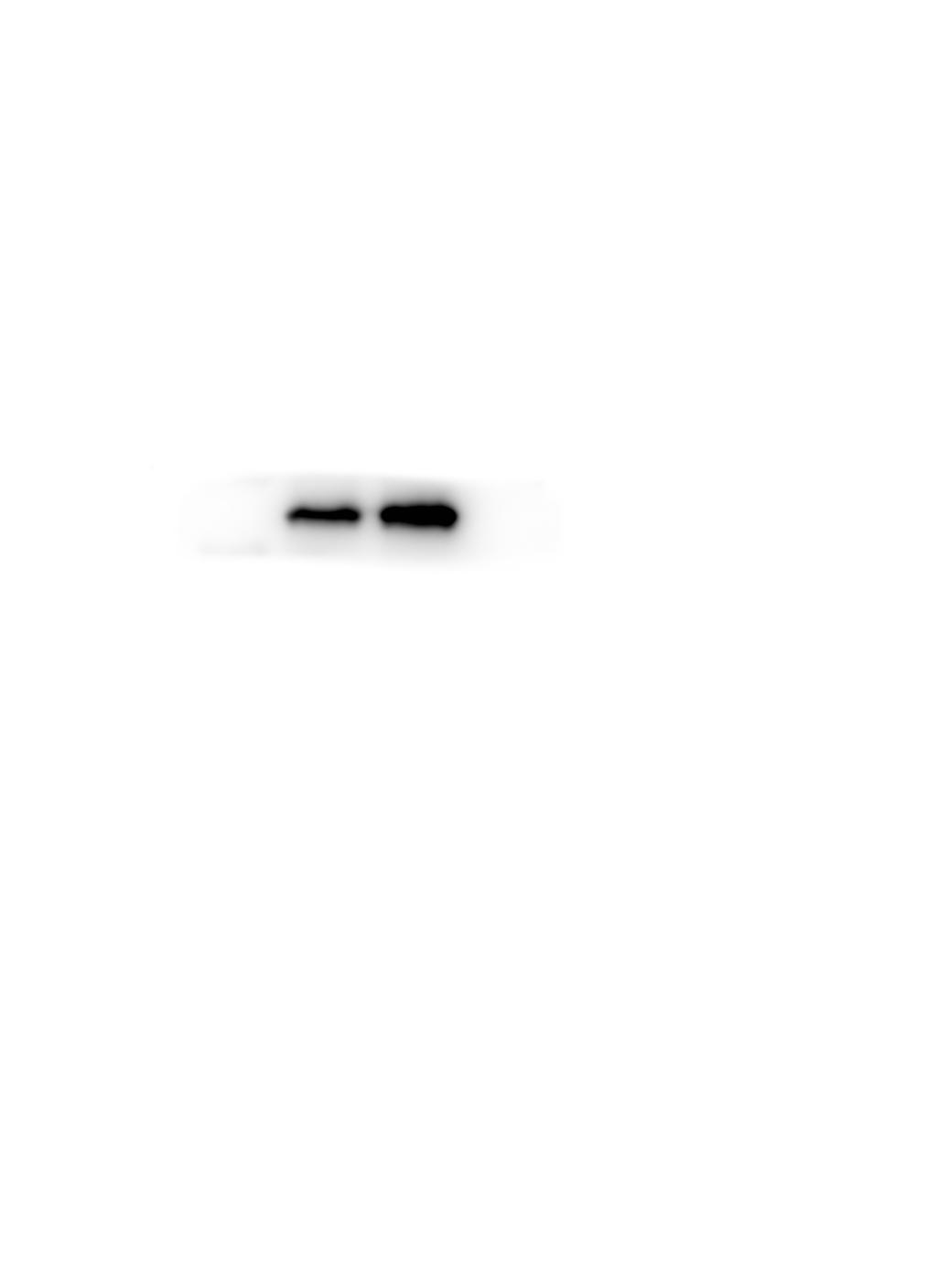

Supplement: Figure 6—source data 7. [file elife-91289-fig6-data7.zip › Figure 6-source data 7/p-AKT-2.jpg]

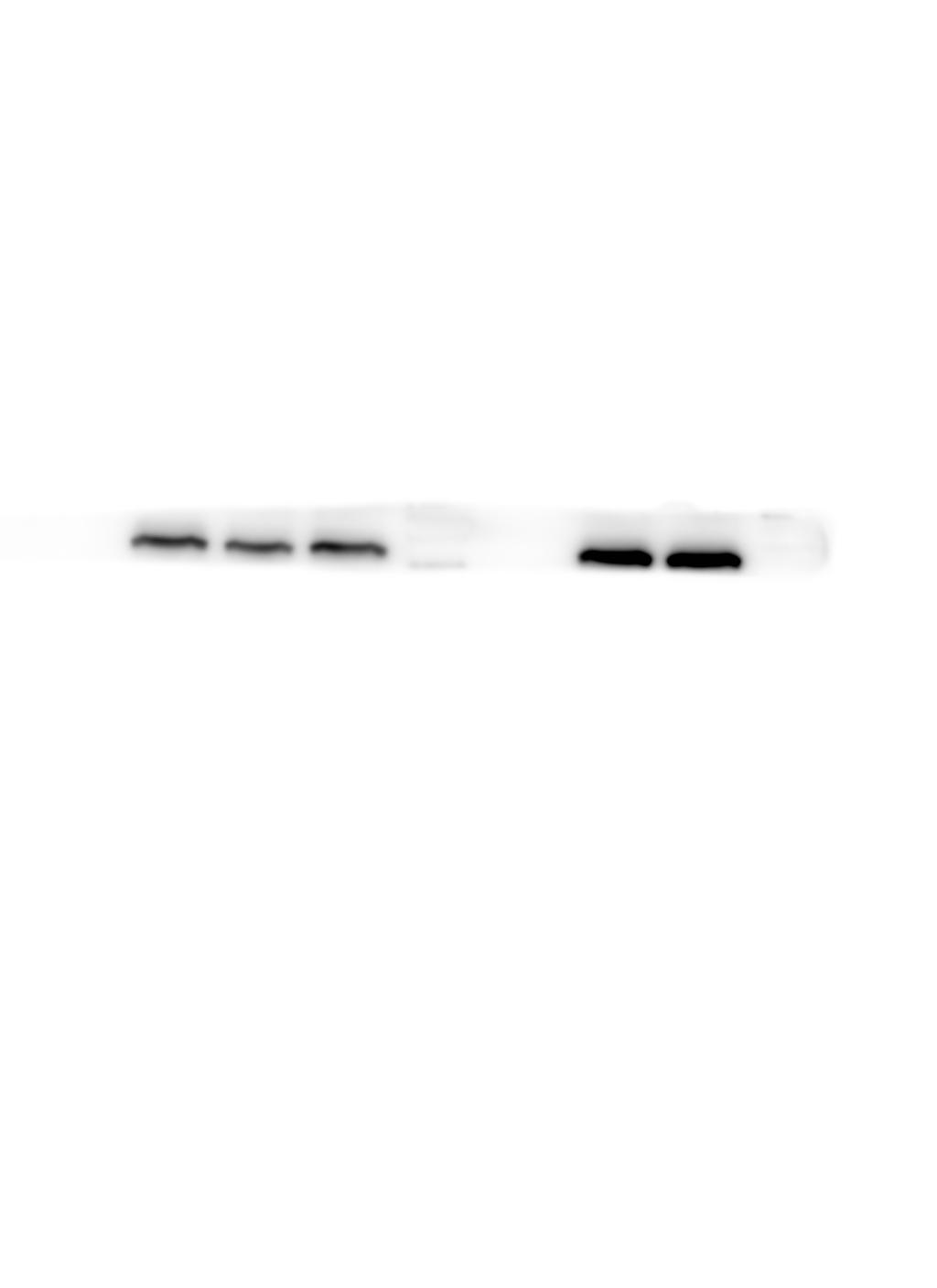

Supplement: Figure 6—source data 7. [file elife-91289-fig6-data7.zip › Figure 6-source data 7/p-AKT-3.jpg]

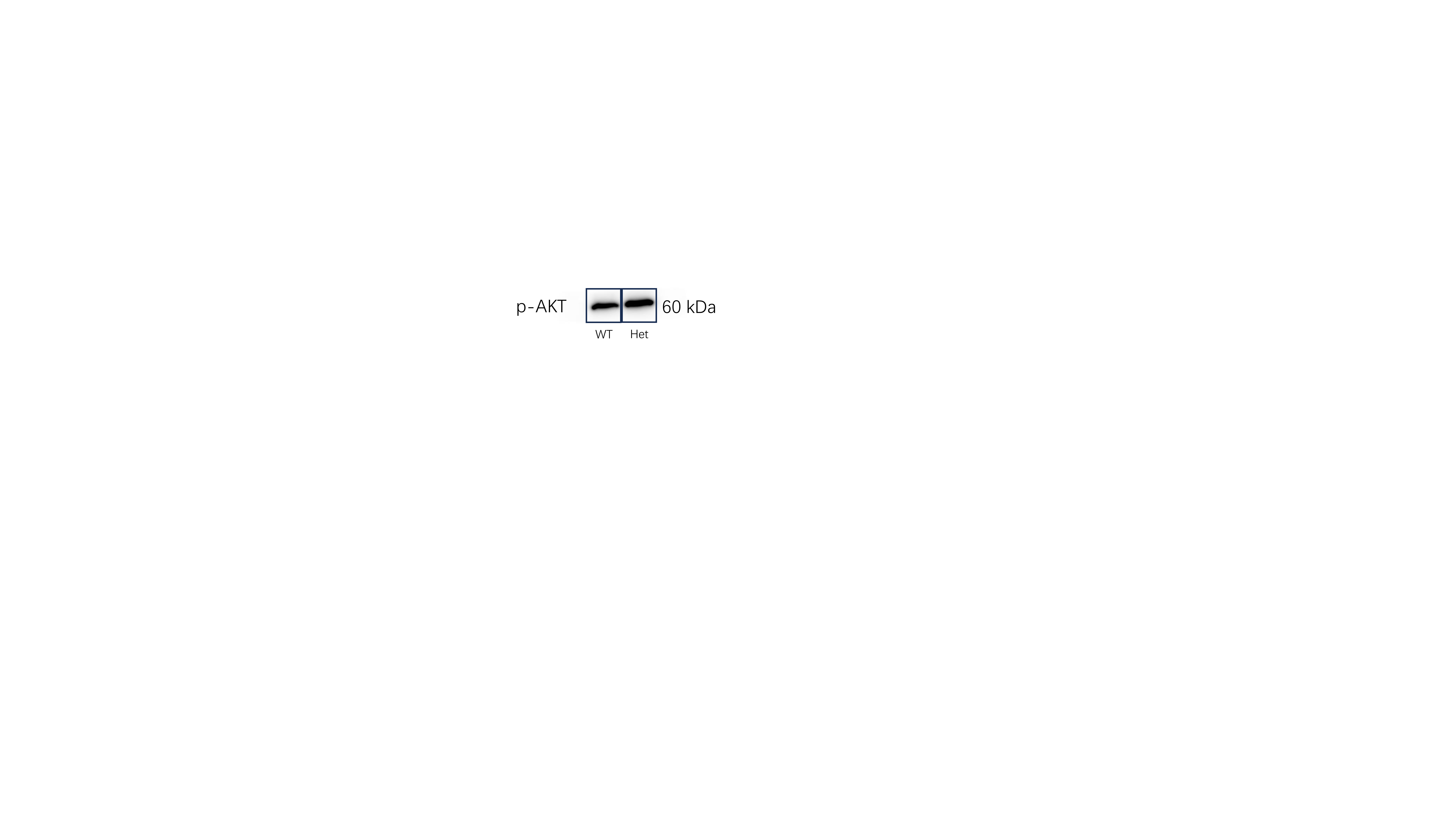

Supplement: Figure 6—source data 8. [file elife-91289-fig6-data8.zip › Figure 6-source data 8/p-AKT-1.TIF]

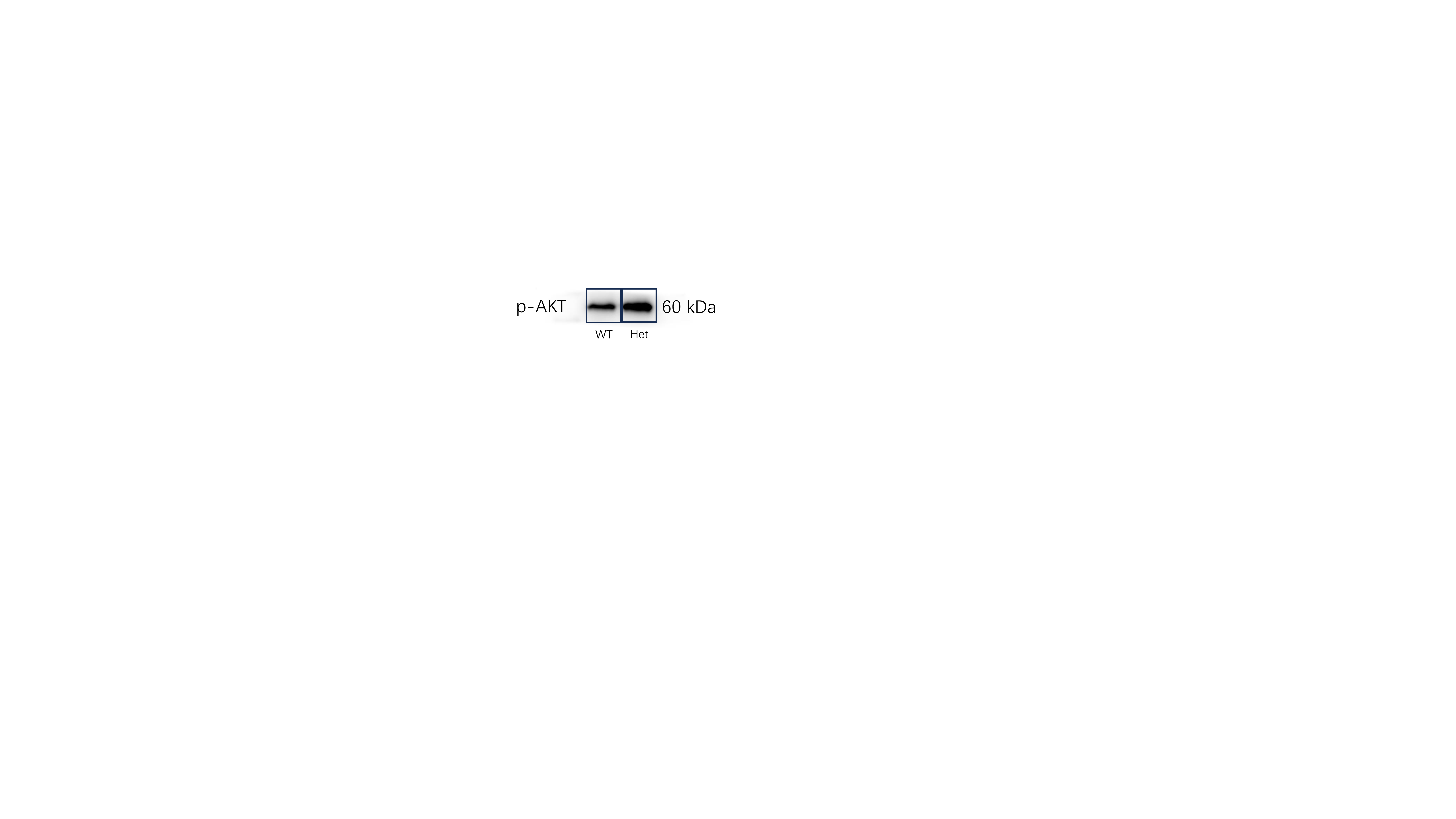

Supplement: Figure 6—source data 8. [file elife-91289-fig6-data8.zip › Figure 6-source data 8/p-AKT-2.TIF]

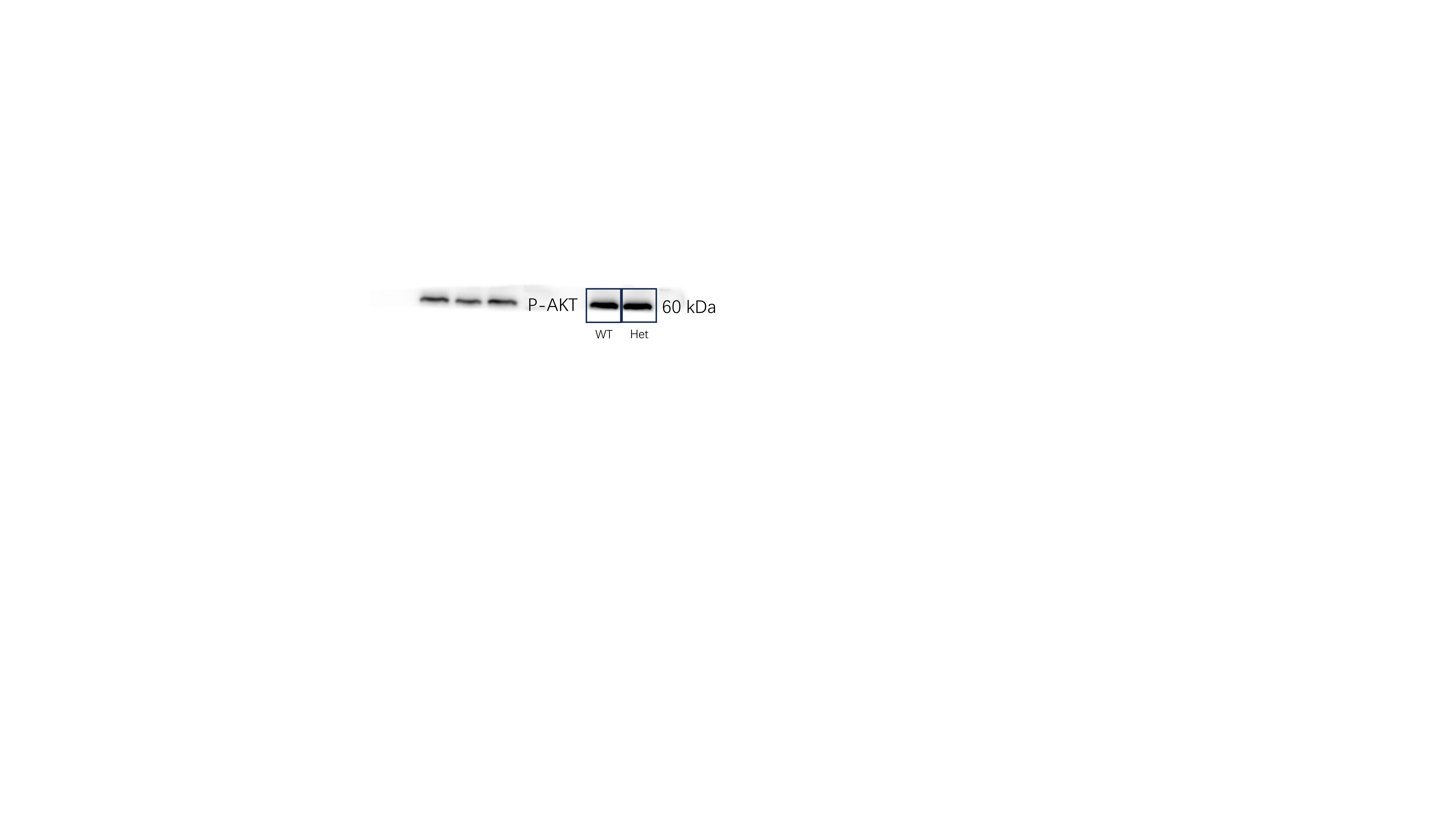

Supplement: Figure 6—source data 8. [file elife-91289-fig6-data8.zip › Figure 6-source data 8/p-AKT-3.TIF]

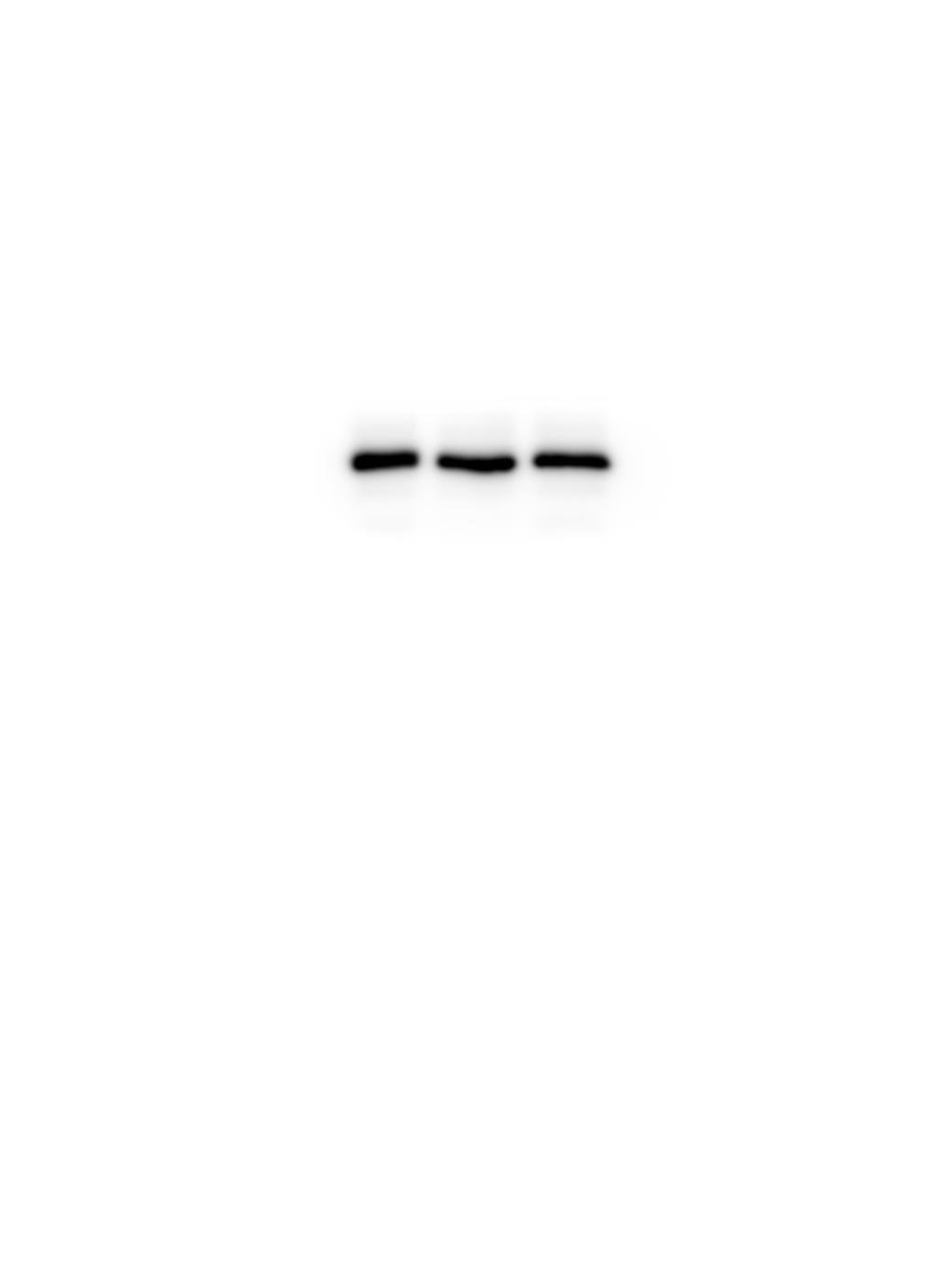

Supplement: Figure 6—source data 9. [file elife-91289-fig6-data9.zip › Figure 6-source data 9/GAPDH-1.jpg]

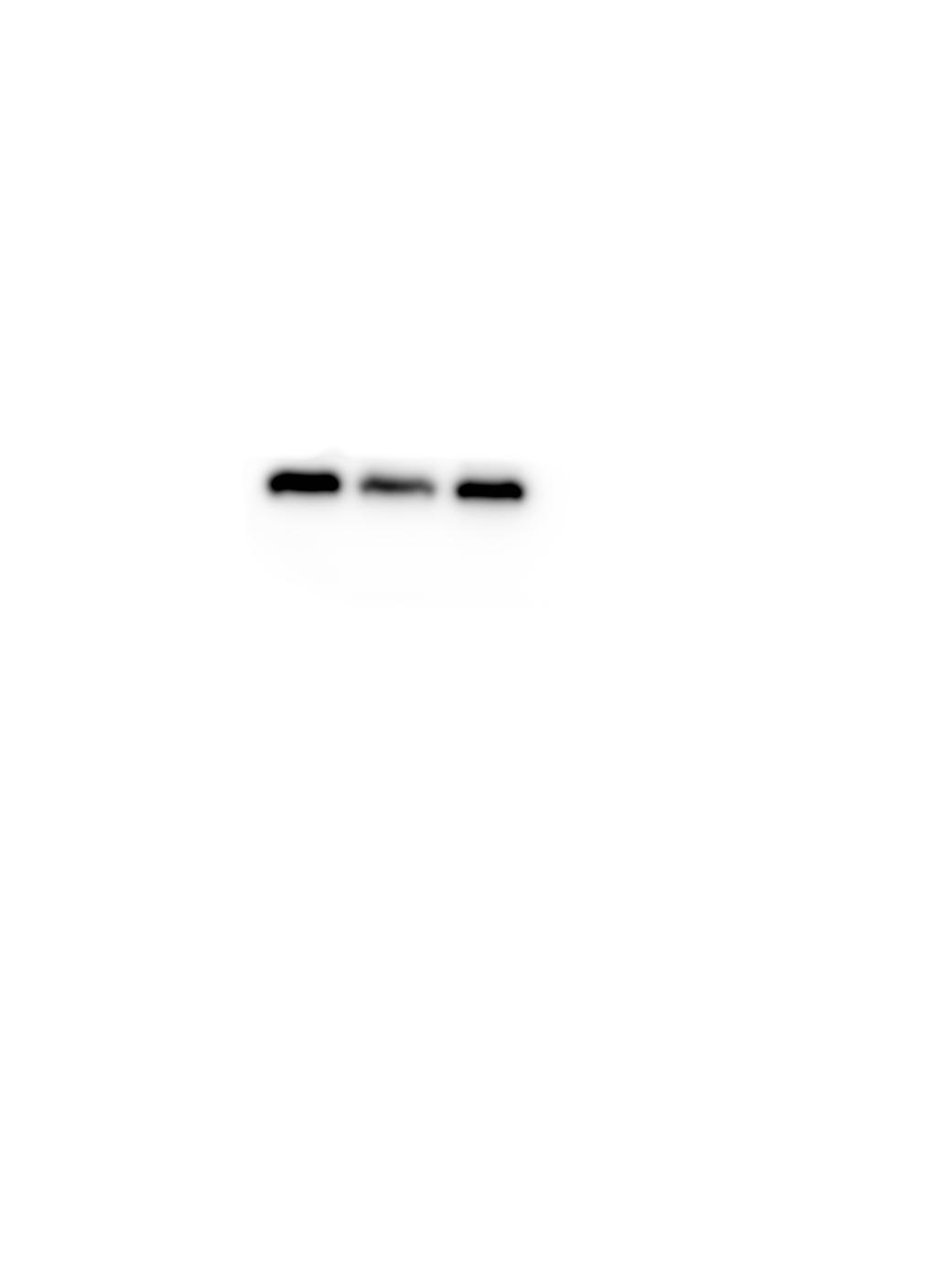

Supplement: Figure 6—source data 9. [file elife-91289-fig6-data9.zip › Figure 6-source data 9/IκBα-1.jpg]

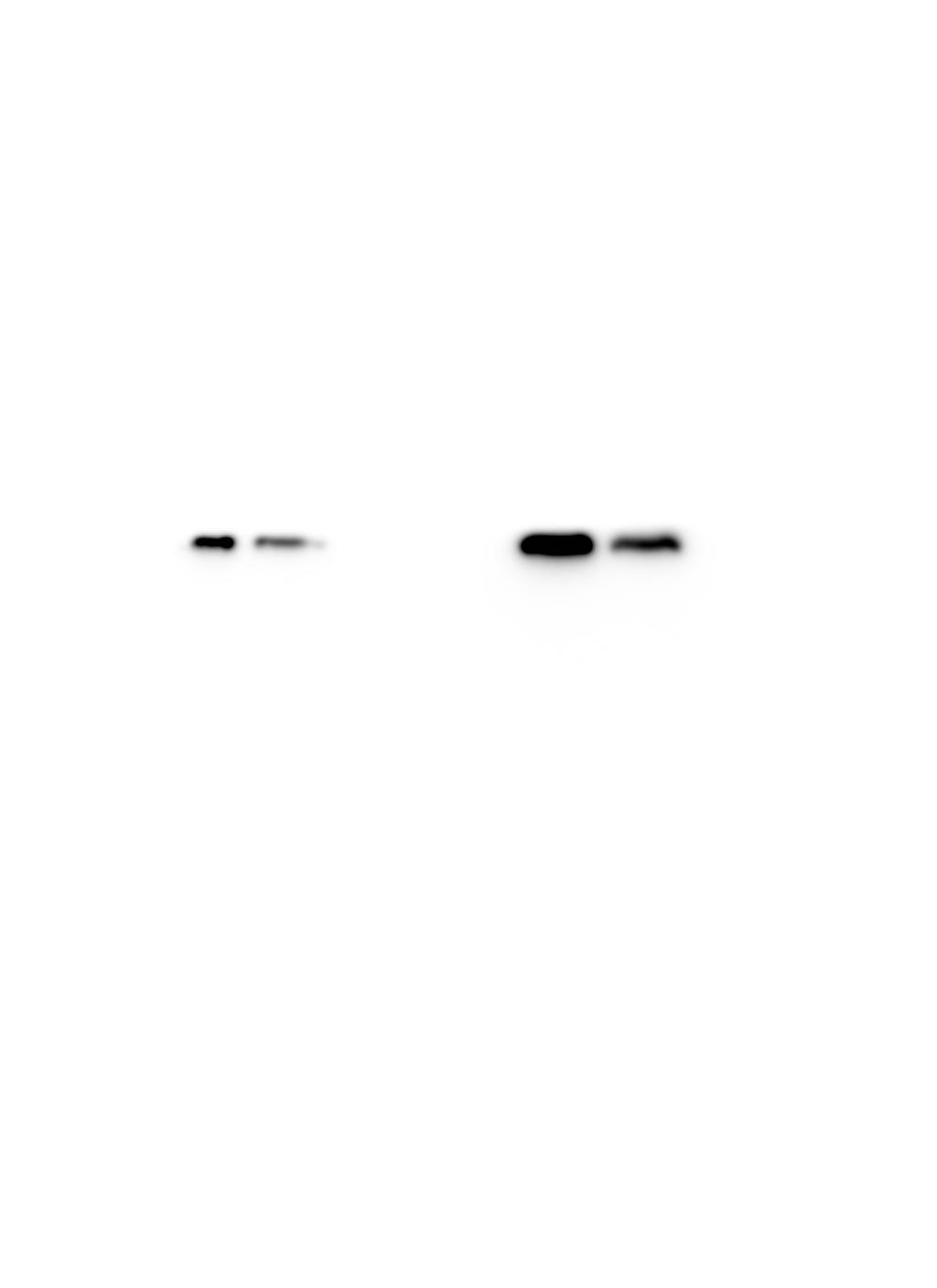

Supplement: Figure 6—source data 9. [file elife-91289-fig6-data9.zip › Figure 6-source data 9/IκBα-2.jpg]

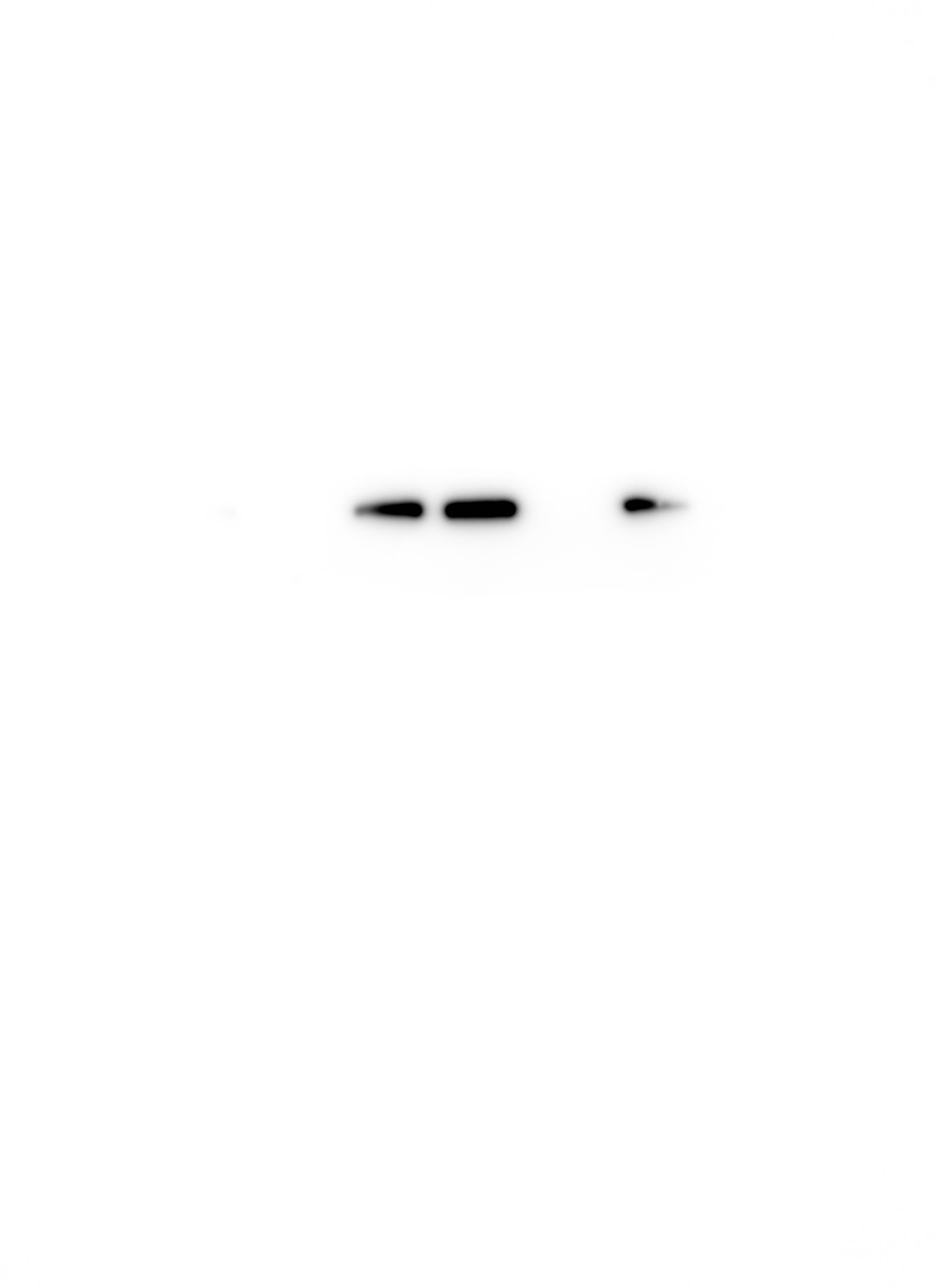

Supplement: Figure 6—source data 9. [file elife-91289-fig6-data9.zip › Figure 6-source data 9/IκBα-3.jpg]

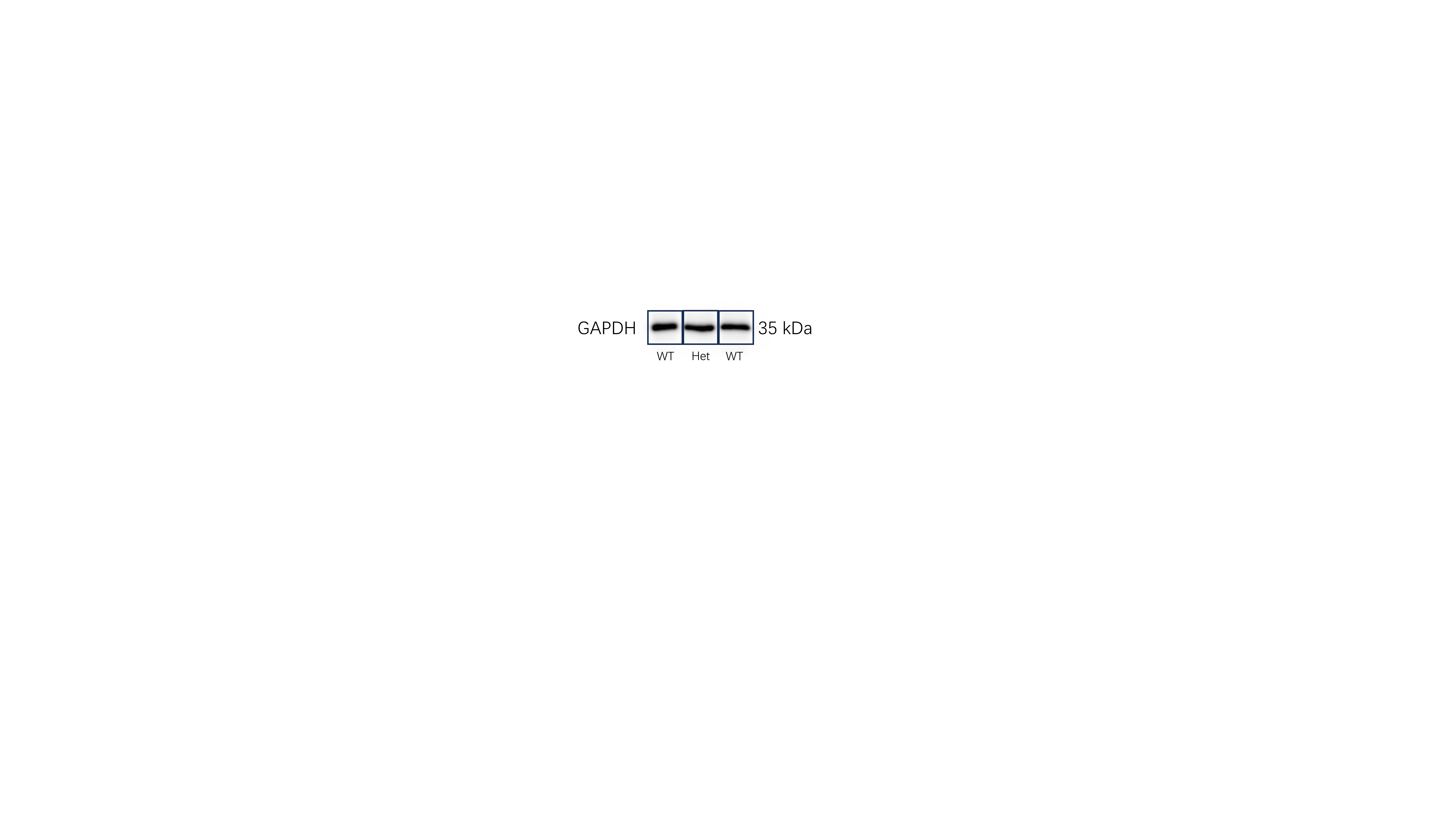

Supplement: Figure 6—source data 10. [file elife-91289-fig6-data10.zip › Figure 6-source data 10/GAPDH-1.TIF]

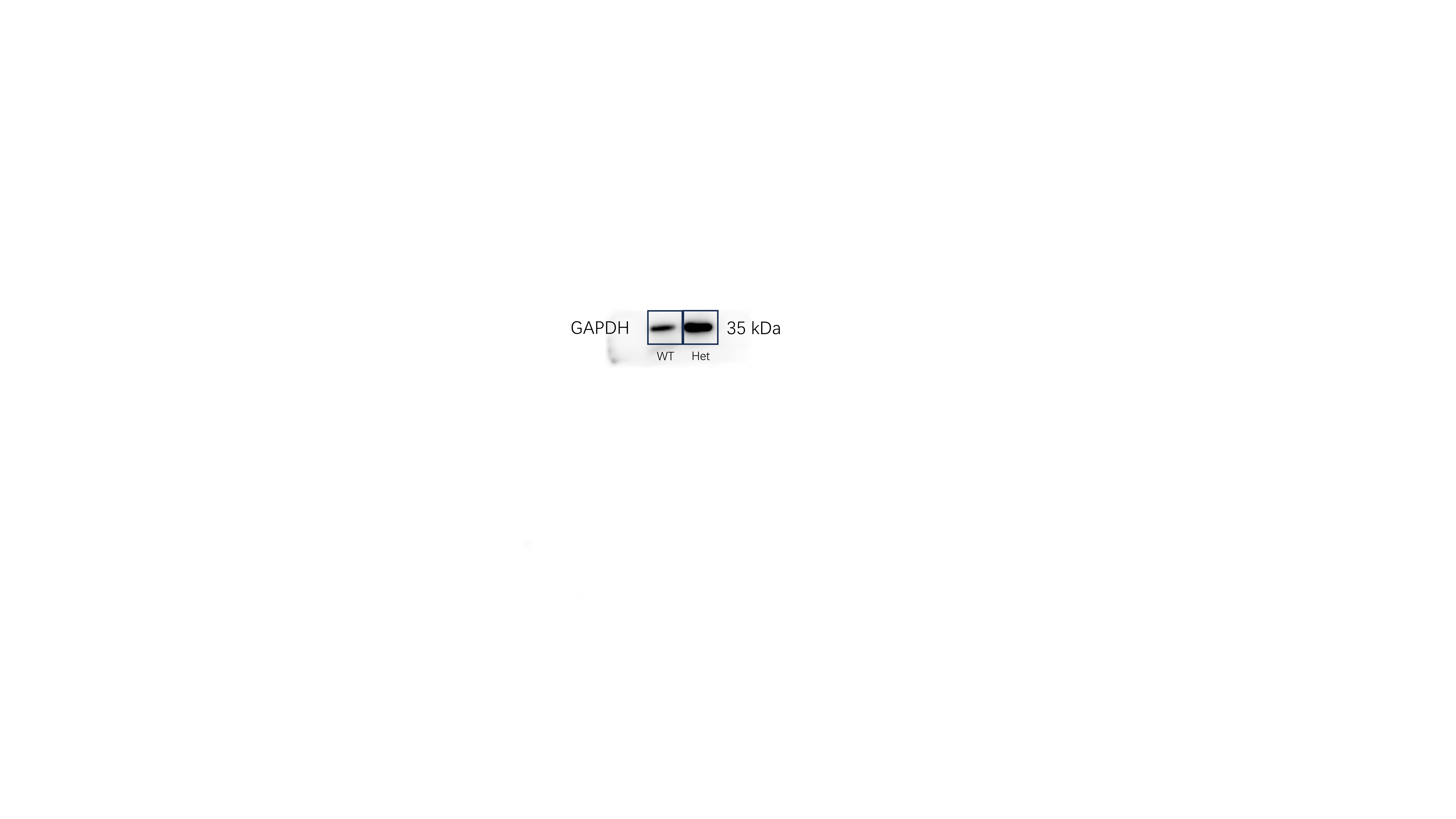

Supplement: Figure 6—source data 10. [file elife-91289-fig6-data10.zip › Figure 6-source data 10/GAPDH-2.TIF]

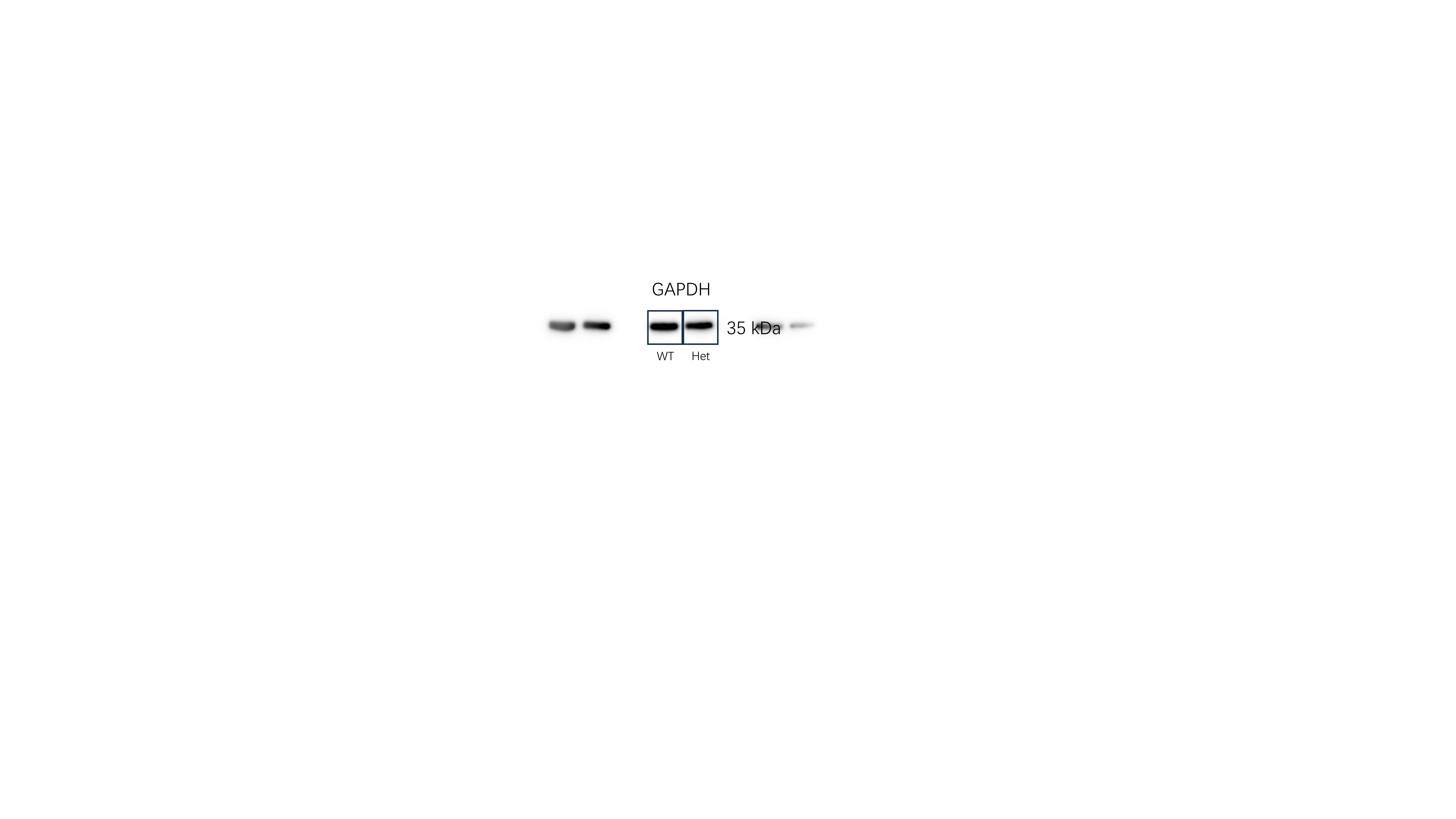

Supplement: Figure 6—source data 10. [file elife-91289-fig6-data10.zip › Figure 6-source data 10/GAPDH-3.TIF]

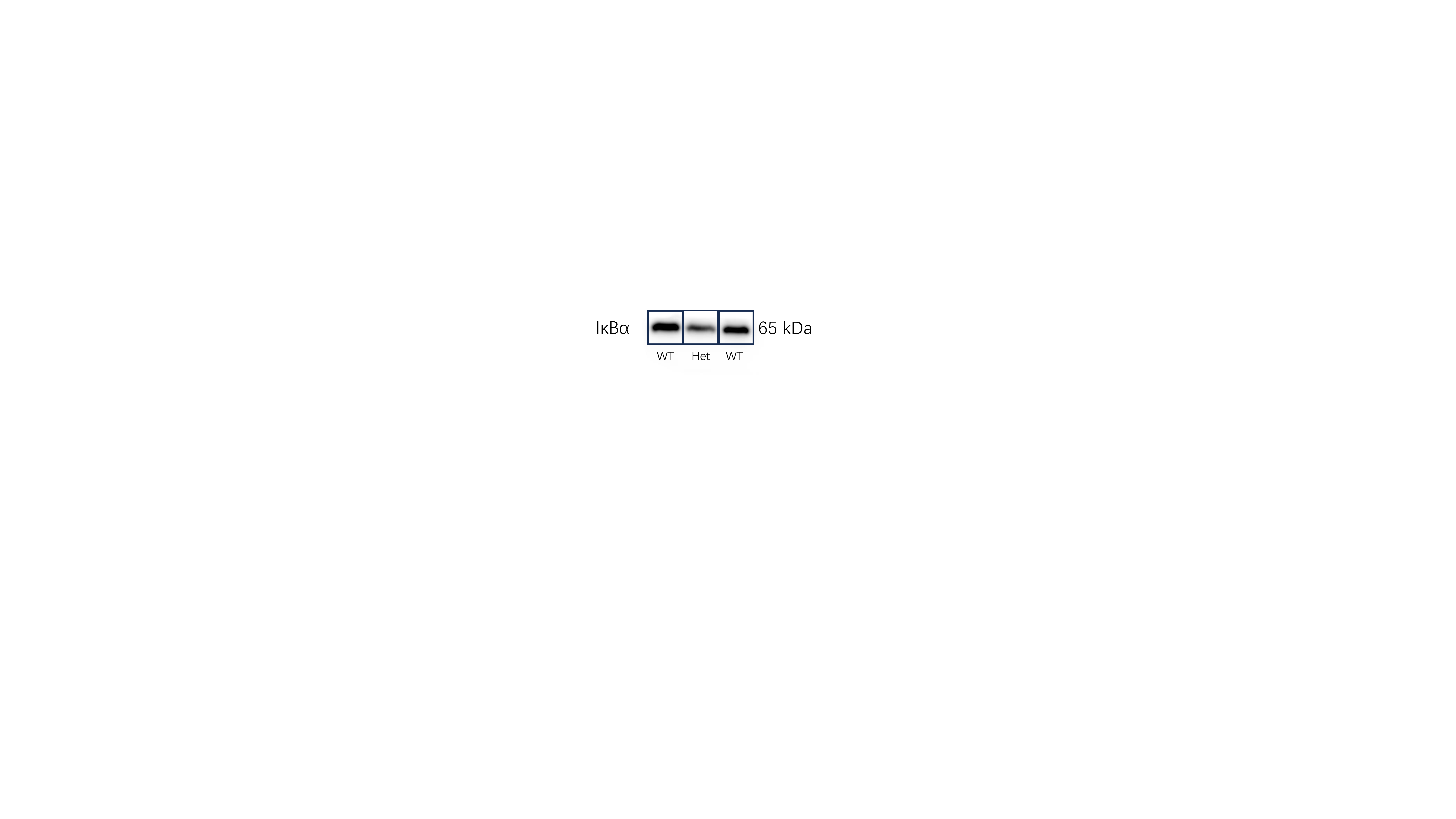

Supplement: Figure 6—source data 10. [file elife-91289-fig6-data10.zip › Figure 6-source data 10/IκBα-1.TIF]

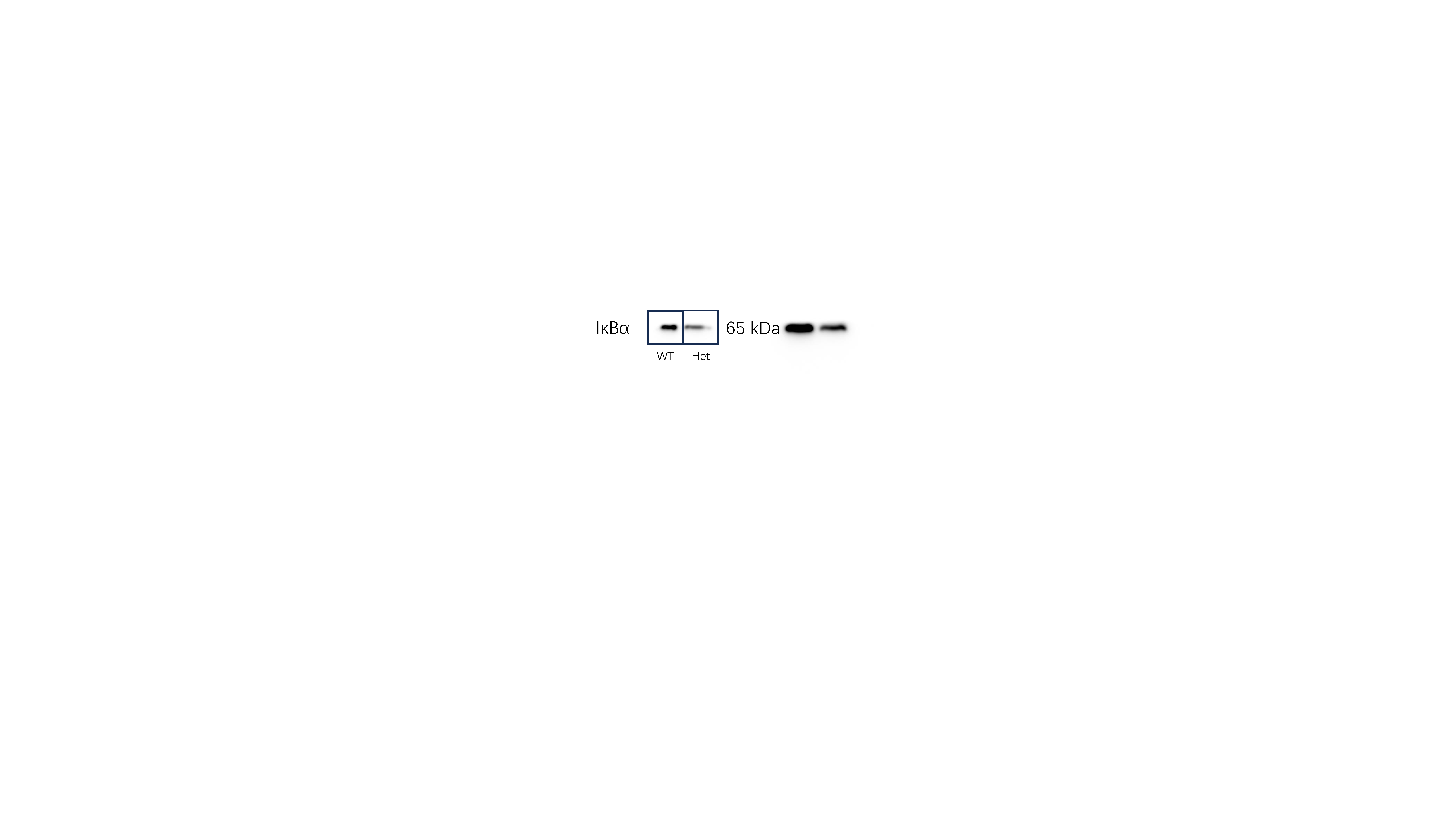

Supplement: Figure 6—source data 10. [file elife-91289-fig6-data10.zip › Figure 6-source data 10/IκBα-2.TIF]

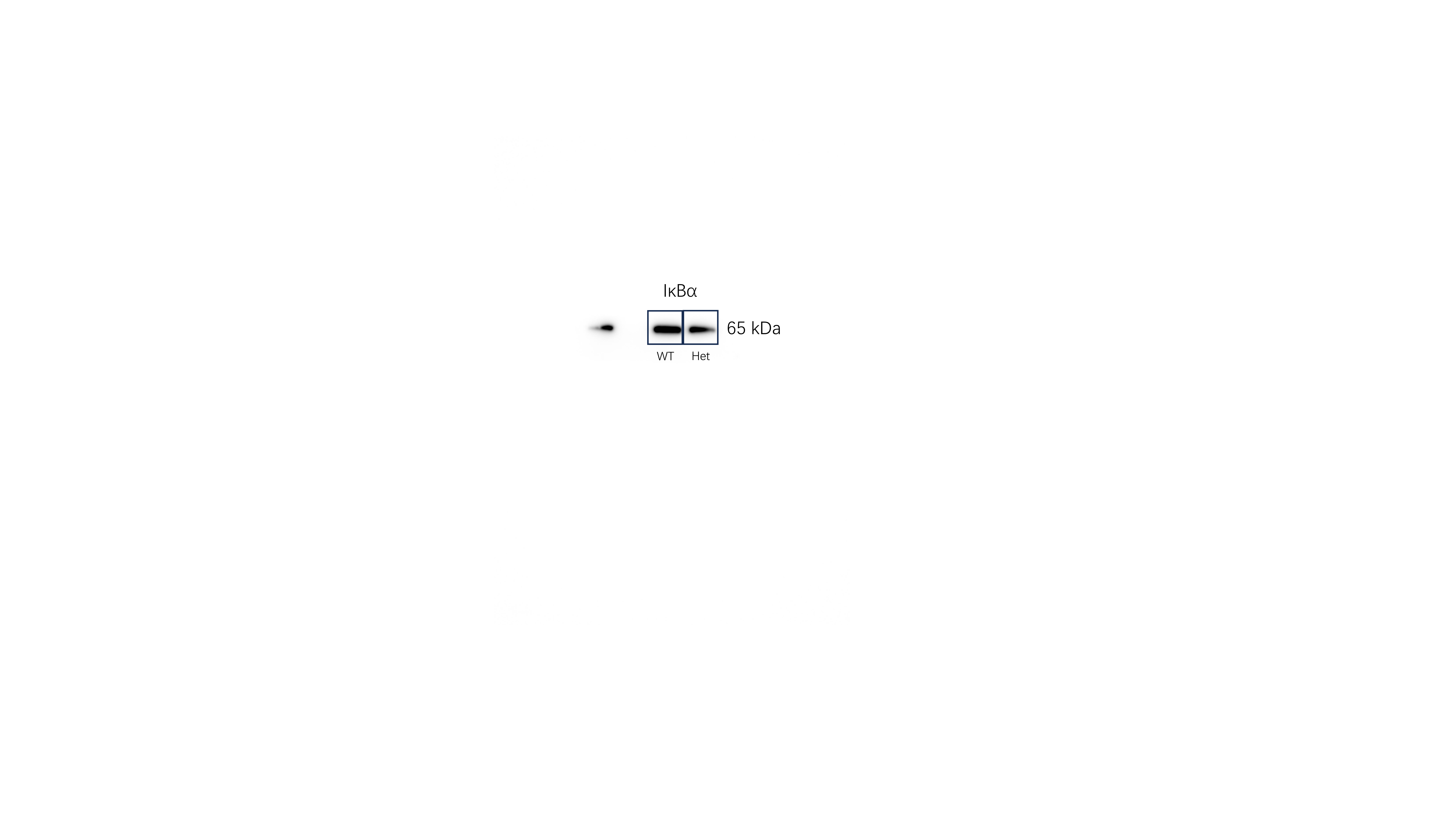

Supplement: Figure 6—source data 10. [file elife-91289-fig6-data10.zip › Figure 6-source data 10/IκBα-3.TIF]

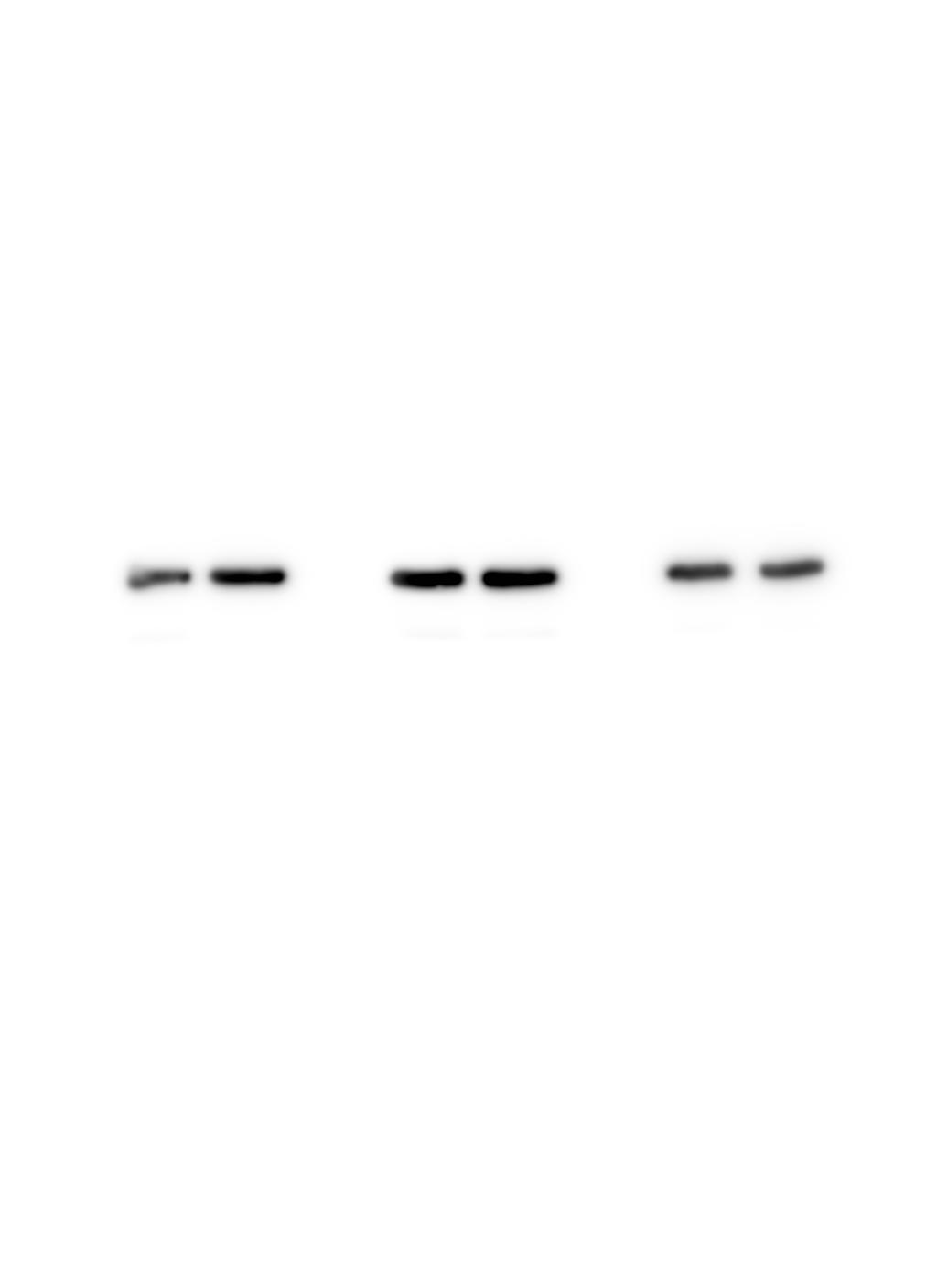

Supplement: Figure 6—source data 11. [file elife-91289-fig6-data11.zip › Figure 6-source data 11/GAPDH-1.jpg]

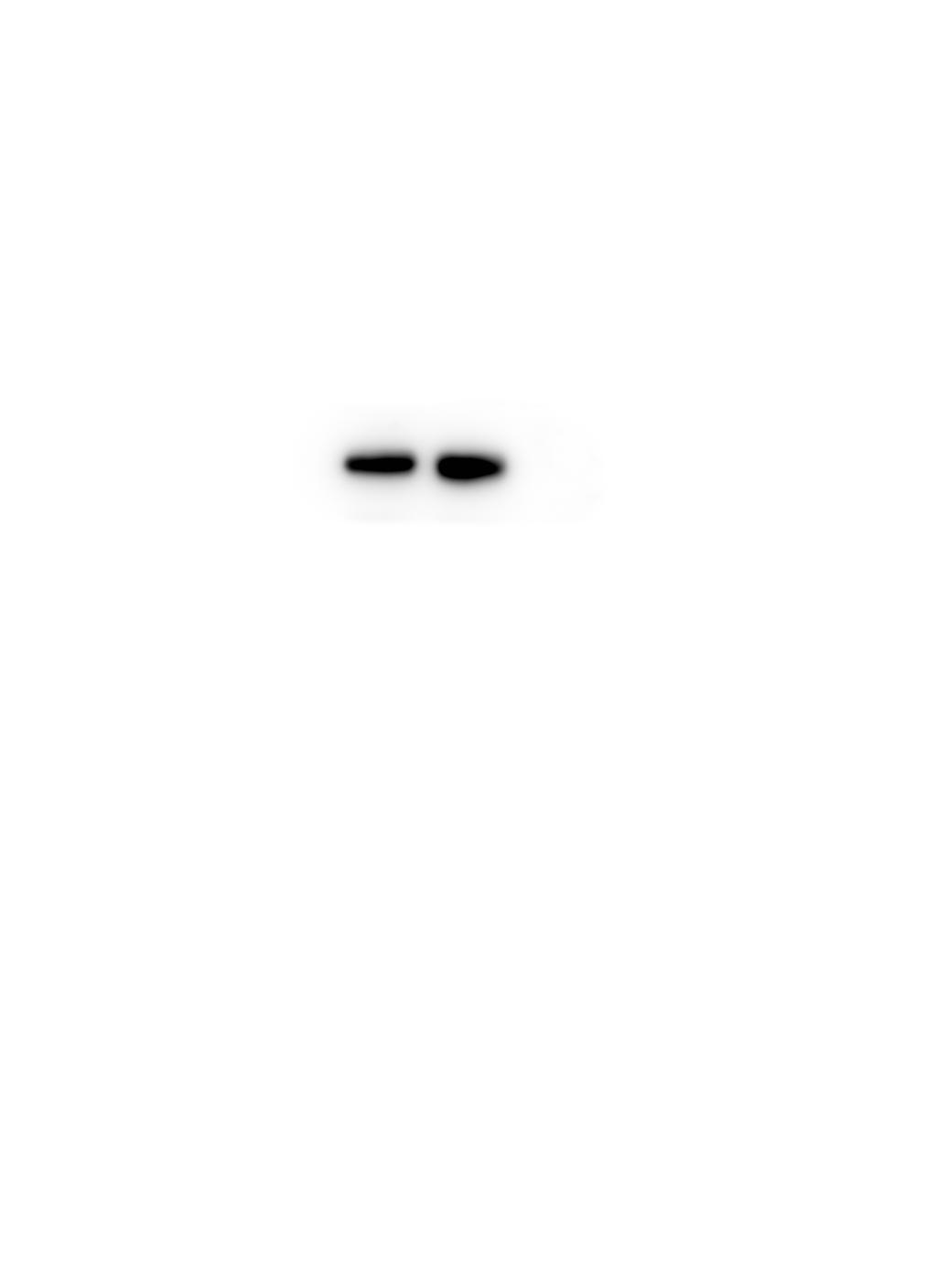

Supplement: Figure 6—source data 11. [file elife-91289-fig6-data11.zip › Figure 6-source data 11/GAPDH-2.jpg]

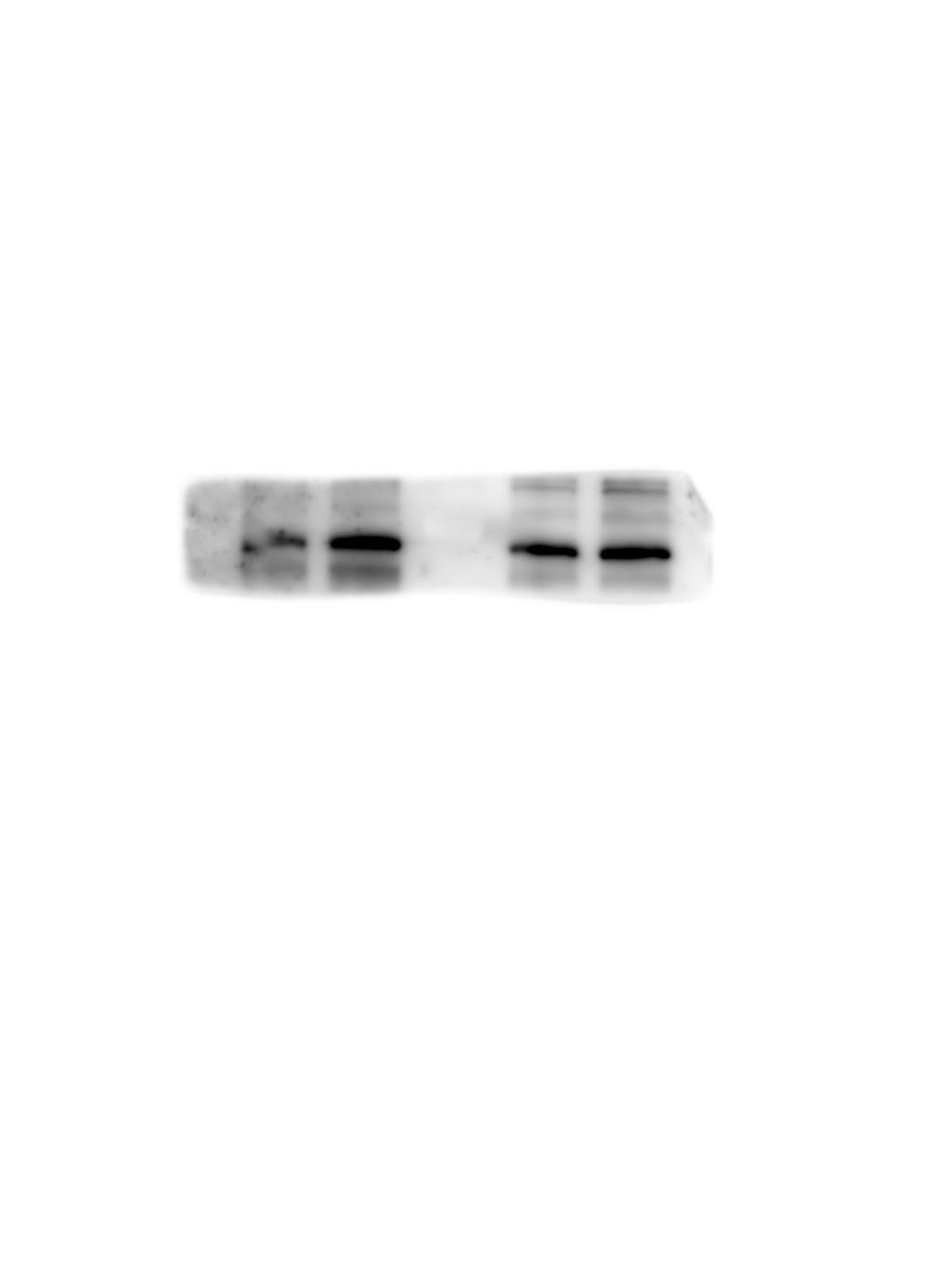

Supplement: Figure 6—source data 11. [file elife-91289-fig6-data11.zip › Figure 6-source data 11/NF-κB-1.jpg]

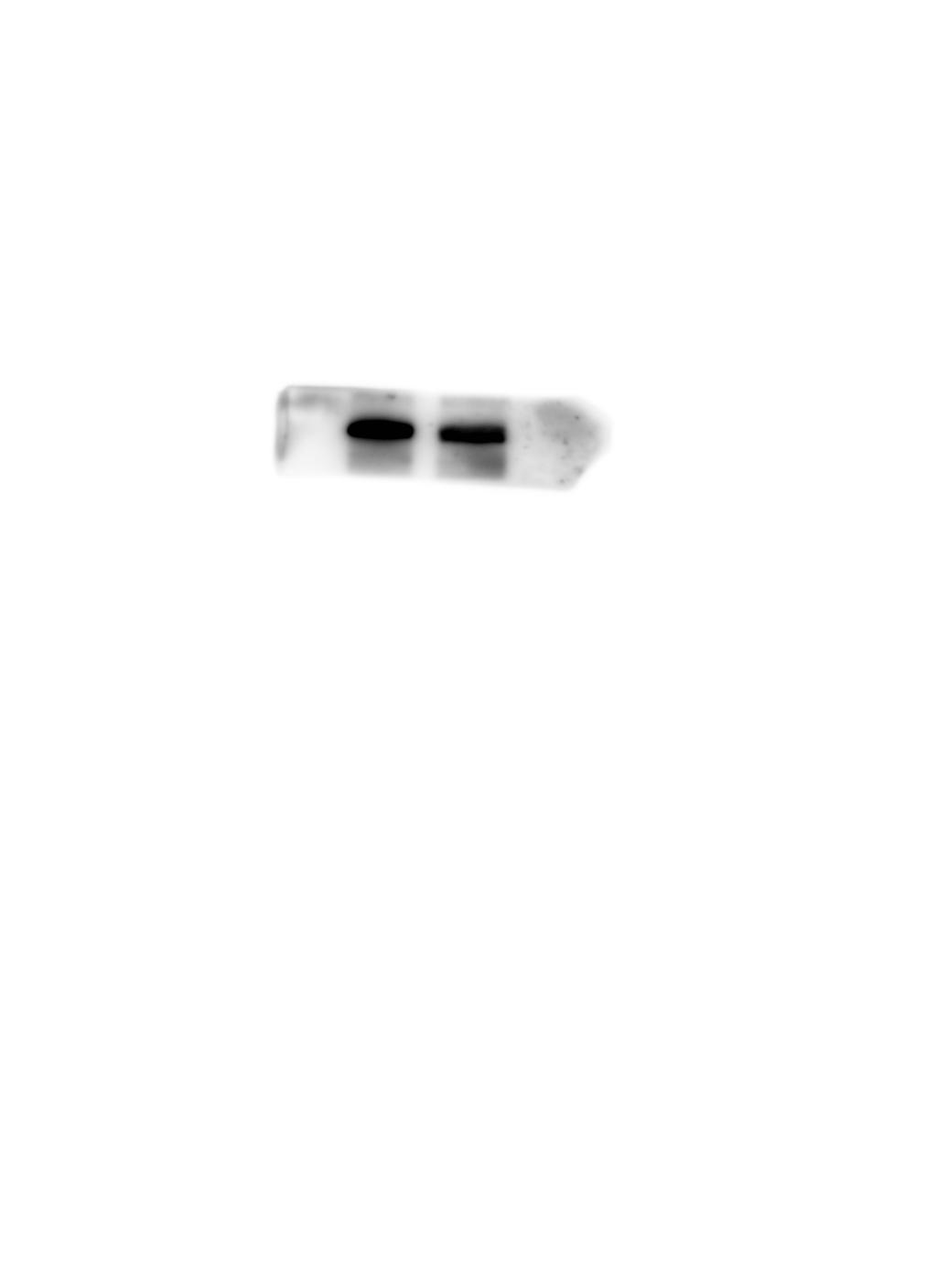

Supplement: Figure 6—source data 11. [file elife-91289-fig6-data11.zip › Figure 6-source data 11/NF-κB-2.jpg]

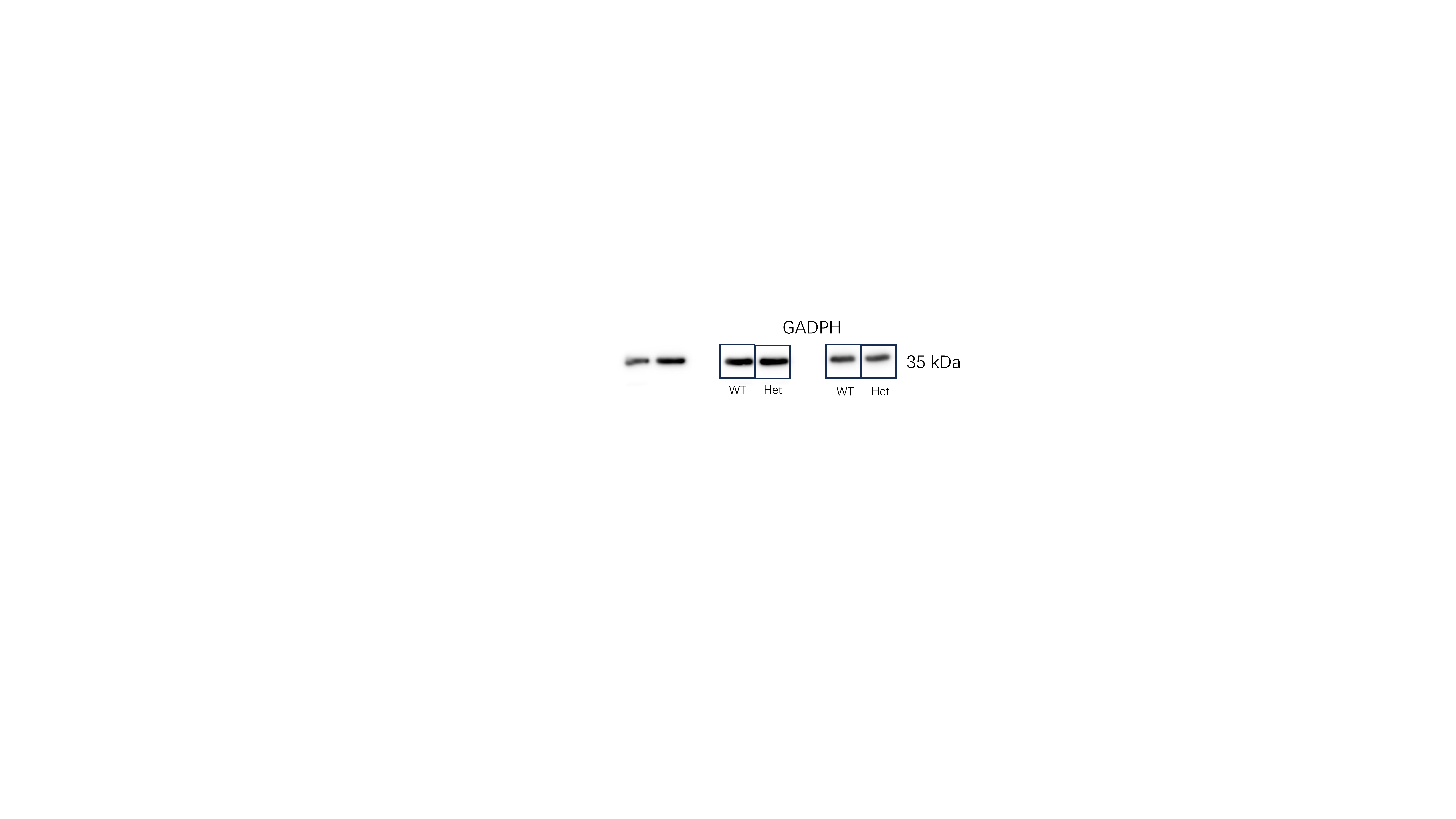

Supplement: Figure 6—source data 12. [file elife-91289-fig6-data12.zip › Figure 6-source data 12/GAPDH-1.TIF]

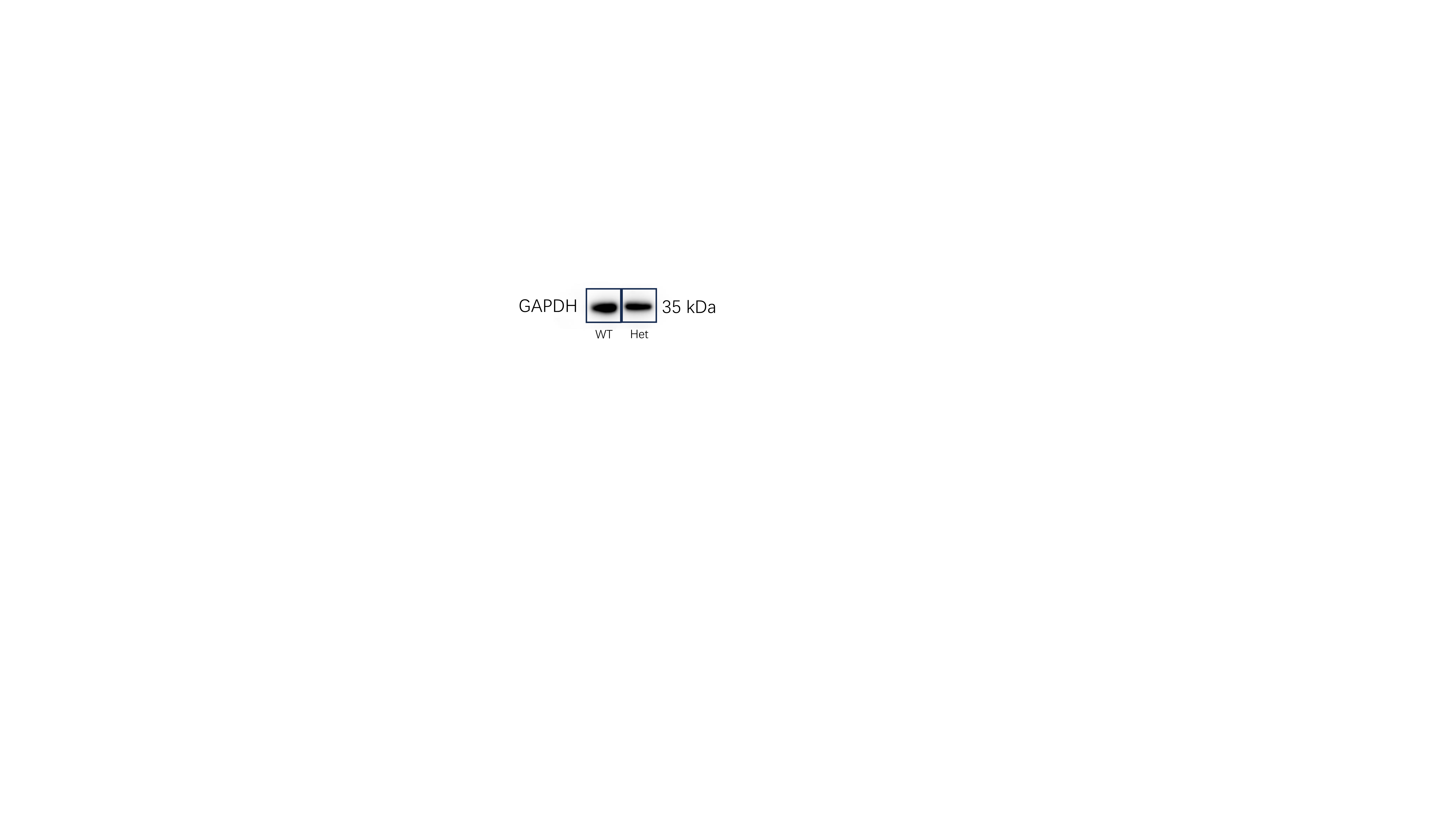

Supplement: Figure 6—source data 12. [file elife-91289-fig6-data12.zip › Figure 6-source data 12/GAPDH-2.tif]

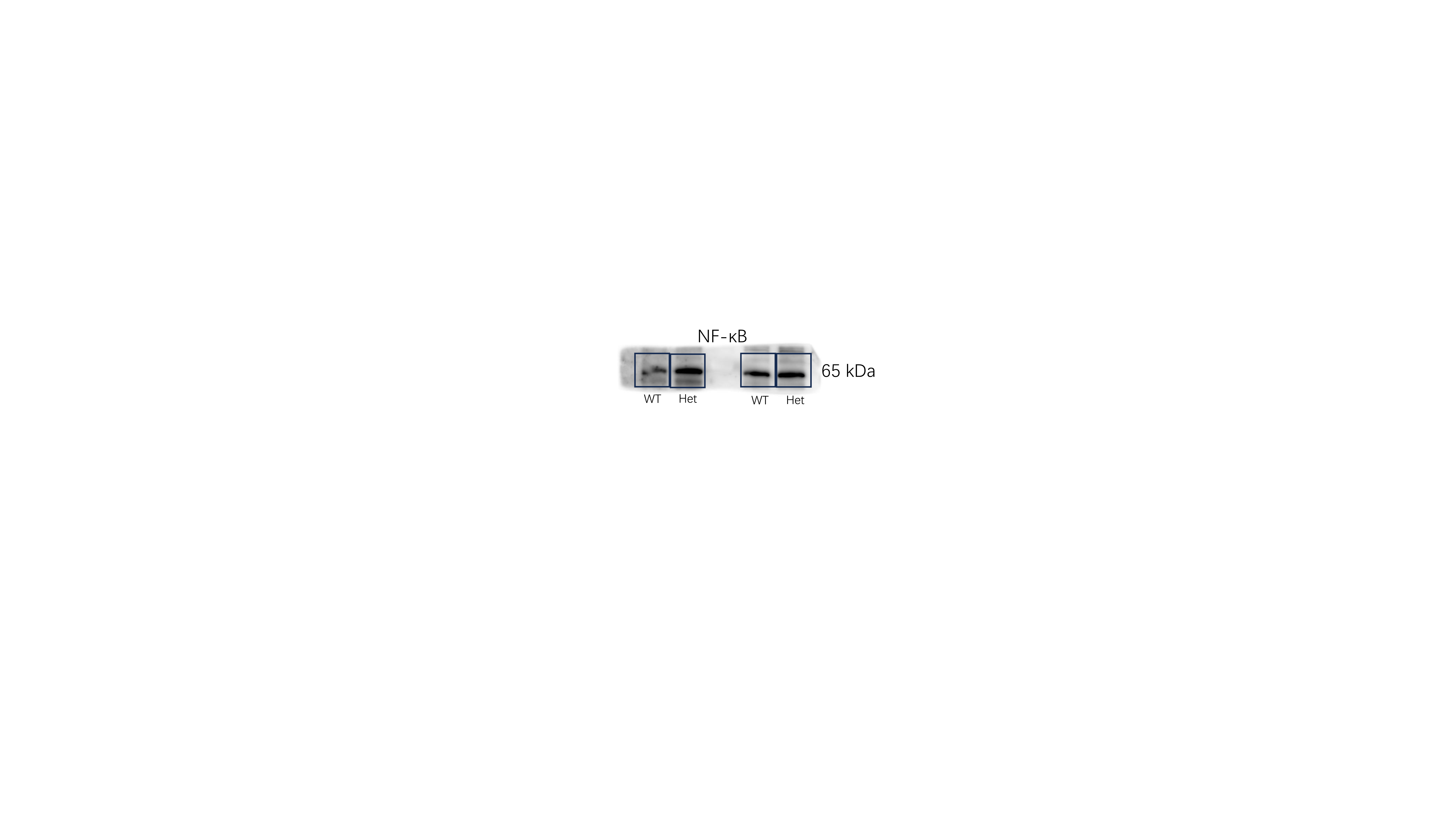

Supplement: Figure 6—source data 12. [file elife-91289-fig6-data12.zip › Figure 6-source data 12/NF-κB-1.TIF]

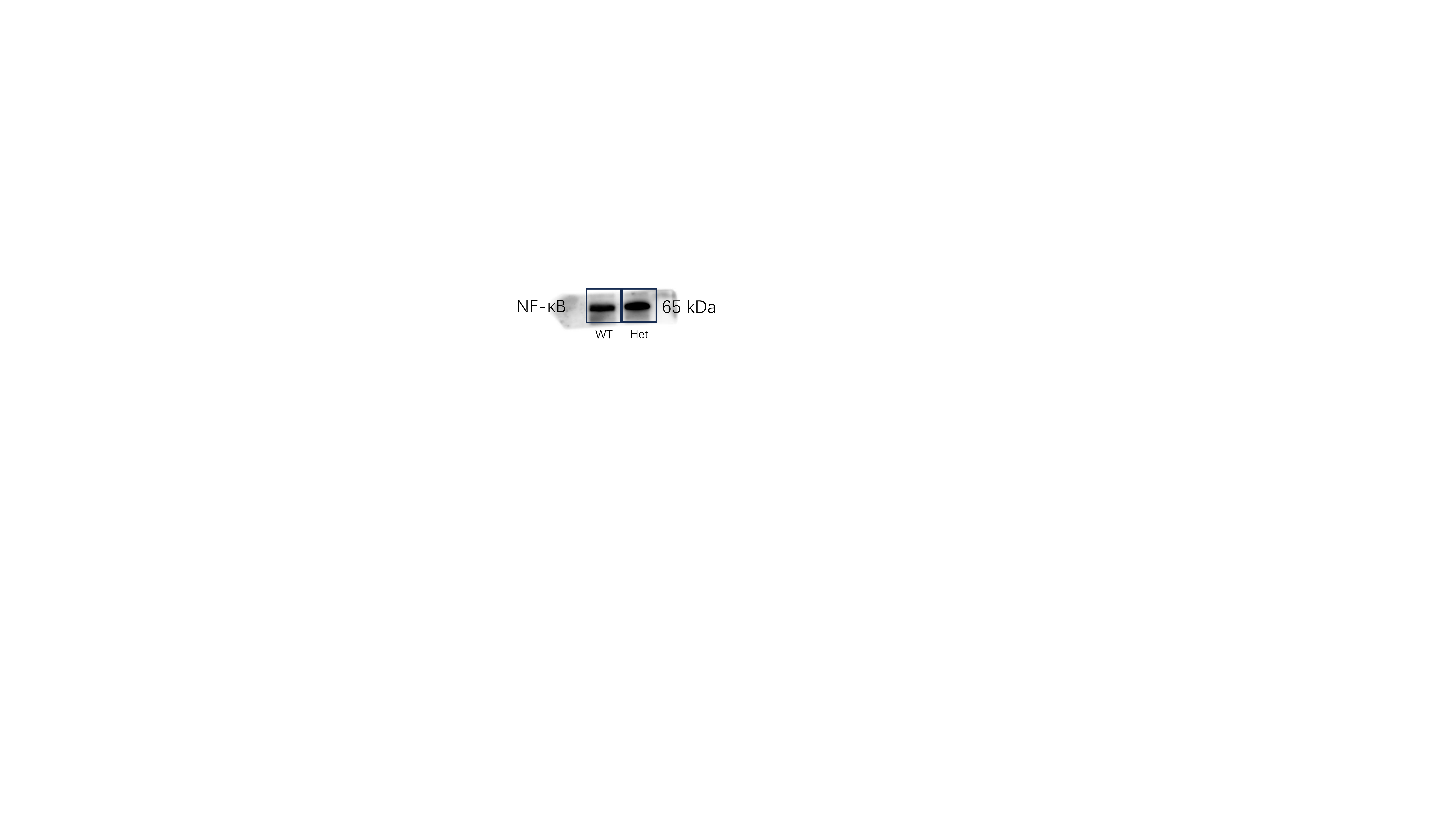

Supplement: Figure 6—source data 12. [file elife-91289-fig6-data12.zip › Figure 6-source data 12/NF-κB-2.TIF]

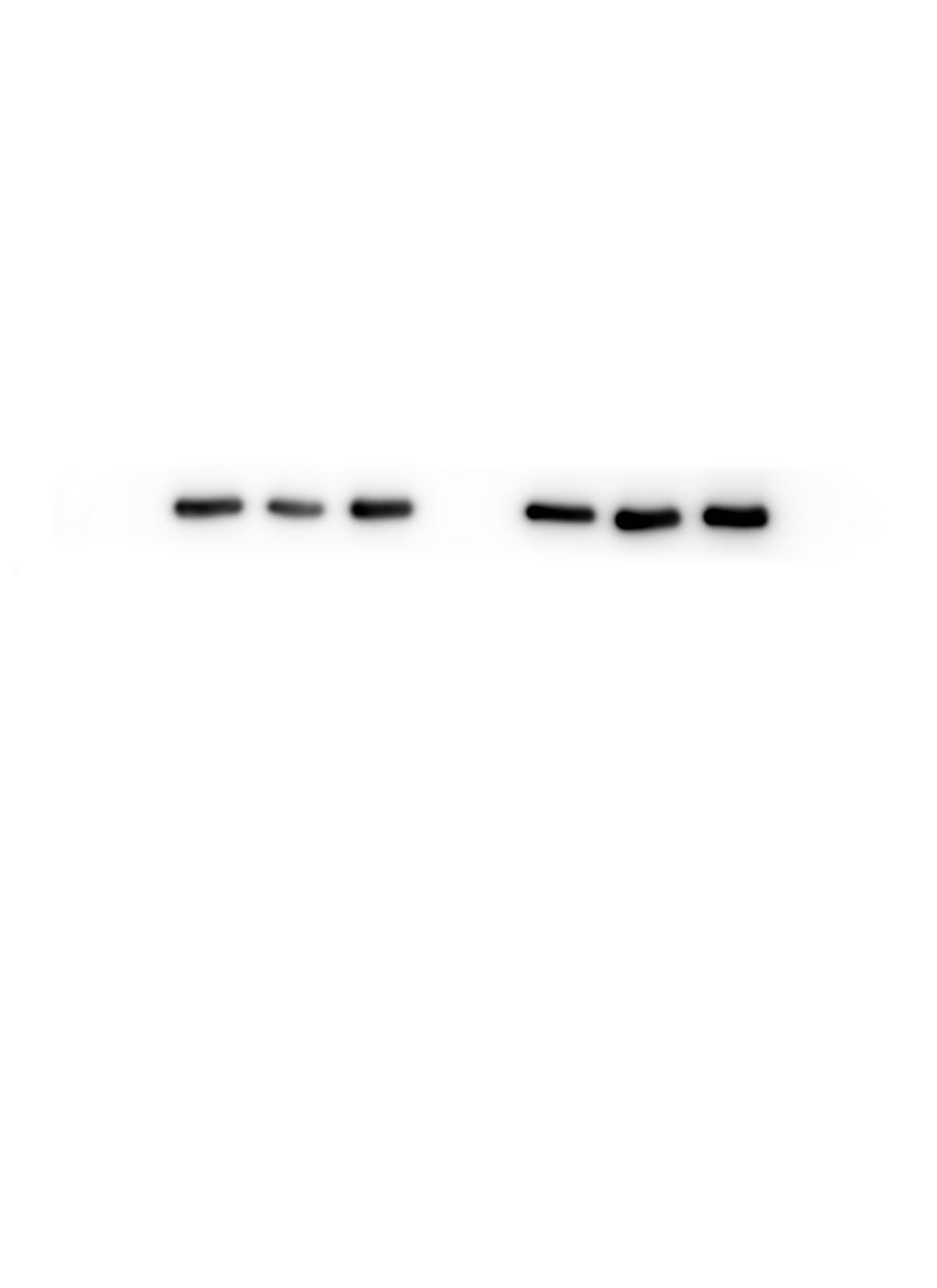

Supplement: Figure 7—source data 1. [file elife-91289-fig7-data1.zip › Figure 7-source data 1/GAPDH-2.jpg]

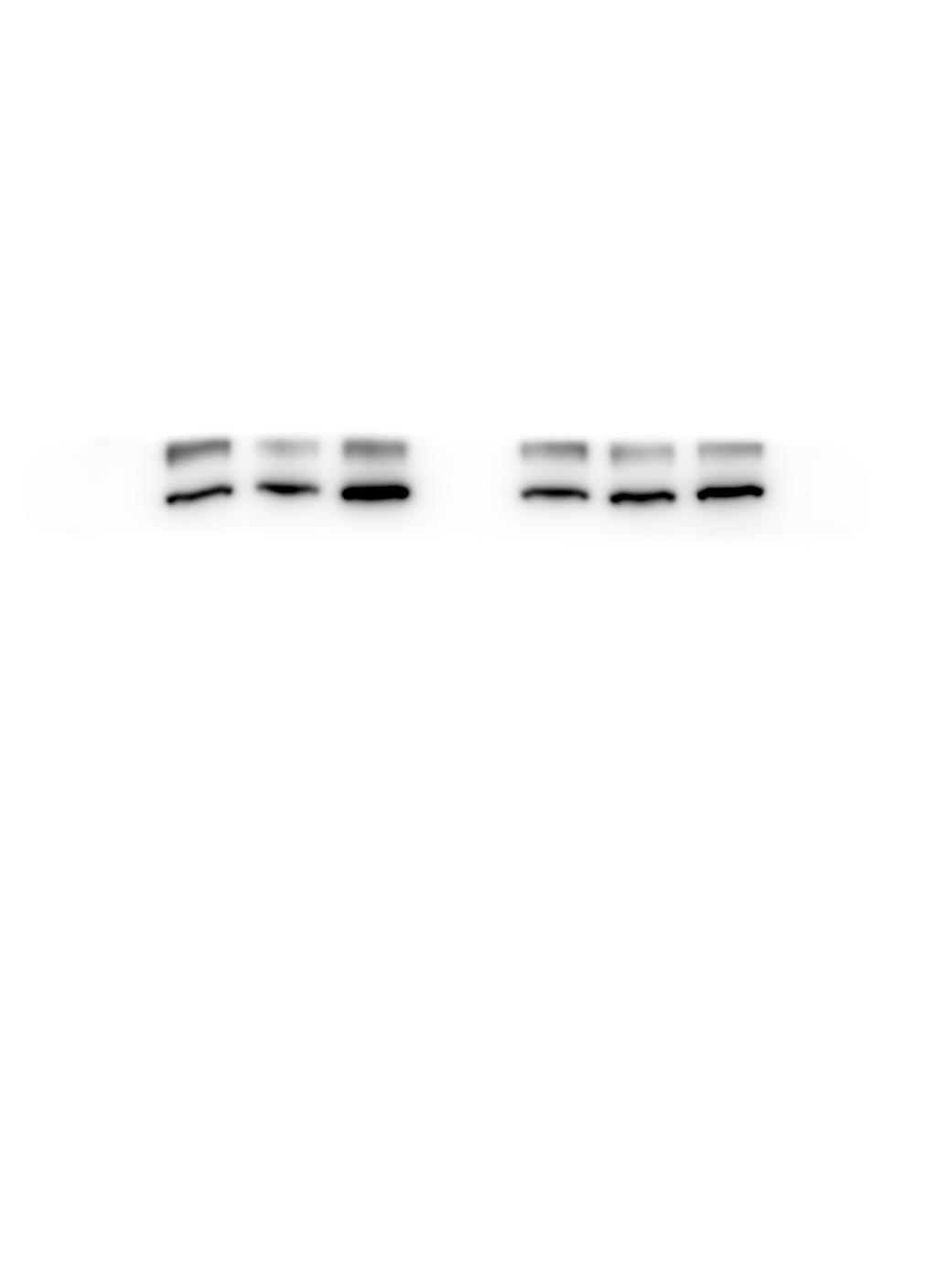

Supplement: Figure 7—source data 1. [file elife-91289-fig7-data1.zip › Figure 7-source data 1/MMP-2-2.jpg]

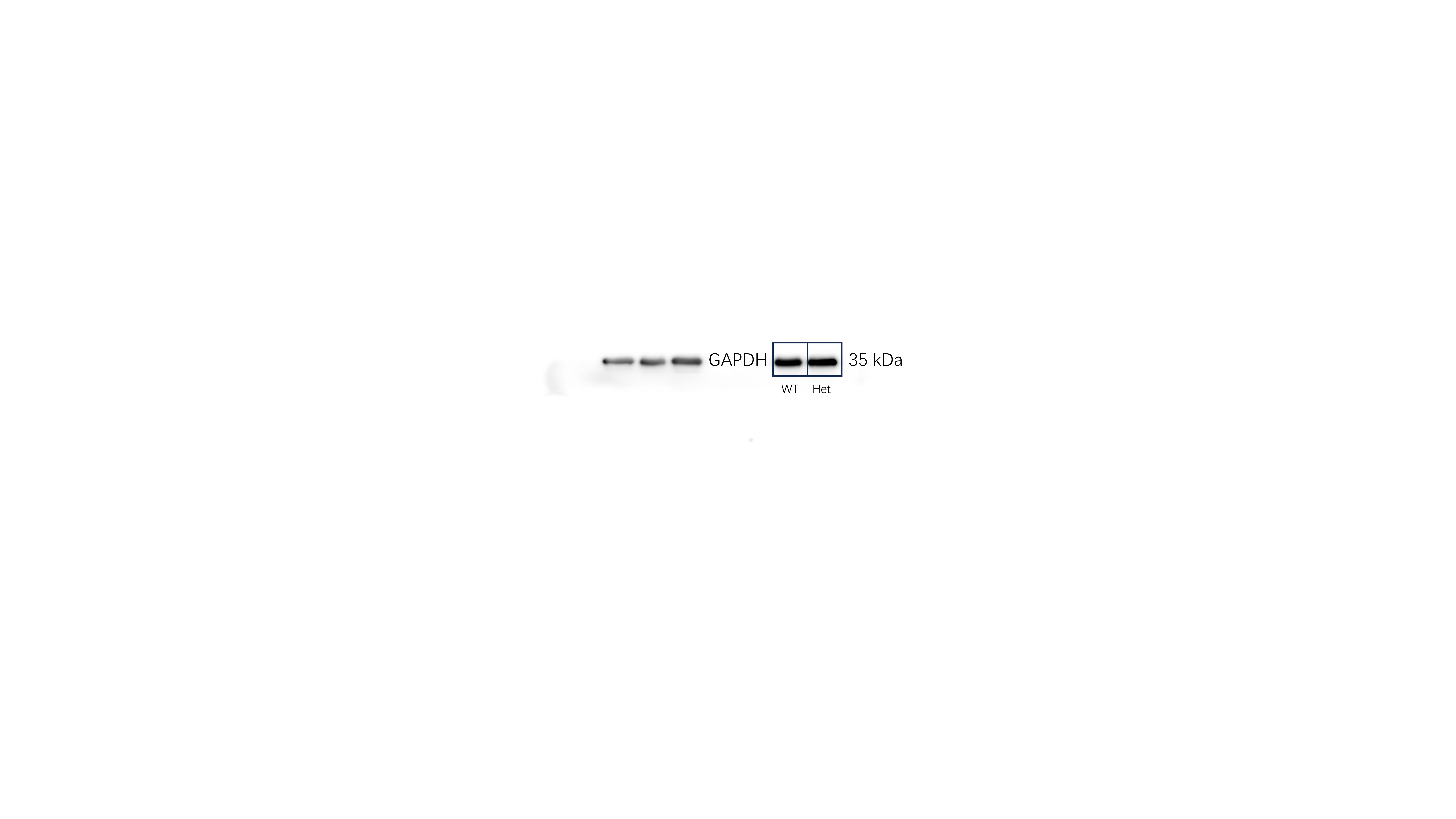

Supplement: Figure 7—source data 2. [file elife-91289-fig7-data2.zip › Figure 7-source data 2/GAPDH-1.tif]

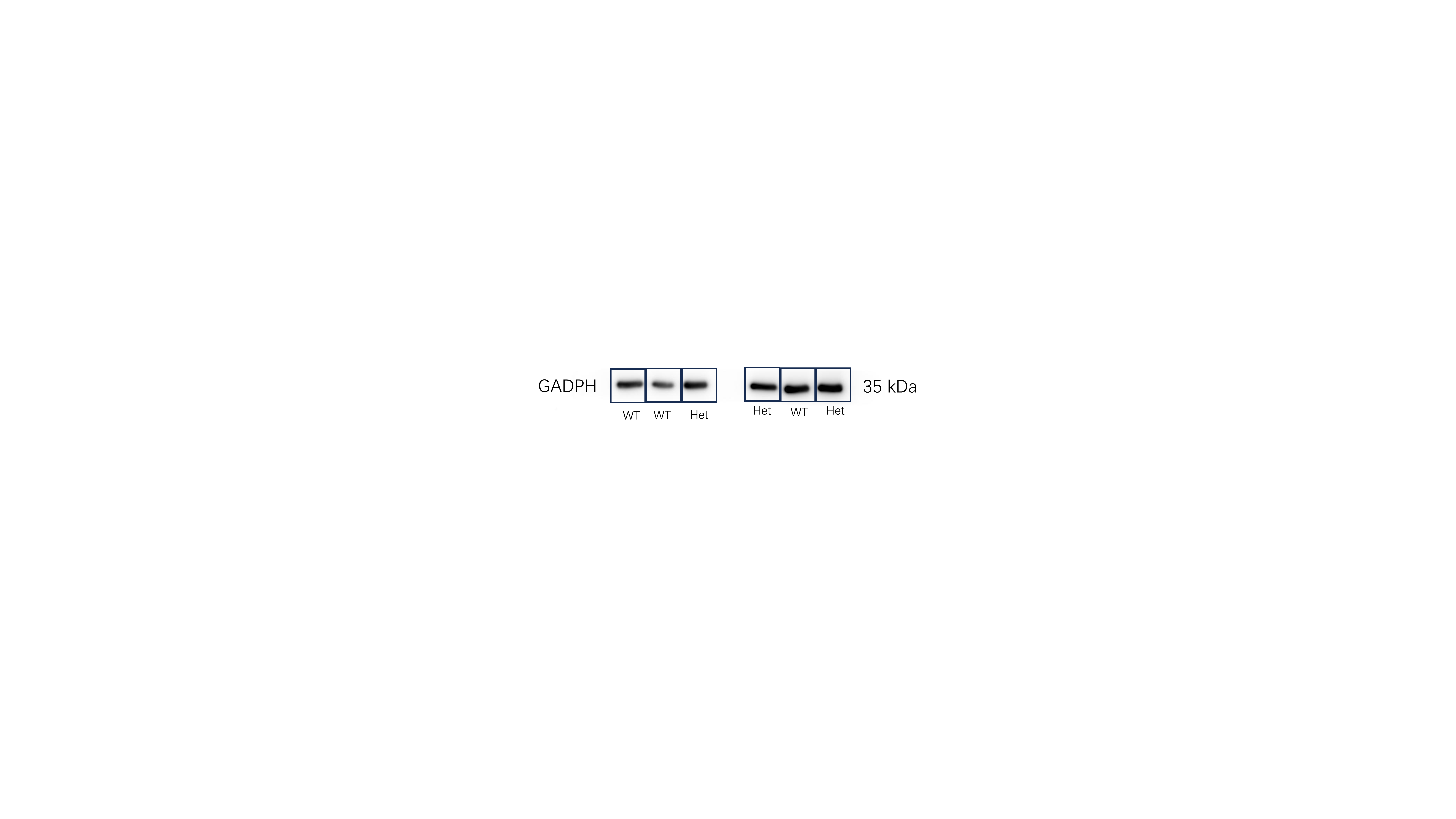

Supplement: Figure 7—source data 2. [file elife-91289-fig7-data2.zip › Figure 7-source data 2/GAPDH-2.tif]

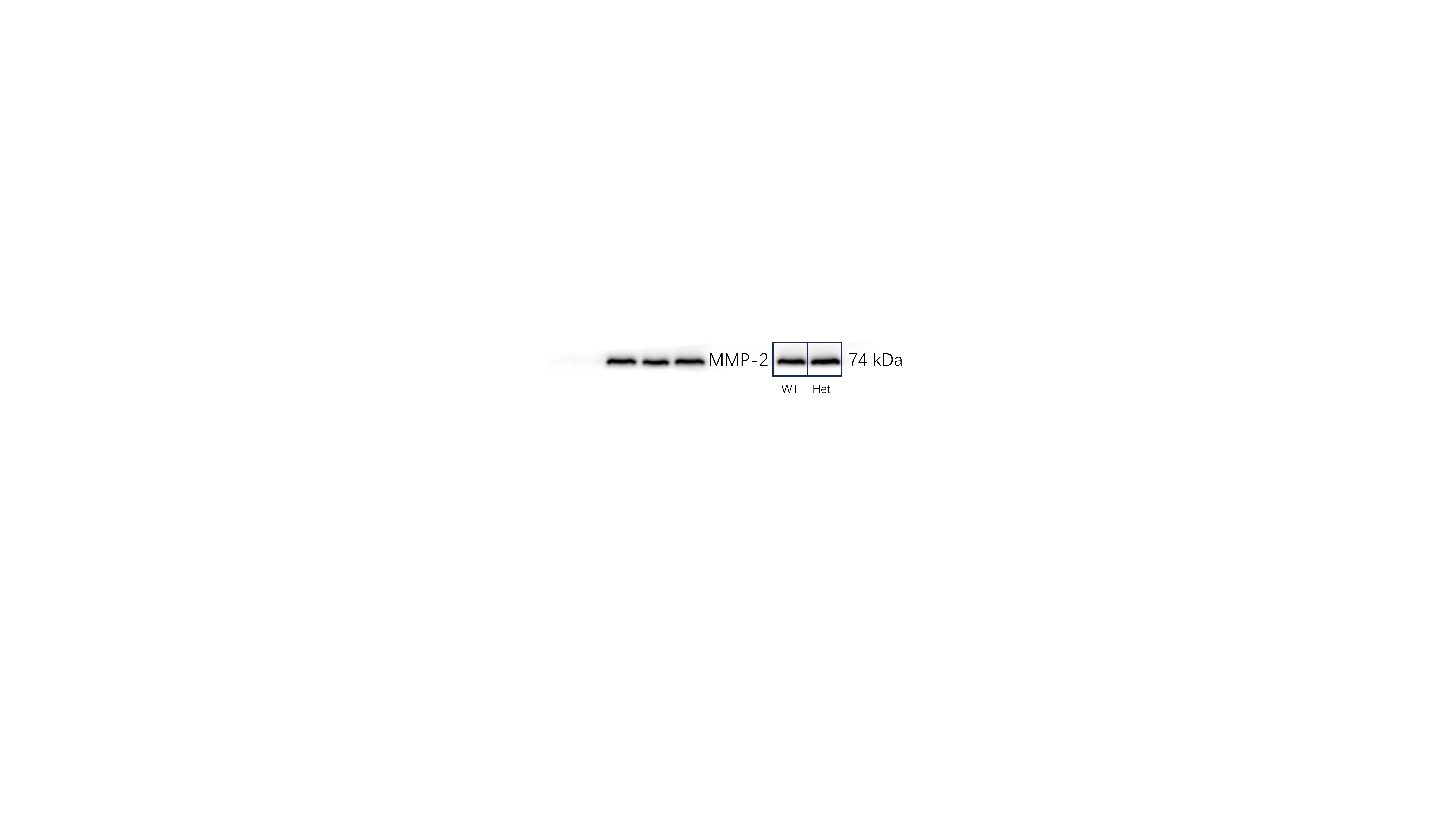

Supplement: Figure 7—source data 2. [file elife-91289-fig7-data2.zip › Figure 7-source data 2/MMP-2-1.tif]

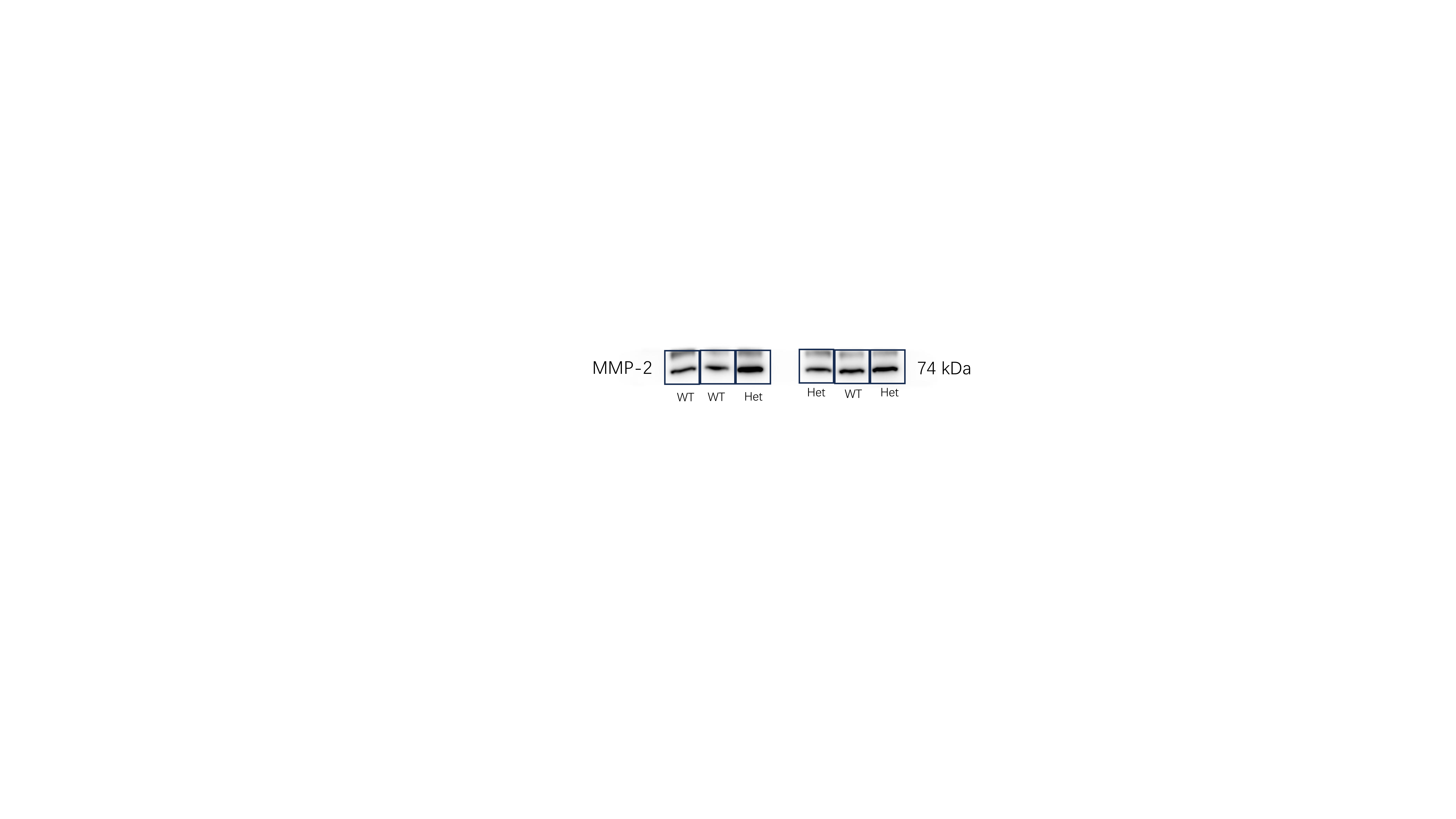

Supplement: Figure 7—source data 2. [file elife-91289-fig7-data2.zip › Figure 7-source data 2/MMP-2-2.tif]

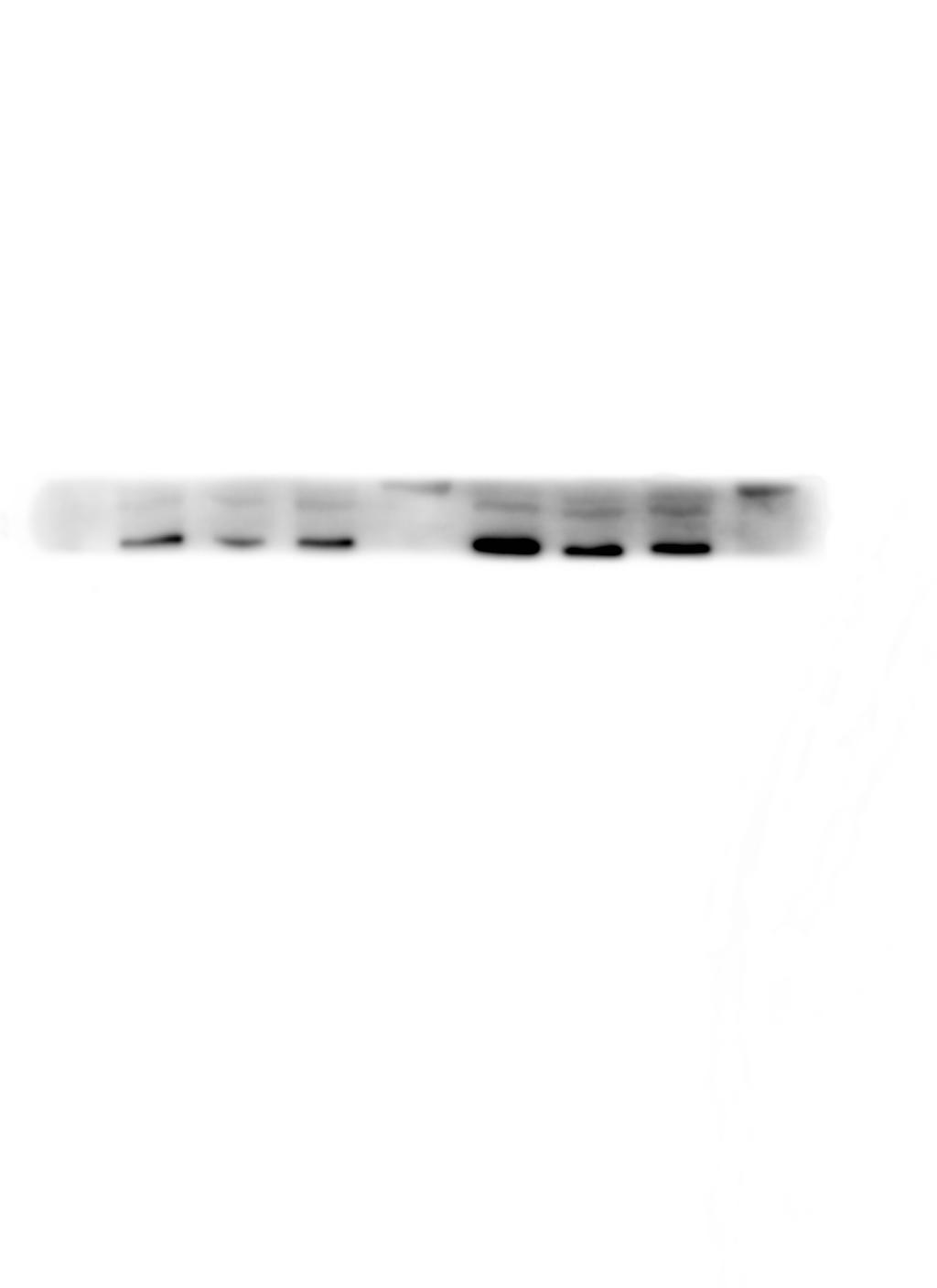

Supplement: Figure 7—source data 3. [file elife-91289-fig7-data3.zip › Figure 7-source data 3/TGF-β1.jpg]

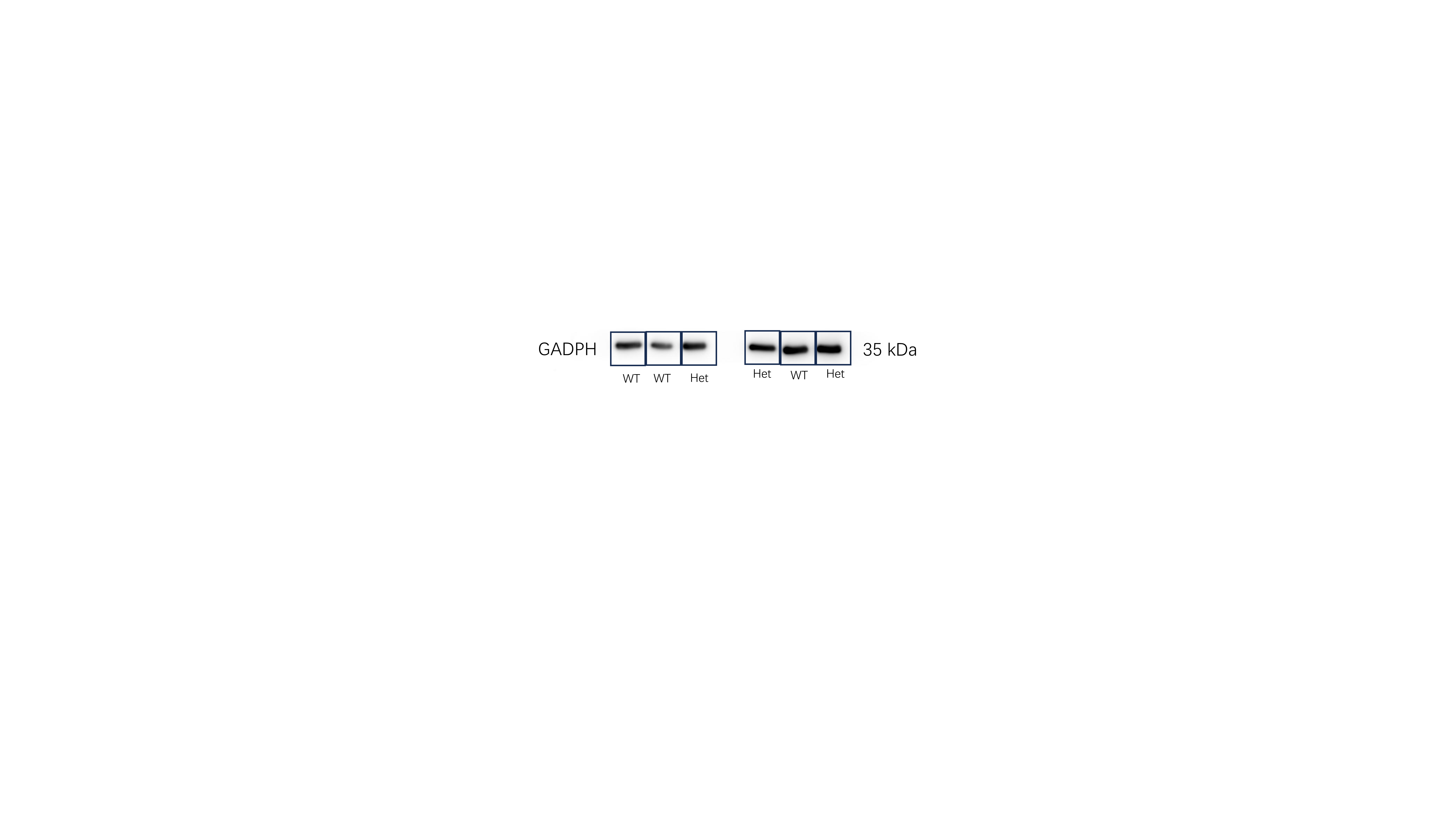

Supplement: Figure 7—source data 4. [file elife-91289-fig7-data4.zip › Figure 7-source data 4/GAPDH.tif]

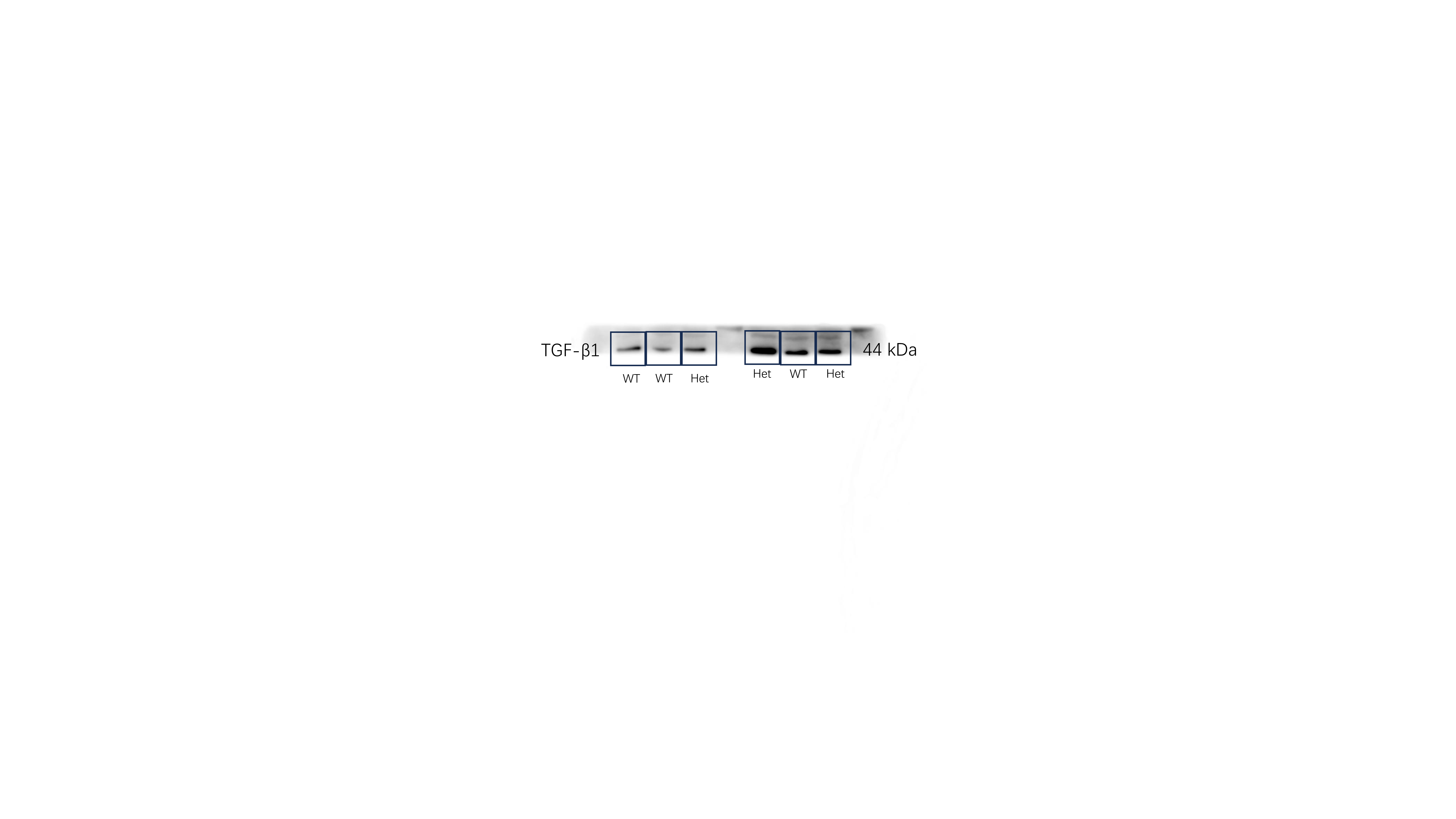

Supplement: Figure 7—source data 4. [file elife-91289-fig7-data4.zip › Figure 7-source data 4/TGF-β1.tif]
