# Supplementary material for: ZC3H11A mutations cause high myopia by triggering PI3K-AKT and NF-κB-mediated signaling pathway in humans and mice
Source: eLife. 2025 Aug 27;12:RP91289. doi: 10.7554/eLife.91289 (PMC12387752; doi:10.7554/eLife.91289)
Supplement: Supplementary file 1. [file elife-91289-supp1.docx]

**Supplementary File 1. Sequence of oligonucleotides**

| Gene | Sequence |
| --- | --- |
| ZC3H11A | Forward: CTAAACTGCGCTTTCCATCACA  Reserve: CGTTGATTACAACAGGCGGAT |
| PI3K | Forward: TGGGACCTTTTTGGTACGAGA  Reserve: AGCTAAAGACTCATTCCGGTAGT |
| AKT | Forward: CCTTTATTGGCTACAAGGAACGG  Reserve: GAAGGTGCGCTCAATGACTG |
| IκBα | Forward: TGAAGGACGAGGAGTACGAGC  Reserve: TGCAGGAACGAGTCTCCGT |
| NF-κB | Forward: GGGGCCTGCAAAGGTTATC  Reserve: TGCTGTTACGGTGCATACCC |
| TGF-β1 | Forward: GTAACGCCAGCCAGGAATTGTTGCTA  Reserve: CTTCAATACGTCAGACATTCGGG |
| IL-6 | Forward: CTGCAAGAGACTTCCATCCAG  Reserve: AGTGGTATAGACAGGTCTGTTGG |
| MMP-2 | Forward: CAAGTTCCCCGGCGATGTC  Reserve: TTCTGGTCAAGGTCACCTGTC |
| GAPDH | Forward: AGGTCGGTGTGAACGGCTTTG  Reserve: TGTAGACCATCTAGTTGAGGTCA |

The mRNA expression levels were analysed by the comparative $2^{-\Delta\Delta}$ Ct method and normalised using GAPDH.
